# DNA-Based Networks Formed by Coordination Cross-Linking of DNA with Metal–Organic Polyhedra: From Gels to Aerogels to Hydrogels

**DOI:** 10.1021/jacs.5c03934

**Published:** 2025-05-01

**Authors:** Laura Hernández-López, Akim Khobotov-Bakishev, Alba Cortés-Martínez, Eduard Garrido-Ribó, Partha Samanta, Sergio Royuela, Félix Zamora, Daniel Maspoch, Arnau Carné-Sánchez

**Affiliations:** † Catalan Institute of Nanoscience and Nanotechnology (ICN2), CSIC, and The Barcelona Institute of Science and Technology, Campus UAB, Bellaterra 08193, Spain; ‡ Departament de Química, Facultat de Ciències, Universitat Autònoma de Barcelona, Bellaterra 08193, Spain; § Departamento de Química Inorgánica, Universidad Autónoma de Madrid, Ciudad Universitaria de Cantoblanco, Madrid 28049, Spain; ∥ ICREA, Pg. Lluís Companys 23, Barcelona 08010, Spain

## Abstract

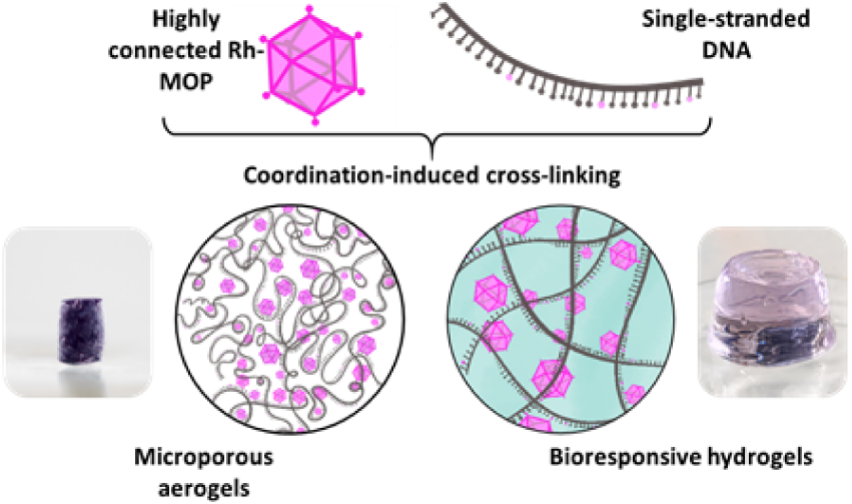

Herein, we introduce a supramolecular method to form DNA-based networks by cross-linking DNA with Rh(II)-based metal–organic polyhedra (MOPs), which entails coordination of DNA to the exohedral Rh(II) axial sites of the MOP. The resultant highly connected networks can then be processed into gels, porous aerogels, or hydrogels, exhibiting properties suitable for pollutant removal and drug release.

## Introduction

DNA is an appealing natural polymer due to its unique capability to encode biological and chemical information on a biocompatible polymeric matrix, and to its high environmental responsiveness.^1−3^ These features can be harnessed to develop functional materials for applications such as molecular delivery,^4^ biorecognition and environmental protection.^5^ The synthesis of DNA-based materials involves the cross-linkage of DNA into networks, which can be shaped into macroscopic functional objects, such as hydrogels and aerogels.^6,7^ A common strategy for cross-linking DNA into purely DNA-based networks relies on the base pairing of complementary strands.^8^ The reversible nature of this interaction yields responsive materials whose physicochemical and mechanical properties can be further enhanced when the network includes self-assembled structures of DNA^9^ such as i-motifs,^10^ G-quadruplexes,^11^ DNA triplex structures^12^ or aptamers.^13^ Alternatively, the diversity of functional groups in DNA confers it with rich latent reactivity, allowing for the cross-linking of DNA strands with other materials through covalent bonding (between nucleobases),^14^ hydrophobic interactions,^15^ H-bonding,^16^ physical entanglement,^17^ coordinative bonds^18^ and electrostatic interactions.^19^

Despite these advances, current synthetic strategies for DNA networks rely on cross-linkers with low connectivity (e.g., DNA junctions or end-functionalized linear polymers) or multivalent nodes with undefined connectivity due to their inherent polydispersity (e.g., polycationic polymers and inorganic nanoparticles). These limitations hinder the controlled synthesis of highly connected DNA networks with enhanced mechanical properties and stability. In contrast, the field of reticular materials has addressed this challenge through the supermolecular building block (SBB) approach,^20^ which enables the design and construction of highly connected porous networks by incorporating in situ-formed or preassembled metal–organic (or organic) polyhedra (MOPs) as highly connected structural nodes.^21,22^

Herein, we introduce the SBB concept to the synthesis of DNA networks by using MOPs as molecularly precise, nanoscopic nodes with high connectivity. Specifically, we employ the robust, anionic, and water-soluble Rh(II)-based cuboctahedral MOP with the formula Na_24_[Rh_24_O-BDC_24_] (hereafter referred to as ONa-RhMOP, where O-BDC is 5-oxido-1,3-benzendicarboxylate) to facilitate the three-dimensional cross-linking of DNA into gels, aerogels, and hydrogels.^23^ This MOP exhibits 24 sodium phenoxide groups on its external surface, conferring it with water solubility, and 12 Rh(II) axial sites that are suitable for coordinatively interacting with other species. In fact, this type of MOP has recently been employed as a 3D, 12-connected building block to construct amorphous and crystalline coordinative networks through connection with organic linkers or molecular clusters via the 12 Rh(II) axial sites.^24,25^ Here, we propose that the high connectivity and molecular precision of ONa-RhMOP can be exploited to create highly cross-linked DNA networks, facilitating the transition from gels to robust aerogels and hydrogels. Accordingly, we selected genomic salmon sperm DNA with over 9000 bp (Figure S1), a surrogate of biomass-derived DNA,^26^ as a sustainable, renewable and abundant source of DNA. The DNA-MOP networks displayed excellent mechanical properties in both the gel (storage modulus >10 KPa) and aerogel (Young’s modulus >45 MPa) forms, which can be attributed to the high connectivity of the network. Additionally, the incorporation of MOP into the DNA network induced permanent microporosity in the aerogel, making it the first DNA network to exhibit this property. Furthermore, the hydrogel form demonstrated adsorptive capacity and triggered release for guest molecules, expanding the potential of DNA-based materials as robust adsorbents for both solid-state and liquid-phase applications.

## Results and Discussion

### Synthesis and Formation Mechanism of DNA-MOP Gel Networks

**Figure 1:**
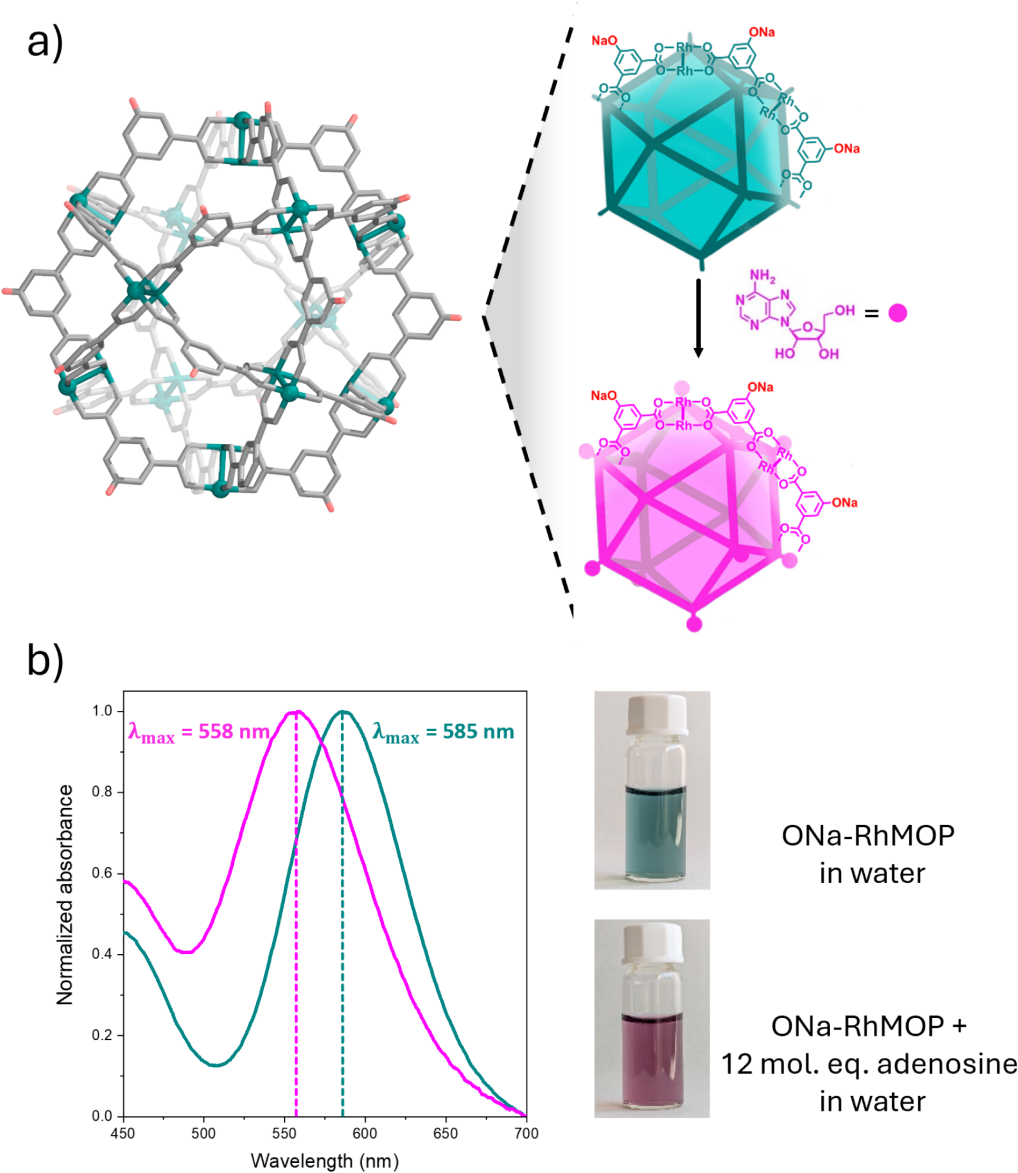
(a) Structure of ONa-RhMOP (left), and schematics of its coordination with adenosine (right). (b) UV–vis spectra and photographs of ONa-RhMOP before (green) and after (pink) its coordination to adenosine.

We began by analyzing the coordinative interactions of the exposed Rh(II) axial sites of ONa-RhMOP with the N-donor atoms of the four nucleosides present in DNA. To this end, ONa-RhMOP was separately combined with 12 mol equivalents (mol. eq.) of each of the nucleosides in basic water. UV–vis spectroscopy revealed that only adenosine coordinates to ONa-RhMOP through its N-donor atoms, as evidenced by a shift in the π* → σ* transition of Rh–Rh bonds (λ_max_) from 585 to 558 nm (Figure 1).^27^ Steric hindrance around the N-donor atoms of the other three nucleosides precludes their coordinative interaction with ONa-RhMOP (Figure S2).^28^ These results align with previous reports showing the Rh(II) paddlewheel cluster’s preference for adenine over other nucleobases, as demonstrated by computational modeling and experimental studies. In all cases, the preferred coordination involves the axial Rh(II) site binding to basic nitrogen atoms.^29−31^

**Figure 2:**
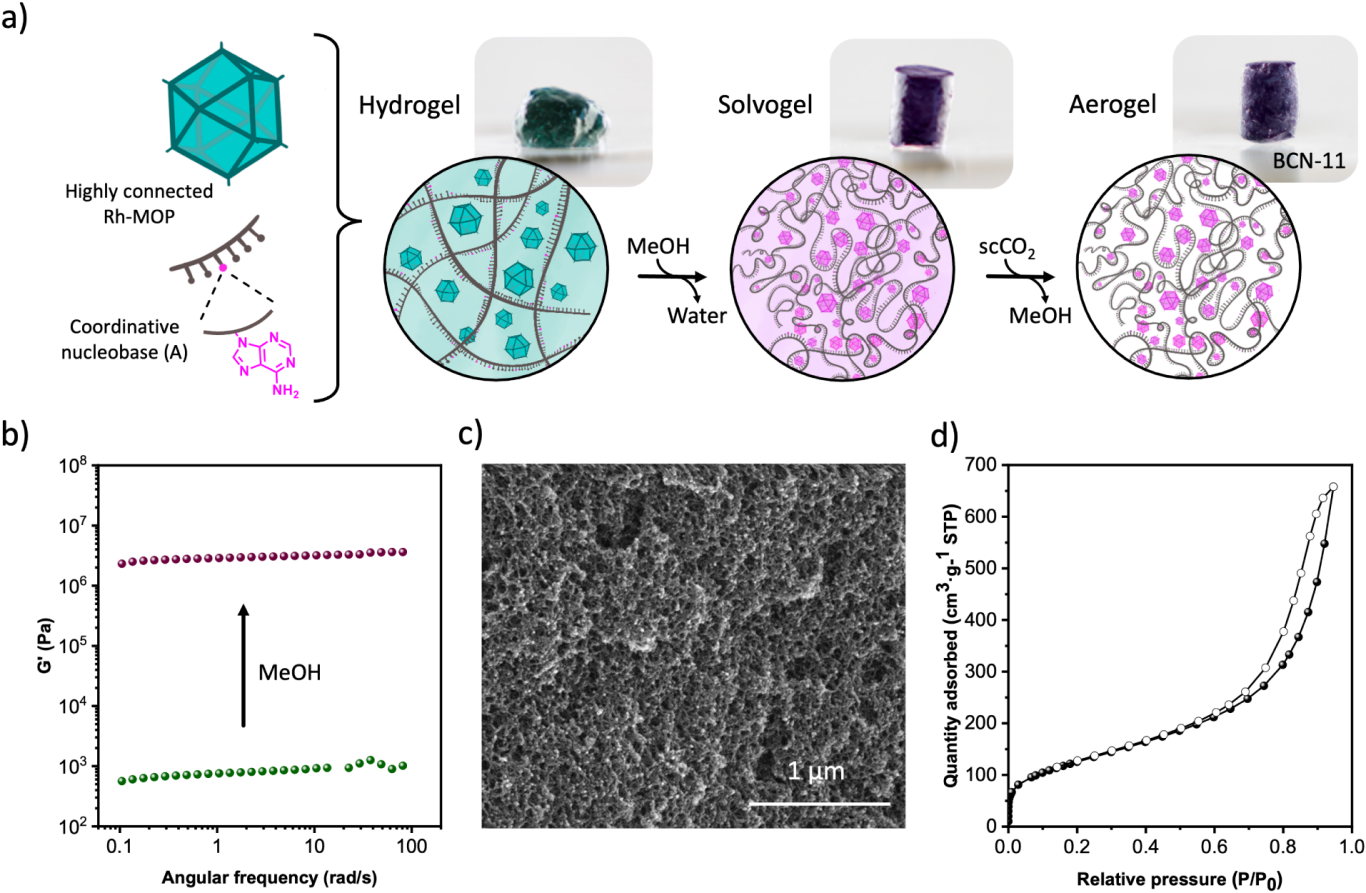
(a) Schematic of the formation mechanism of BCN-11. (b) Rheology measurements of DNA-MOP networks before (green) and after (purple) methanol exchange. (c) FE-SEM image of BCN-11_42. (d) N_2_-sorption isotherm for BCN-11_42.

Once we had confirmed adenosine as a potential coordinative binding site, we then reacted ONa-RhMOP with DNA in an aqueous solution containing NaOH. Basic conditions were selected to form single-stranded DNA with all adenosine moieties available to react with the MOP. Mixing the two components immediately afforded a green gel with a frequency-independent storage modulus (G’) of 0.76 kPa, as determined by rheological analysis (Figure 2b). However, solid-state UV–vis analysis of the DNA-MOP hydrogel showed that λ_max_ remained constant at 586 nm throughout the gel formation reaction, indicating the absence of coordinative interactions between the DNA and the MOP (Figure S3). We reasoned that H-bonding between ONa-RhMOP and DNA caused gelification. FT-IR spectrum of the lyophilized gel supported this, showing a 12–14 cm^–1^ decrease in carbonyl and phosphate peaks, indicating that these moieties participate in H-bonding interactions, such as carboxylate-amine or phosphonate-hydroxyl interactions (Figure S4).^16,32^ FE-SEM analysis of the lyophilized gel revealed a macroporous network in which DNA and ONa-RhMOP are homogeneously dispersed, as confirmed by EDX analysis (Figure S5).

We ascribed the lack of coordinative interactions in the DNA-MOP hydrogel to the electrostatic repulsion between the two negatively charged constituents of the gel. One strategy to screen electrostatic repulsion is the use of solvents with low dielectric constant. Thus, the DNA-MOP hydrogel was incubated in methanol to leverage the latent coordination potential of DNA. Upon immersion in methanol for 24 h, the DNA-MOP hydrogel changed color, from green to purple, which indicated the coordinative interaction between the DNA and the Rh(II) axial sites of ONa-RhMOP. Solid-state UV–vis of the methanol exchanged solvogel confirmed the coordination of DNA to the ONa-RhMOP through N-donor groups as λ_max_ shifted from 586 to 566 nm (Figure S6). Furthermore, the methanol exchange process also induced densification of the network by decreasing the volume of the gel by 65% due to the alcohol-induced condensation of DNA (Figure S7).^33^ These changes in the network induced an increase of the mechanical properties of the gel, as G’ increased from 0.76 to 2880 kPa upon incubation in methanol (Figure 2b). An increase of G’ by a factor of 3790 can not only be ascribed to the densification of DNA in apolar solvents but also to the strong DNA-MOP coordination interaction.^34^

Therefore, we propose that the coordinative DNA-MOP network forms in two steps (Figure 2a). In the first step, ONa-RhMOP establishes hydrogen-bonding interactions with DNA, generating a network in which mutual electrostatic repulsion inhibits adenosine coordination to the Rh(II) axial sites. In the second step, methanol addition to the H-bonded network induces simultaneous DNA condensation and coordination to the ONa-RhMOP surface. To validate this hypothesis, we replicated this two-step process using adenosine monophosphate as a model compound. Similar to DNA, adenosine monophosphate does not coordinate to ONa-RhMOP through its N atoms in basic water, as confirmed by UV–vis spectroscopy, which showed no shift in λ_max_. However, after exposing the reaction mixture to methanol, λ_max_ shifted from 590 to 570 nm indicating that the exohedral Rh(II) axial sites are coordinated to N atoms, as the O-donor groups present in the molecule (e.g., phosphate groups) do not induce a blue shift in the λ_max_ of the Rh(II) paddlewheel (Figure S8).^35−37^ Therefore, once the Rh–N coordination between adenosine monophosphate and Rh(II) is established in methanol, it remains stable even upon re-exposing the mixture to basic water, as evidenced by UV–vis and NMR spectra (Figures S29–S31). Thus, the initial electrostatic repulsion acts as an energy barrier to coordination, but once overcome, the interaction is preserved in water.

Remarkably, the above-described two-step mechanism was independent of the initial amount of ONa-RhMOP. Indeed, we synthesized a series of solvogels by systematically varying the mass percentage (% w/w, defined as (w_ONa-RhMOP_/w_(ONa-RhMOP+DNA)_) × 100) of ONa-RhMOP in the reaction mixture from 50% to 14% (w/w). Conversely, when the same reaction was performed with a simple Rh(II) acetate cluster, immediate precipitation of ill-defined, mechanically weak amorphous solids was observed, which could not be further processed or characterized in terms of mechanical properties. These results highlight the importance of the high connectivity and surface negative charge of the MOP in the two-step gelation process described above.

### Synthesis and Characterization of BCN-11 Aerogels

We transformed all DNA-MOP solvogels into aerogels by drying them with supercritical CO_2_. The composition of each of these aerogels was determined by quantifying the amount of Rh and P through inductively coupled plasma-optical emission spectroscopy of the acid-digested samples (Table S1). Thus, the reaction mixtures containing ONa-RhMOP at 14%, 25%, 40% and 50% (w/w) afforded aerogels containing ONa-RhMOP at final concentrations of 13%, 21%, 35%, and 42% (w/w), respectively. This discrepancy is directly proportional to the amount of MOP used in the synthesis, due to the progressive saturation of the networks by MOPs, which leads to leaching of unbound MOP during washing. The coordinative DNA-MOP interactions were preserved in all aerogel networks (hereafter named as BCN-11_XX, where XX denotes the % w/w in the aerogel), as confirmed through solid-state UV–vis (Figure S9). The mechanical properties of all BCN-11 aerogels were measured through uniaxial quasi-static compression tests, which revealed that the amount of MOP in the network modulates the stiffness of the obtained aerogel. Thus, BCN-11_13 and BCN-11_21 are plastic materials with Young’s modulus values of 14.8 and 30.5 MPa, respectively. Conversely, BCN-11_35 and BCN-11_42 are stiffer brittle monoliths with Young’s modulus values of 40.9 and 46.3 MPa, respectively, an order of magnitude higher than current DNA-based aerogels (Figure S10).^38−40^

Next, we characterized the microstructure of the BCN-11 series by FE-SEM, which revealed a colloidal network of fused nanoparticles of sizes of ca. 25 nm (Figures 2c and S11–S14). This network was thermally stable up to ca. 225 °C, enabling thermal activation before the gas-sorption experiments (Figure S15). Measurements of N_2_ sorption at 77 K revealed that all aerogels are porous to N_2_, exhibiting a type IV isotherm characteristic of aerogels having a broad range of pore sizes (Figures 2d and S16). The BET surface area of BCN-11 aerogels increased with the concentration of MOP in the network, ranging from 177 m^2^·g^–1^ for BCN-11_13 to 452 m^2^·g^–1^ for BCN-11_42 (Figures S17–24). The micropore (V_μ_) and total pore volumes (V_t_) of the four aerogels were as follows: BCN-11_13 (0.085 and 0.52 cm^3^/g), BCN-11_21 (0.19 and 1.24 cm^3^/g), BCN-11_35 (0.21 and 1.14 cm^3^/g), and BCN-11_42 (0.22 and 1.02 cm^3^/g). Pore-size distribution (PSD) analysis consistently revealed the presence of micropores (pore sizes: 1.4 and 1.7 nm) and mesopores (pore size: *ca*. 4 nm) in the colloidal network (Figure S25). The micropore size determined by PSD is larger than the inner MOP cavity (0.6 nm),^41^ indicating that N_2_ cannot access it due to the steric hindrance from the DNA attached to the MOP surface. However, the MOP cross-linking unit structures DNA into a microporous network that also generates mesopores through hierarchical organization of the colloidal particles, becoming the first example of a permanently porous DNA network. The porosity of BCN-11 was also confirmed through CO_2_ gas-sorption measurements at 298 K, which, for BCN-11_42, revealed a maximum uptake of 1.13 mmol·g^–1^ at 1 bar (Figure S26).

### Synthesis, Mechanical Properties and Stability Against DNAse I of BCN-11 Derived Hydrogels

**Figure 3:**
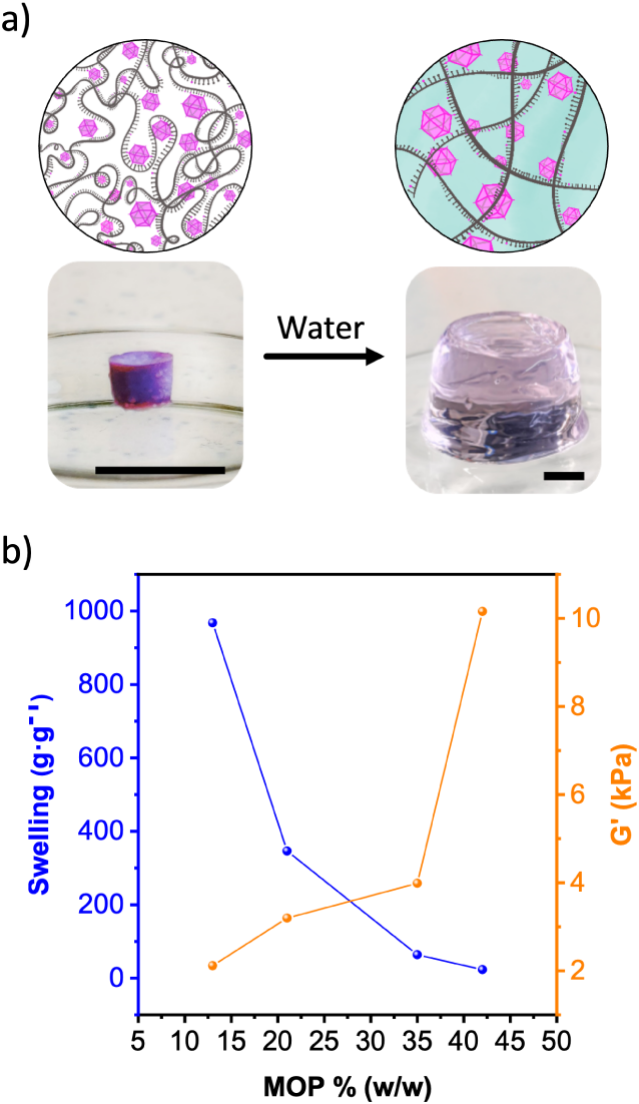
(a) Photographs and schematic of the hydration of BCN-11. Scale bars: 1 cm. (b) Swelling and storage moduli of hydrogels derived from BCN-11.

A common feature of polymeric networks is their ease of conversion from xerogels or aerogels to hydrogels upon immersion in water.^42,43^ However, this phenomenon is much less common for permanently porous aerogels, which are brittle and decompose or maintain their solid-like behavior upon incubation in water, as they are generally assembled from low molecular weight linkers.^44^ Thus, considering the polymeric nature of DNA, we aimed to investigate the behavior of BCN-11 aerogels upon incubation in water. All four BCN-11 aerogels transformed into stable hydrogels upon incubation in water for 24 h, without any leaching of ONa-RhMOP or DNA (Figures 3a, S27 and Table S2). Furthermore, the coordinative DNA-MOP interaction was preserved in all cases, as evidenced by solid-state UV–vis (Figure S28). This behavior is consistent with the experiments performed with model nucleotides, in which the selective N–Rh coordinative interaction established by adenosine monophosphate in methanol was preserved in water, as evidenced by UV–vis spectroscopy and Diffusion-Ordered Spectroscopy (DOSY) NMR analysis (Figures S29–S37). The swelling ratio (g/g) of the hydrogels was strongly dependent on the % w/w, increasing from 22 for BCN-11_42 to 968 for BCN-11_13 (Figure 3b). Conversely, the robustness of the hydrogels followed the inverse trend, as G’ decreased from 10.1 kPa for BCN-11_42 to 2.1 KPa for BCN-11_13 (Figures 3b and S38). These values of storage modulus are significantly higher than most hydrogels made with synthetic DNA that rarely reach 1 KPa.^45^ Notably, BCN-11 derived hydrogels also show superior stiffness when compared to hydrogels made from long genomic DNA that display values of G’ in the range of 0.01 KPa–1 KPa (Table S3).^46,47^ We ascribe the excellent mechanical properties of the BCN-11 hydrogel network to the high connectivity of the ONa-RhMOP and the strength of the Rh–N coordination.

The rheology and swelling measurements indicate that the ONa-RhMOP node controls the density and entanglement of DNA within the network. We envisioned that the response of BCN-11-derived hydrogels to DNase I could be modulated since these two parameters are crucial for recognition of DNA by DNase I.^48^ Therefore, the stability of all four BCN-11-derived hydrogels against DNase I was assessed by incubating the hydrogels in a solution containing the enzyme for 24 h. We found that BCN-11_35 and BCN-11_42 hydrogels remained stable upon incubation in DNase I, whereas hydrogels with a lower content of MOP degraded into noncytotoxic byproducts under the same conditions (Figures S27 and S39). We attribute the stability of BCN-11_35 and BCN-11_42 hydrogels against DNase I to the coordinative linkages between DNA and the 3D MOP cross-linking units, which entangle DNA into dense conformations, effectively shielding it from enzymatic degradation. As the number of MOP nodes increases, the protected regions within the DNA network expand, reaching a threshold at 35% w/w, beyond which the entire network becomes resistant to DNase I. We compared this behavior to that of histones, DNA-binding proteins that are crucial for gene regulation and operate principally by electrostatic interactions. Therefore, the DNA-MOP coordinative cross-linking strategy enables the modulation of the biochemical and mechanical properties of DNA hydrogels.

### BCN-11 Derived Hydrogels as Dual Site Adsorbents for Pollutant Removal

**Figure 4:**
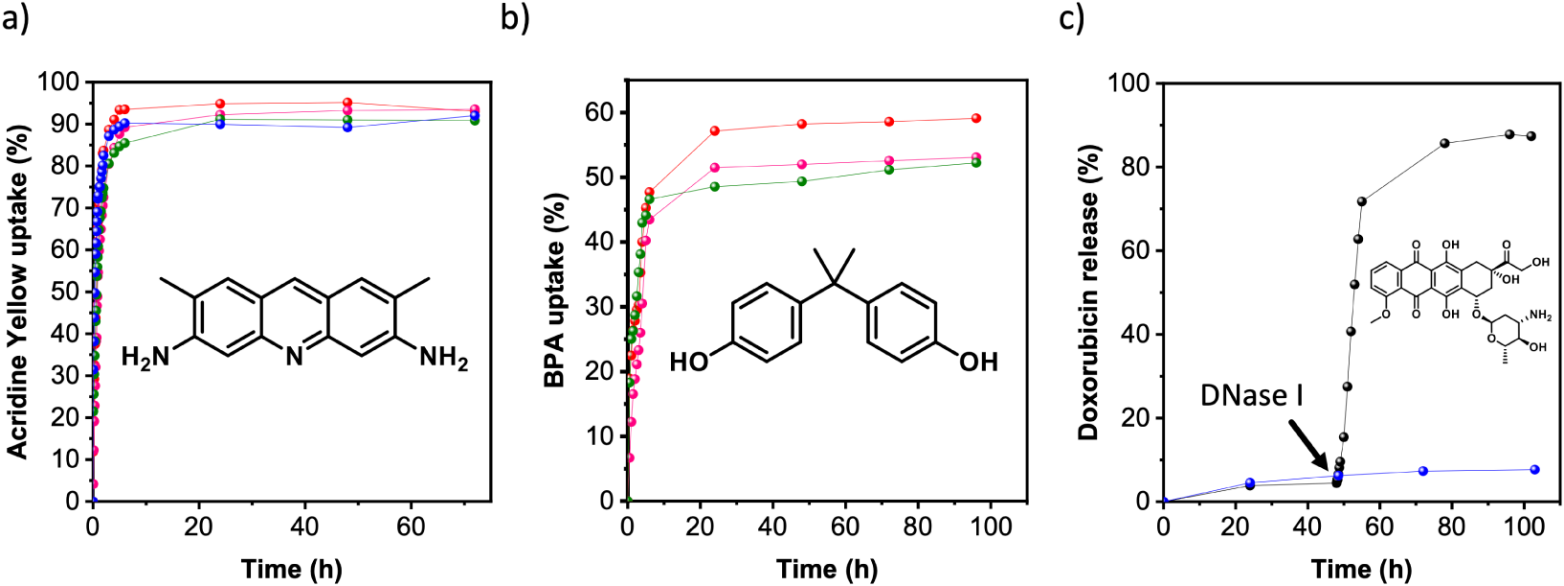
(a) Time-dependent removal of acridine yellow (initial concentration: 10 (red), 7 (pink), 6 (green) and 4 ppm (blue)) in the presence of BCN-11_42 in aqueous medium. (b) Time-dependent removal of BPA (initial concentration: 200 (green), 80 (pink), and 40 ppm (red)) in the presence of BCN-11_42. (c) Release profile of doxorubicin encapsulated in BCN-11_13 (black) and BCN-11_42 (blue) in the absence and presence of DNase I, added 48 h after the start of the experiment.

We hypothesize that the MOP nodes’ control over the DNA network could be leveraged to tailor DNA hydrogels for specific applications, such as pollutant removal from water, which requires hydrogels with adsorptive sites, good mechanical properties, and high stability.^49,50^ To test this hypothesis, we employed the dense, mechanically robust hydrogels derived from BCN-11_42 as adsorbents for Acridine Yellow, as a model of a DNA-intercalating pollutant.^51^ Time-dependent experiments showed that BCN-11_42 hydrogel adsorbed over 90% of pollutant at initial concentrations in the range of 4–10 ppm after 24 h (Figures 4a and S49–54). This adsorption process is best described by the Freundlich model, which implies a multilayer adsorption mechanism and heterogeneous distribution of adsorptive sites (Figure S40 and Table S4). The obtained correction factor was found to be less than 1 (*n* = 0.68), which is characteristic of a favorable process. Time-dependent removal data were fitted against the pseudo-second-order kinetic model with good correlation (R^2^ > 0.97), indicating strong chemical interaction between the absorbent and adsorbate molecules, which we ascribed to the high density of DNA adsorptive sites in the network (Figures S41–44). This hypothesis was further supported by the fitting of the kinetic data to the Elovich model, which showed a good correlation (R^2^ > 0.87). The strong agreement with the Elovich model indicates that the adsorption process involves significant chemical interactions (chemisorption) on heterogeneous surfaces (Figures S45–S48).

Once we confirmed that DNA in the BCN-11_42 network is an efficient adsorptive site for planar, intercalating molecules, we next studied the potential of the MOP nodes as adsorptive sites. To this end, we selected the Bisphenol A (BPA), a widely used industrial chemical of concern due to its severe toxicological and adverse health effects that cannot be trapped by DNA.^52,53^ We begun by studying the interaction of BPA with ONa-RhMOP in solution through NMR spectroscopy. The ^1^H NMR spectrum of a solution containing ONa-RhMOP and 6 mol. eq. of BPA revealed that the aromatic and aliphatic signals of BPA were shifted upfield, indicative of shielding (Figure S55). Additionally, the NMR signals attributed to BPA appeared broadened compared to the free molecule. Furthermore, the DOSY-NMR spectra showed that the diffusion coefficient of BPA decreased from 5.31·10^–10^ m^2^/s to 2.6·10^–10^ m^2^/s upon the addition of ONa-RhMOP (Figure S56). These results indicate that BPA interacts with the MOP through a slow exchange rate, operating on the NMR time scale, via supramolecular forces on the surface of the MOP.^54^

After confirming the interaction between the MOP and BPA in solution, we evaluated the performance of ONa-RhMOP as adsorptive sites in the DNA-MOP network. To this end, we first performed time-dependent removal experiments at different concentrations (40, 80, and 200 ppm) of BPA with BCN-11_42 hydrogel (Figures S57–S62). These experiments revealed BPA uptakes of 48% (200 ppm), 51% (80 ppm), and 57% (40 ppm) after 24 h of incubation (Figure 4b). In each case, the kinetics of the capture process followed the pseudo-second-order model with good correlation (R^2^ > 0.95; Figures S63–S65) and also fit well to the Elovich model (Figures S66–S68). These results signify that the BPA molecules exhibit strong interactions with the adsorptive sites. Moreover, we found that the adsorption isotherm of this BPA removal study is well fitted with the Freundlich adsorption isotherm model with good correlation (R^2^ = 0.99) (Figure S69). This result indicates a multilayer adsorption mechanism and heterogeneous distribution of adsorptive sites, similar to the previous pollutant. Therefore, these experiments confirm that the MOP cavities are accessible in the swollen BCN-11_42 hydrogel network, thereby designating these hydrogels as dual-site adsorbents, with performance comparable to that of state-of-the-art materials (Table S5).

### Enzyme-Triggered Release from BCN-11 Derived Hydrogels

Once the guest uptake was confirmed for BCN-11 derived hydrogels, we aimed to demonstrate enzyme-triggered release of active compounds, taking doxorubicin as a model molecule. Thus, the DNase I-degradable BCN-11_13 hydrogel was loaded with doxorubicin (drug loading of 105 mg/g) and incubated in water in the presence of the nuclease. This experiment revealed that the passive release of doxorubicin from the hydrogel in the absence of enzyme was less than 10%, which we attributed to the strong interaction between DNA and doxorubicin.^55^ However, after 24 h incubation of the drug-loaded hydrogel in a solution containing DNase I, 90% of the doxorubicin had been released. In contrast, for drug-loaded hydrogels derived from BCN-11_42 (drug loading of 24 mg/g) and studied under identical conditions, only 8% of the doxorubicin had been released (Figures 4c and S74).

## Conclusions

In conclusion, we have demonstrated the use of MOPs as molecular nodes for synthesizing highly connected DNA networks, which can be processed into mechanically robust aerogels and hydrogels capable of capturing and storing molecules from air and water, respectively. These findings not only pave the way for integrating molecular cage chemistry^56,57^ with the (bio)chemical properties of DNA but also provide synthetic guidelines for upcycling biomass-derived DNA into functional materials.

## Supporting Information Available

Detailed synthesis and methods, UV–vis spectra, FT-IR spectra, ^1^H NMR spectra, DOSY-NMR spectra, composition analysis, FE-SEM images, TGA analysis, rheology measurements, uniaxial compression tests, and gas adsorption–desorption isotherms (PDF)
