# Supplementary material for: DNA-Based Networks Formed by Coordination Cross-Linking of DNA with Metal–Organic Polyhedra: From Gels to Aerogels to Hydrogels
Source: J Am Chem Soc. 2025 May 1;147(19):16560–7. doi: 10.1021/jacs.5c03934 (PMC12082623; doi:10.1021/jacs.5c03934)
Supplement: Supplementary file 1 — ja5c03934_si_001.pdf [file ja5c03934_si_001.pdf]

DNA-Based Networks Formed by Coordination Cross-Linking of DNA with Metal-Organic Polyhedra: from Gels to Aerogels to Hydrogels.

Laura Hernández-López,[a,b] Akim Khobotov-Bakishev,[a,b] Alba Cortés-Martínez,[a,b] Eduard Garrido Ribó,[a,b] Partha Samanta,[a,b] Sergio Royuela,[c] Félix Zamora,[c] Daniel Maspoch,[a,b,d*] Arnau Carné-Sáncheza,[b*]

[a.] Catalan Institute of Nanoscience and Nanotechnology (ICN2), CSIC, and The Barcelona Institute of Science and Technology, Campus UAB, 08193 Bellaterra, Spain

[b.] Departament de Química, Facultat de Ciències, Universitat Autònoma de Barcelona, 08193 Bellaterra, Spain.4

[c.] Universidad Autónoma de Madrid, Departamento de Química Inorgánica, Ciudad Universitaria de Cantoblanco, Madrid, 28049 Spain.

[d.] ICREA, Pg. Lluís Companys 23, 08010 Barcelona, Spain

Table of contents:

S1. Materials and methods:

S1.1 Materials and characterization……………………………………………………S3

S1.2. Synthetic procedures…………………………………………………………..S5

S2. Characterization ……………..……………………………………………………………S11

S3. References………………………………………………………………………….S90

S1. Materials and methods

S1.1 Materials and characterization

Rhodium acetate was purchased from Across Organics. Sodium carbonate (Na_2CO_3), 5-hydroxy isophthalic acid (OH-bdc), sodium hydroxide, acridine yellow, doxorubicin hydrochloride and deoxyribonucleic acid sodium salt from salmon (*Orcerhynchus keta*) testes were purchased from Sigma-Aldrich. The whole genome of DNA employed can be found in the literature.[1] Solvents at HPLC grade were purchased from Fischer Chemicals. All reagents and solvents were used without further purification. Deionized water was obtained with a Milli-Q® system (18.2 MΩ cm).

Ultraviolet-visible (UV−vis) spectra were measured using a Thermo Scientific™ NanoDrop 2000 at room temperature (*ca.* 25 °C).

Solid-state ultraviolet-visible (UV−vis) spectra were measured using a Cary 4000 UV-Vis spectrometer (Agilent Technologies, Santa Clara, USA) with a UV-Vis Internal Diffuse Reflectance setup (model DRA-900). The DNA-based hydrogels samples were measured directly, whereas solvogels and aerogels were fragmented into small particles to ensure a proper measurement. Barium sulfate was used as a blank.

Rheology measurements of the gels were made using a stress-controlled Haake Rheo Stress 1 (Thermo Electron Corporation, Karlsruhe, Germany) rheometer. The rheological measurements of all materials were conducted through frequency sweeping in shear mode with a 1 % deformation amplitude, which was well inside the linear regime. The measurements were performed with the as-made hydrogels, except the solvogels, which were first exchanged with isopropanol for 24 h, to avoid eventual solvent loss during the measurements.

Field-emission scanning electron microscopy (FESEM) was performed in an FEI Quanta 650FEG ESEM and an FEI Magellan 400L XHR SEM.

Fourier-Transform Infrared Spectroscopy (FTIR-ATR) measurements were performed using a Bruker Alpha II spectrophotometer with a diamond reflection ATR module.

Volumetric N_2 isotherms were collected at 77 K using a High-Resolution ASAP 2460 (Micromeritics). The temperature for N_2 isotherms measurement was controlled by using a liquid nitrogen bath. BET surface values were calculated according to the BETSI method.[2]

Volumetric CO_2 isotherms were collected at 298 K using ASAP 2460.

Supercritical CO_2 drying was performed using a Laboratory Supercritical Fluid Equipment SFE 15 mL (Extratex Supercritical Fluid Innovation, France).

Uniaxial compression tests were carried out on an Instron 3400 Series Universal Testing Machine with a 500 N load cell. Samples were compressed to a maximum strain of 80% at a rate of 1.0 mm min^{-1}, with a preload of 0.1 N.

Inductively Coupled Plasma Optical Emission spectroscopy (ICP-OES) (ICP-OES) analyses were performed in the Chemical Analysis Service at the UAB with an Agilent equipment (model 5900). For the solid samples, 10 mg of sample were digested with 4 mL of concentrated ultrapure nitric acid (HNO_3 70%) in an analytical microwave Anton Paar Multiwave 7000 at 250 °C. Subsequently, the digestion residues obtained and the liquid samples were suitably diluted with a nitric acid solution (HNO_3 2%) to analyze the elements of interest by ICP-OES. The quantification was performed by interpolation on a calibration curve prepared from commercial standards of the elements of interest. The assays were performed in duplicates.

Nuclear magnetic resonance (NMR). All ^{1}H NMR spectra were recorded using a Bruker Avance NEO 300 NMR or AVANCE 500 NMR spectrometer operating at 500.13 MHz and equipped with a cryoprobe zgradient inverse TCI probehead spectrometer at 25 °C. Chemical shifts (δ) are reported in ppm.

S1.2. Synthetic procedures

Synthesis of OH-RhMOP. OH-RhMOP was synthesized according to a previously described methodology.[3]

UV-Vis Titration experiments with nucleosides and nucleotides in water. Titration experiments were performed in water at a pH=11 and using a ONa-RhMOP concentration of 2 mg/ml. The same concentration was used when the titration was performed in methanol.

UV-Vis Titration experiments with nucleosides and nucleotides in water after being exposed to methanol: ONa-RhMOP (2.0 mg, 0.29 μmol) was dissolved in basic water (1 mL, pH = 11), and the resulting green solution was lyophilized to obtain a green powder. The lyophilized powder was dissolved in methanol (1 mL) and adenosine monophosphate (6 mol. eq.) was added to the solution. The methanol solution containing ONa-RhMOP and adenosine monophosphate was dried and the solid was re-dissolved in water (2 mL).

^{1}H-NMR characterization of the interaction of ONa-RhMOP with nucleotides in D_2O after being exposed to methanol. ONa-RhMOP (4.0 mg, 0.58 μmol) was dissolved in basic water (1 mL, pH = 11), and the resulting green solution was lyophilized to obtain a green powder. The lyophilized powder was dissolved in methanol (1 mL) and the desired nucleotide (6 mol. eq.) was added. The methanol solution containing the ONa-RhMOP and the corresponding nucleotide was evaporated and the solid was re-dissolved in D_2O (0.5 mL).

Synthesis of BCN-11 aerogels. BCN-11_42 was prepared as follows. 30 mg of deoxyribonucleic acid sodium salt from salmon testes were dissolved in 0.33 ml of NaOH (0.20 M) aqueous solution, resulting in a transparent colorless dense liquid. Separately, 30 mg (4.4 μmol) of OH-RhMOP were dissolved in 0.54 mL of aqueous solution containing 24 molar equivalents (in respect to the MOP) of NaOH (105 μmol). The MOP solution was added on top of the DNA precursor solution under vigorous stirring. The homogenous mixture gelified instantaneously leading to a transparent green gel. The gel was transferred to a syringe, sealed with parafilm, and kept at room temperature for 36

h. The obtained green gel was carefully removed from the syringe to undergo a solvent exchange process. The solvent exchange process entailed the washing of the gel with methanol (*ca.* 20 mL every day) for 5 days, to yield a dark purple solvogel. The methanol-exchanged solvogel was first exchanged with liquid CO_2 at 25°C and 60 bar for 30 min. Next, the liquid CO_2 was transformed into supercritical CO_2 ($scCO_2$) by increasing the pressure to 140 bar at 40°C. The sample was kept under $scCO_2$ for 2 h. Finally, the CO_2 was slowly vented for 1 h to reach 1 bar. After this process, a dark purple DNA-based aerogel was obtained (BCN-11).

BCN-11_13, BCN-11_21 and BCN-11_35 were prepared following the above detailed procedure, being the only difference the concentration of OH-RhMOP and NaOH in the initial solution. Thus, the initial 0.54 mL of the aqueous MOP solution for the synthesis of BCN-11_13, BCN-11_21 and BCN-11_35 contained 5 mg (0.73 µmol), 10 mg (1.46 µmol) and 20 mg (2.93 µmol) of MOP, respectively. In each of these solutions, 24 molar equivalents of NaOH were added prior mixing with the DNA solution.

Hydrogel formation from BCN-11 aerogels: Hydration process of all BCN-11 aerogels was carried out following the same procedure. BCN-11 aerogels were incubated in 150 mL of MiliQ water for 24 h. After 24 h incubation, purple hydrogels with different swelling ratio depending on their composition were obtained.

Swelling calculation of the DNA-based hydrogel: Swelling tests were conducted using a gravimetric method. BCN-11 aerogels with known weights were immersed in Milli-Q water. After 24 h of incubation, the hydrated gels were removed, wiped with filter paper to eliminate excess water from the surface, and then weighed. The swelling ratio (water uptake) (S_{ratio}) was calculated according to the following equation:

$$S_{ratio} = \frac{M_H - M_A}{M_A}$$

where M_H is the mass of the swollen hydrogel in water and M_A is the mass of the original aerogel.

Stability of hydrogels in DNase I: Stability towards the presence of DNase I was tested using BCN-11 derived hydrogels prepared as detailed above. BCN-11 hydrogels were incubated in 40 mL of digestion solution that contained a 10% v/v reaction buffer required to keep DNase I active and 2% v/v of a DNase I solution (2 U/µL). The reaction buffer was prepared using tris(hydroxymethyl)aminomethane (TRIS) (100 mM), $CaCl_2$ (5 mM) and $MgCl_2 \cdot 6\ H_2O$ (25 mM) in water. After 24 h of incubation of the hydrogels in the digestion solution, the remaining supernatants were analyzed by ICP-MS. In addition, blank experiments were run to ensure that the observed degradation is ascribed to the enzymatic degradation. For these blank experiments, BCN-11 hydrogels were incubated for 24 h in 40 mL of the digestion media (10% v/v reaction buffer) without the presence of DNase I. The remaining supernatants were also analyzed by ICP-MS.

Acridine yellow adsorption experiments: Concentration (C_t in ppm) of acridine yellow at time t was calculated by using the following equation:

$$C_t = C_0 - \left(\frac{A_0 - A_t}{A_0}\right) \times C_0$$

where A_0 and C_0 are the initial absorbance and concentration (in ppm) of acridine yellow solution, respectively. And A_t is the absorbance of the same solution at a specific time t (in min).

Capacity of the adsorbents at the equilibrium (Q_e) was calculated as follows:

$$Q_e = \frac{(C_0 - C_t) \times V}{m}$$

where *V* (in L) and m (in g) are the volume of the acridine yellow solution and the mass of the adsorbent used for the study.

Acridine yellow adsorption isotherm experiment: In each case, the BCN-11_43 aerogel (15 mg) was rinsed with a small amount of water multiple times (usually three times) until the washing water had a neutral pH. After that, the gel was incubated in 10 mL of water and was allowed to swell for 24 h.

A stock solution of 10 ppm was prepared by dissolving Acridine Yellow (10 mg, 0.04 mmol) in 1 L of water. From this solution, a set of dilutions were prepared to obtain 10 mL solutions of Acridine Yellow at 4, 6, and 7 ppm. A calibration curve was made from these solutions. The swollen gels were immersed in 10 mL solution of Acridine Yellow in water (at pH= 7) at different concentrations (4 ppm, 6 ppm, 7 ppm and 10 ppm). UV-vis was measured periodically (every 5 min for the first hour; every 10 min for the second hour; and once every hour until the sixth hour) to monitor the uptake of Acridine Yellow by the hydrogel. Then, the capacity (Qe, in mg·g^{-1}) and concentration (Ce, in ppm) at equilibrium produced a non-linear curve, which was further fitted with the following equations:

Langmuir adsorption isotherm model, $Q_e = \frac{Q_m K_L C_e}{1+K_L C_e}$

Freundlich adsorption isotherm model, $Q_e = K_F C_e^{1/n}$

where Q_e (mg g^{-1}) is the maximum amount of Acridine Yellow per unit mass of adsorbent to form a complete monolayer, and K_L (L·mg^{-1}) is the Langmuir constant.

K_F and 1/n are the Freundlich model constants, which indicate the capacity and favorability of adsorption, respectively.

Acridine yellow adsorption kinetic experiment: For the kinetic experiment with the adsorbent material and Acridine Yellow solution (at a specific concentration), the adsorption process was monitored at different time intervals as described above by UV-Vis spectroscopy. Then, the experimentally obtained data was fitted in the following pseudo second order kinetic equation and Elovich model:

Pseudo second order kinetic model, $Q_t = \frac{k_2 Q_e^2 t}{1+k_2 Q_e t}$

Elovich model, $Q_t = \frac{1}{\beta} \ln{(1 + \alpha\beta t)}$

where Q_t and Q_e are the amounts of pollutants adsorbed on the adsorbent (mg·g^{-1}) at different time intervals (t) and at equilibrium, respectively. Moreover, α represents the initial adsorption rate, whereas, β represents the extent of surface coverage and activation energy in chemisorption.

Bisphenol A adsorption experiments. BCN-11_42 aerogel (*ca.* 15 mg) was rinsed with 25 mL of water multiple times (usually three times) until washing water had a neutral pH. After that, the gel was incubated in 10 mL of water and was allowed to swell for 24 h. The swollen gel was incubated in 10 mL solution of BPA at different concentrations (40 ppm, 80 ppm and 200 ppm). To monitor the uptake of BPA, an aliquot of 0.1 mL was taken periodically and added to deuterated water containing fumaric acid as internal standard. The amount of internal standard for the experiments performed at 40 ppm, 80 ppm and 200 ppm of BPA was 0.002, 0.005 and 0.007 mg, respectively. The uptake of pollutant per amount of adsorbent, initial pollutant concentration and time was fitted to isotherm and kinetic models, as explained above.

Calibration curve of doxorubicin in digestion media with and without DNase I. A 2.5 mg/mL stock solution was prepared by dissolving 25 mg of doxorubicin (25 mg, 0.046 mmol) in 10 mL of water. This stock solution was diluted in the digestion media without DNase I. A calibration curve was plotted taking 480 nm as λ_{max}. To ensure there were no deviations in the λ_{max} in the presence of DNase I, a second calibration curve was prepared in the presence of DNase I.

DNase I triggered release of doxorubicin. BCN-11_13 (30 mg) and BCN-11_45 (54 mg) were swollen in the digestion media in the absence of DNase I prior to doxorubicin loading process. To do so, BCN-11-13 and BCN-11-45 were immersed in 50 mL of digestion media (in absence of DNase I) for 24 h.

In parallel, 50 ml of doxorubicin solution at a concentration of 0.2 mg/mL was prepared using digestion media (without DNase I) as solvent. A swollen gel was introduced to this

solution and allowed to stand for 24 h covered and shielded from light. A dark red gel was obtained from this process, which was rinsed three times with 10 ml of the digestion solution. The washes were combined with the uptake solution and the final volume was brought to 100 mL in a volumetric flask. The amount of doxorubicin absorbed by the gel was calculated by comparing the initial and the final concentration of doxorubicin in solution. The doxorubicin uptakes for BCN-11_13 and BCN-11_45 were 105 mg/g and 24 mg/g, respectively.

The gel loaded with doxorubicin was then introduced in 40 mL of digestion media without DNase I. Over the period of 48 h, the passive release of doxorubicin was tracked using with UV-vis spectroscopy. After 48 h, the gel was transferred into a 40 mL of digestion solution containing 2% v/v DNase I. The release of doxorubicin caused by the enzymatic activity was followed by UV-Vis spectroscopy. To ensure proper quantification of doxorubicin with and without DNase I, calibration curves were prepared of doxorubicin in digestion media with and without DNase I.

Cell culture and cell viability assay. To test the *in vitro* cytotoxicity of the degradation products of BCN-11_13 after being exposed to DNase I, 1BR3G human skin fibroblasts cells were seeded in 96-well plates at a concentration of 1 x 104 cells/well and incubated for 24 h. After 24 h, the medium was changed to fresh medium containing the solution containing the DNase I degraded BCN-11_13 at different concentrations of degraded hydrogel (0.01 mg/ml to 0.05 mg/ml), and cells were left to incubate for 24 h. As control, cells were either exposed to DMSO (5% or 10%) or left untreated. Cell viability was then measured using the XTT method (Invitrogen CyQUANT XTT Cell Viability assay) according to the manufacturer´s instruction. Color change was quantified in a spectrophotometric plate reader (SpectraMax iD3). Cell viability was expressed as a percentage of the untreated control level. All experiments were performed in triplicates, in three independent experiments.

S2. Characterization

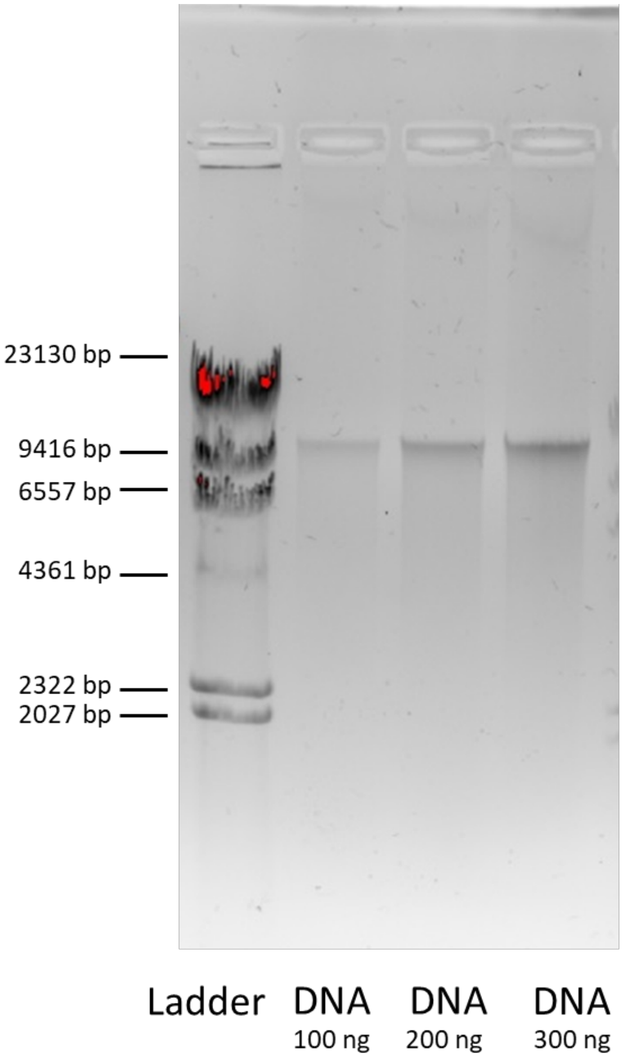

Figure S1. Gel electrophoresis (Agarose at 0.7%) characterization of salmon sperm DNA employed in this study.

.

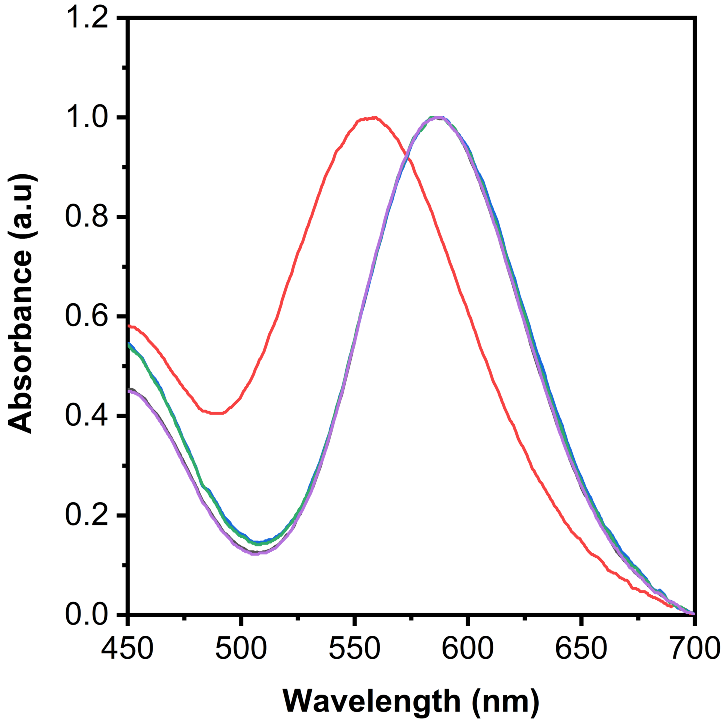

Figure S2. Normalized UV-Vis spectra of ONa-RhMOP in basic water (pH ~11) in the absence (grey) and the presence of 12 mol. eq. of adenosine (red), guanosine (blue), thymidine (green) and cytidine (purple). Note that the UV-Vis spectrum corresponding to the ONa-RhMOP and adenosine is the only one that shows a shift in λ_{max} (from 585 nm to 558 nm), indicating that adenosine is able to coordinate to the Rh(II) centers of the paddlewheel.

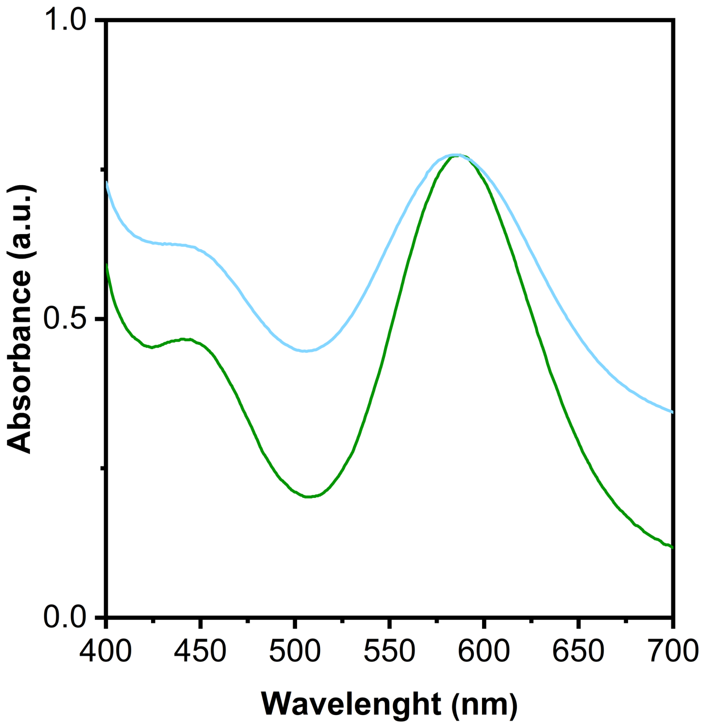

Figure S3. Solid-state UV-Vis absorption spectrum of DNA-MOP hydrogel (light blue), and UV-Vis absorption spectrum for ONa-RhMOP in aqueous solution. Note that the characteristic absorption band of the Rh-Rh bond centered at 586 nm (band I, λ_{max}) for the ONa-RhMOP remains constant after the formation of the DNA-MOP hydrogel, indicating the absence of coordinative interactions between the MOP and the DNA.

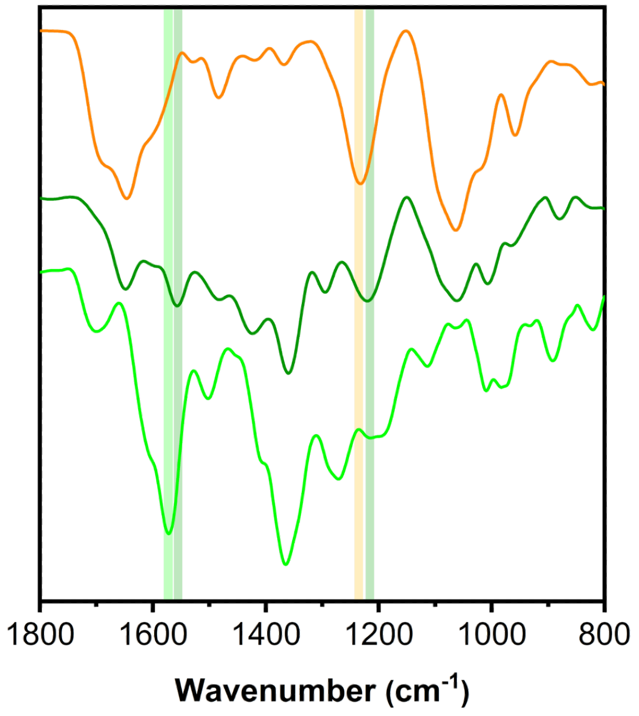

Figure S4. Magnified FT-IR spectra of DNA (orange), ONa-RhMOP (light green), and lyophilized DNA-MOP gel (dark green). Note that the peak at 1569 cm^{-1} corresponding to the carbonyl groups (C=O stretching band) of the ONa-RhMOP is shifted to 1555 cm^{-1}. Additionally, the peak associated with the asymmetric vibration of phosphate groups of the DNA shifts from 1231 cm^{-1} to 1219 cm^{-1}. These changes suggest that both functional groups are involved in H-bonding interactions.

a)

b)

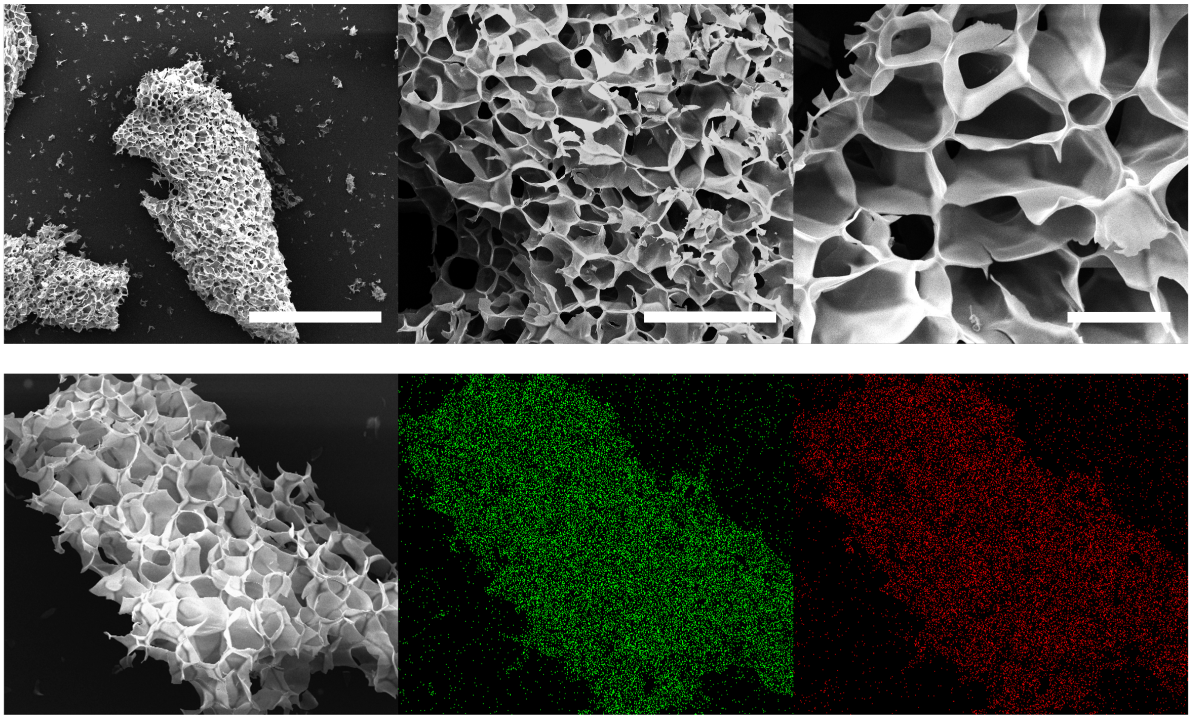

Figure S5. (a) FE-SEM images of the lyophilized DNA-MOP hydrogel at different magnifications. Scale bars, from left to right: 1 mm, 200 μm, and 50 μm. (b) EDX analysis of the lyophilized DNA-MOP hydrogel. The elemental maps indicate that rhodium (green bitmap) from ONa-RhMOP and phosphorus (red bitmap) from DNA are homogeneously dispersed throughout the sample.

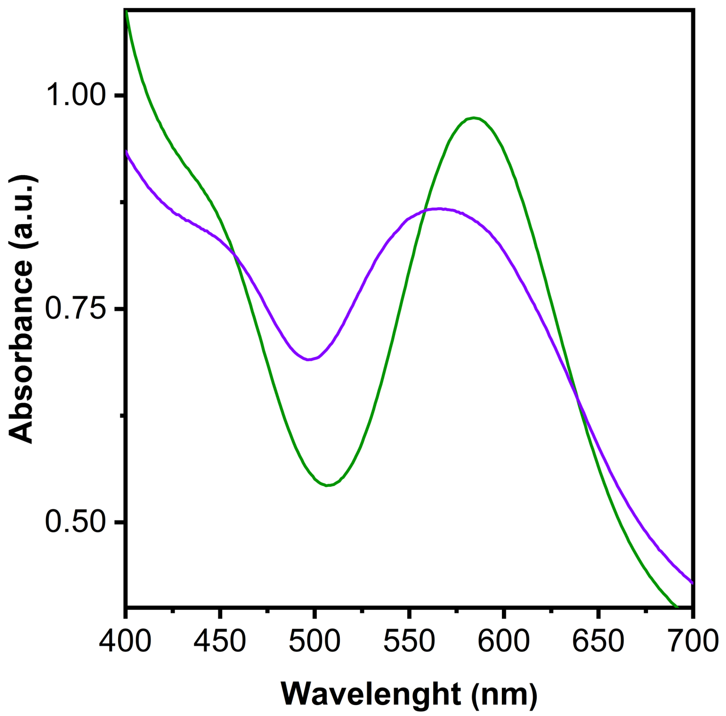

Figure S6. Solid-State UV-Vis absorption spectra for DNA-MOP hydrogel (green) and DNA-MOP solvogel (purple). Note that the characteristic absorption band of the Rh-Rh bond centered at 586 nm (band I, λ_{max}) for DNA-MOP hydrogel is shifted to 566 nm upon incubation in methanol, confirming the coordinative interaction between DNA and ONa-RhMOP.

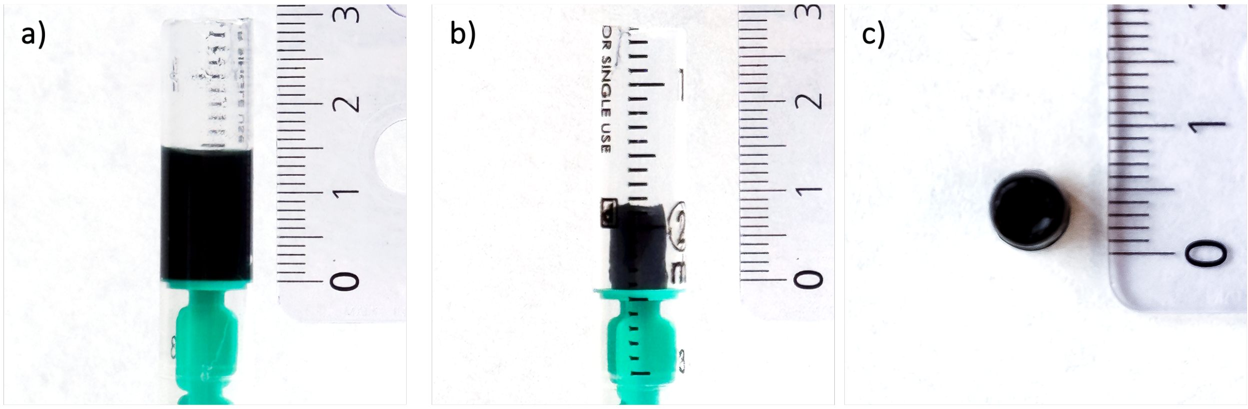

Figure S7. (a) Photograph of the DNA-MOP hydrogel before incubation in methanol. (b, c) Photographs of the DNA-MOP solvogel after the solvent exchange procedure in methanol.

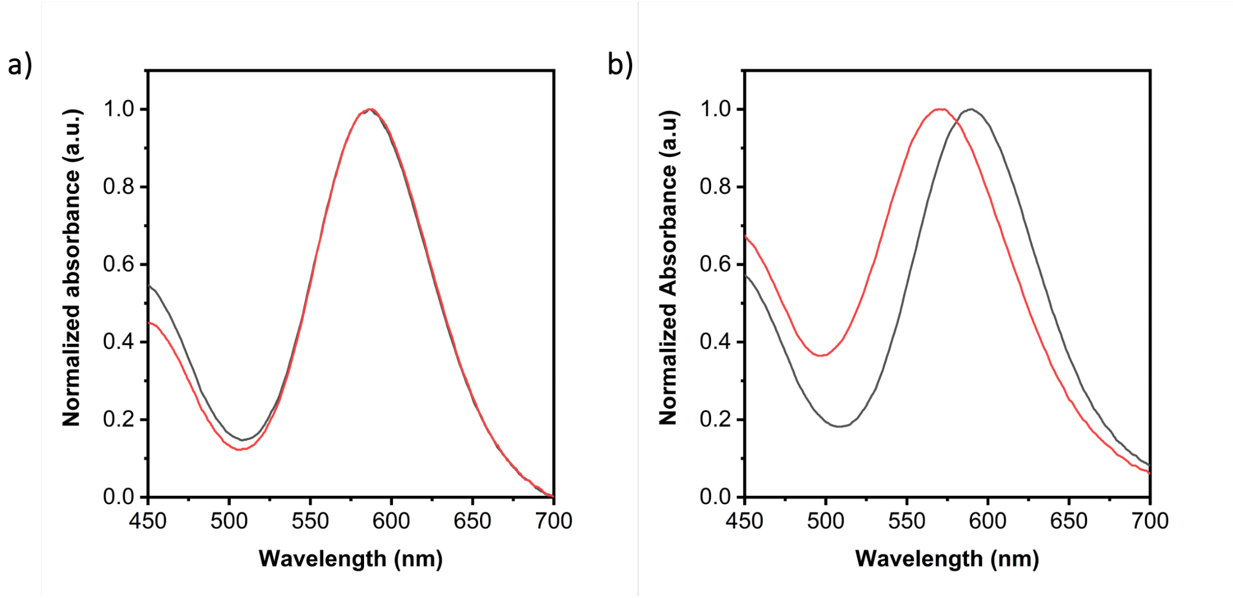

Figure S8. (a) Normalized UV-Vis spectra of ONa-RhMOP in basic water (pH ~11) in the absence (grey) and the presence of 12 mol. eq. of adenosine monophosphate (red). Note that the UV-Vis spectrum corresponding to the ONa-RhMOP and adenosine monophosphate does not show any shift in λ_{max}, thus confirming the absence of coordinative interaction. (b) Normalized UV-Vis spectra of ONa-RhMOP in methanol in the absence (grey) and the presence of 12 mol. eq. of adenosine monophosphate (red). Note that the UV-Vis spectrum corresponding to the ONa-RhMOP and adenosine monophosphate shows a shift in λ_{max}, from 590 nm to 570 nm, indicating that adenosine monophosphate is able to coordinate the Rh(II) centers of the paddlewheel in methanol.

Table S1. Composition of the BCN-11 aerogels.

Aerogel	Initial	Found				
**BCN-11_X**	MOP (% w/w)[a]	P (% w/w)[b]	DNA (% w/w)[c]	Rh (% w/w)[d]	MOP (% w/w)[e]	% w/w[a]
**BCN-11_13**	14	7.42	79.05	4.25	11.75	13
**BCN-11_21**	25	6.76	71.92	7.18	19.85	21
**BCN-11_35**	40	5.54	59.03	11.0	32.91	35
**BCN-11_42**	50	4.79	51.0	13.4	37.06	42

[a] defined as $(w_{ONa\text{-}RhMOP}/w_{(ONa\text{-}RhMOP+DNA)})$x100 This ratio does not account for eventual adsorbed water that can be removed through thermal activation.
[b] defined as (w_P/w_{sample})x100
[c] Calculated from the average molecular weight of nucleotide using the following formula: (Av. Mol. Weight nucleotide / mol. Weight P) x P % w/w.
[d] defined as (w_{Rh}/w_{sample})x100
[e] defined as as (w_{MOP}/w_{sample})x100 calculated from the molecular weight of [Rh24O-BDC24]: 6806 g/mol

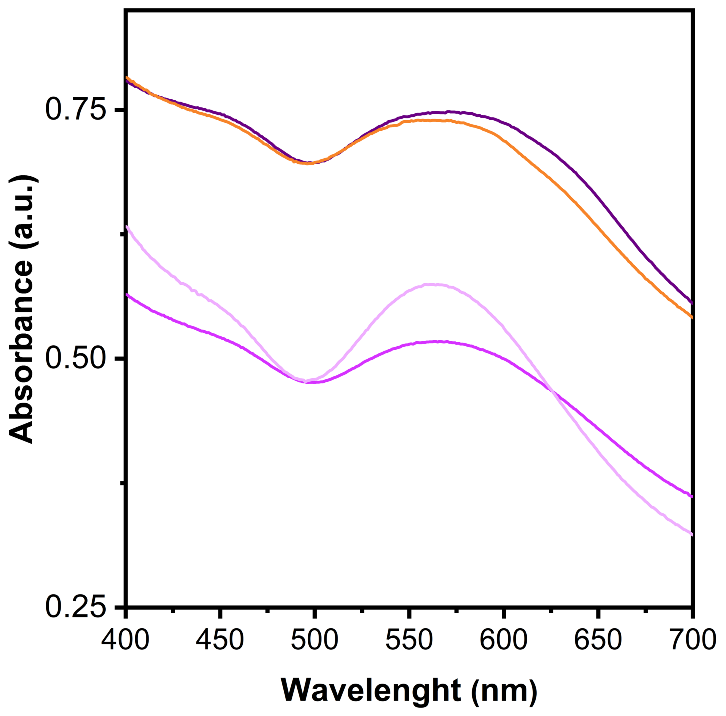

Figure S9. Solid-state UV-Vis absorption spectra for BCN-11_13 (light pink), BCN-11_21 (pink), BCN-11_35 (orange), and BCN-11_42 (purple). Note that the λ_{max} of the spectra of all BCN-11 aerogels are centered at *ca.* 570 nm, confirming the coordinative interaction between DNA and ONa-RhMOP.

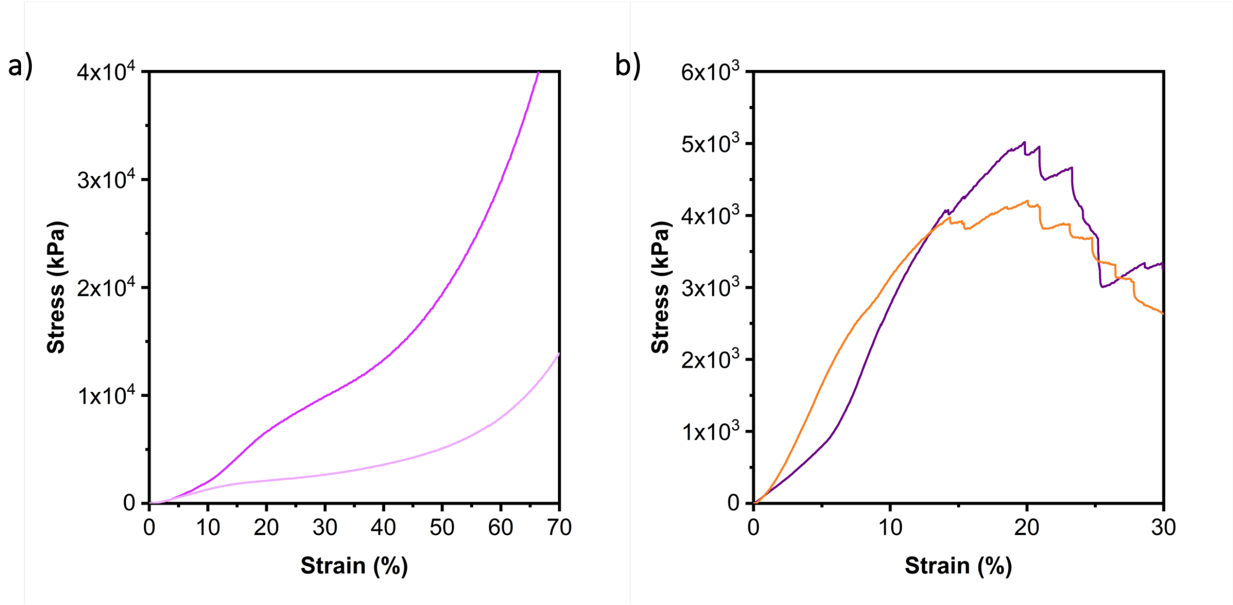

Figure S10. Uniaxial compression tests of BCN-11 aerogels. (a) Stress-strain curves of BCN-11_13 (light pink), and BCN-11_21 (pink), which behave as plastic materials and can be compressed into the densification region without failure. These plastic materials can be compressed to over 70% strain without fracture (b) Stress-strain curves of BCN-11_35 (orange) and BCN-11_42 (purple), which behave as brittle materials and fail by cracking under relatively small stress. Note that the initial segment of the curves is excluded from the analysis, as it is used to remove slack from the samples. Here "slack" refers to the initial looseness in the testing setup before the samples are in full contact with the compression plates. This is due to the difficulty in preparing shaped materials with perfectly flat and parallel surfaces for testing, which leads to small gaps, misalignments and incomplete contact at the beginning of the measurements.

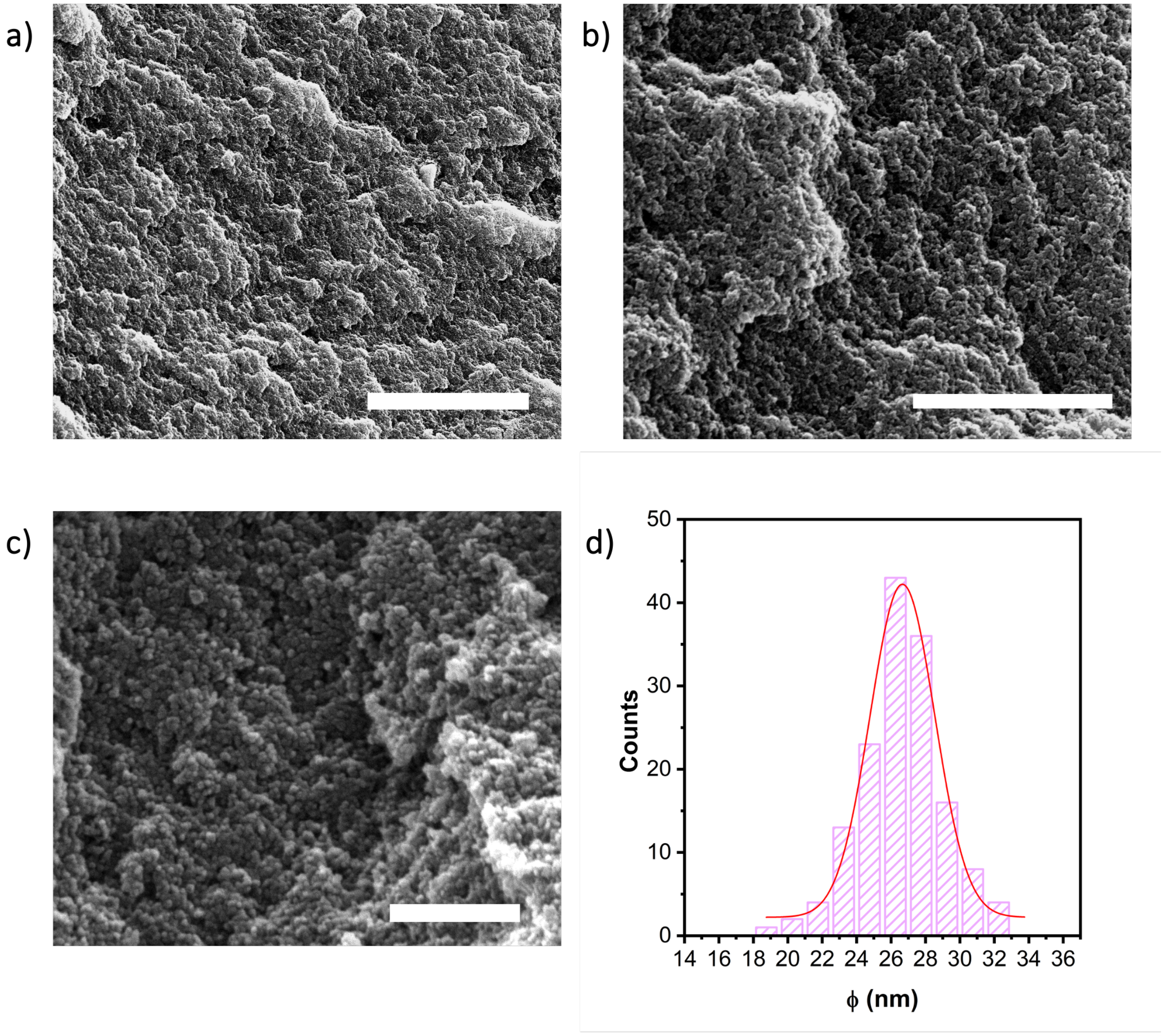

Figure S11. (a, b, c) FE-SEM images of the BCN-11_13 aerogel at different magnifications. (d) Particle size distribution histogram of the BCN-11_13 aerogel with a mean particle size of 27 ± 2 nm. Scale bars: 10 μm (a), 2 μm (b), and 500 nm (c).

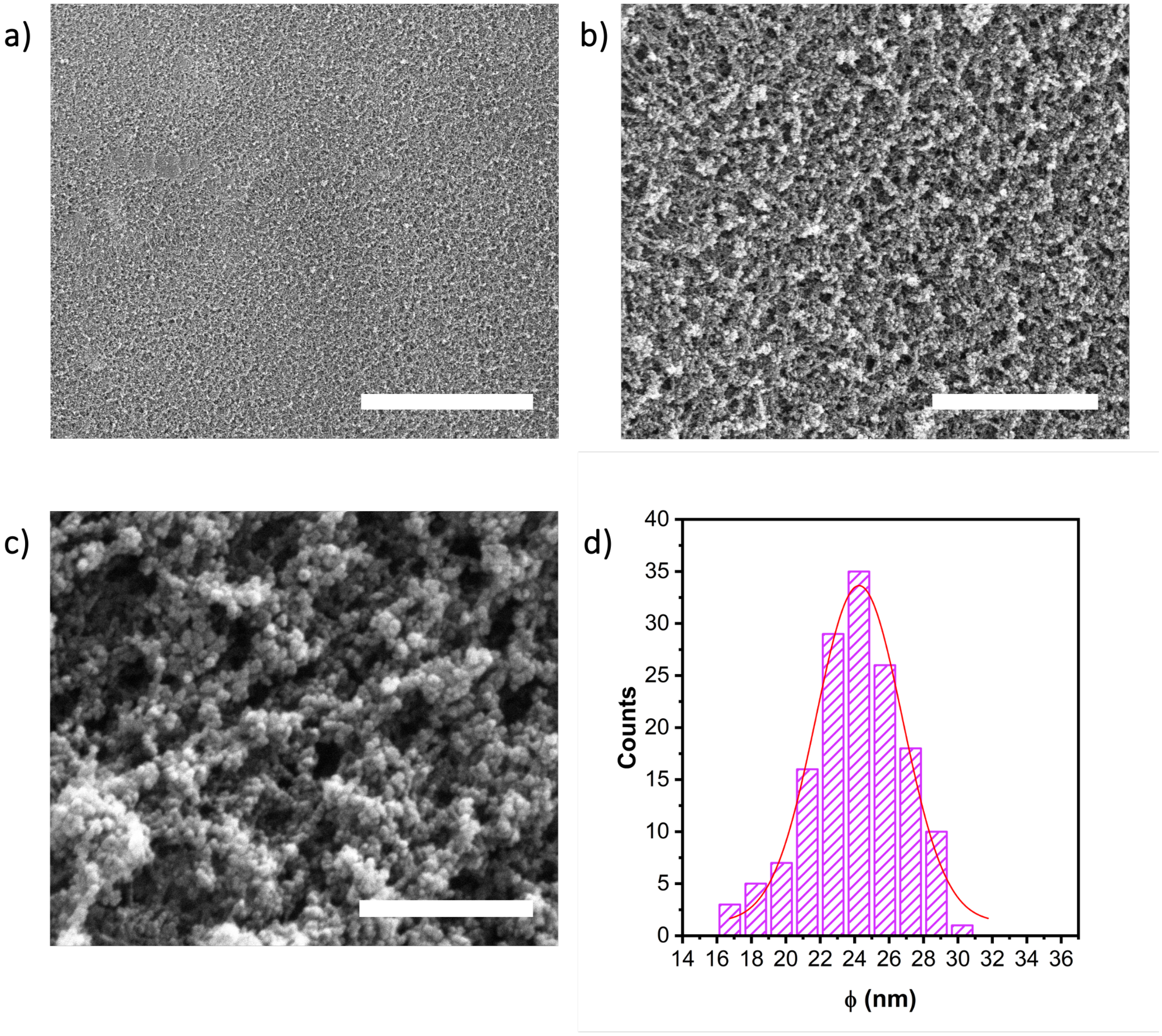

Figure S12. (a, b, c) FESEM images of the BCN-11_21 aerogel at different magnifications. (d) Particle size distribution histogram of the BCN-11_21 aerogel with a mean particle size of 24 ± 3 nm. Scale bars: 10 μm (a), 2 μm (b), and 500 nm (c).

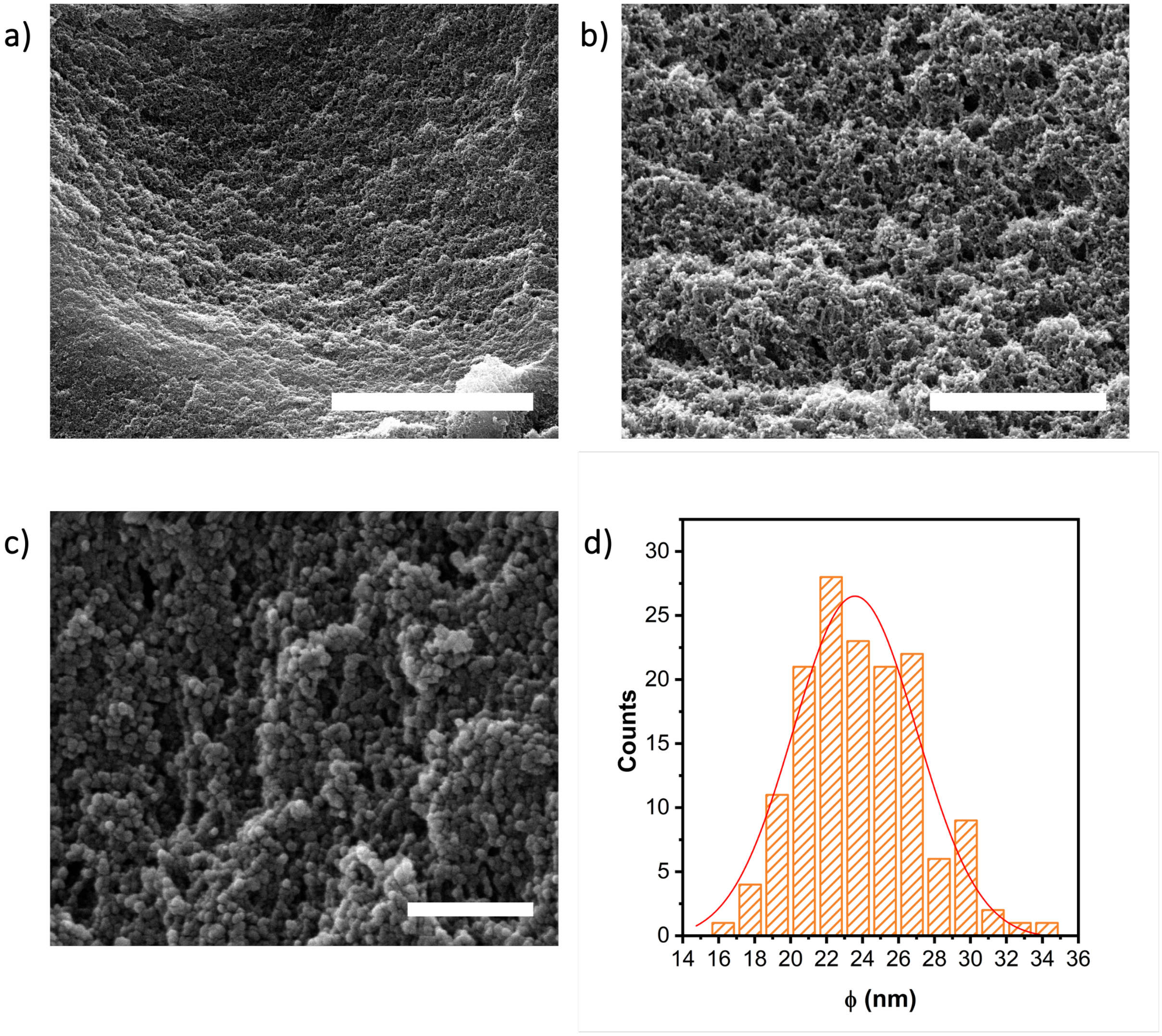

Figure S13. FESEM images of the BCN-11_35 aerogel at different magnifications. (d) Particle size distribution histogram of the BCN-11_35 aerogel with a mean particle size of 24 ± 4 nm. Scale bars: 10 μm (a), 2 μm (b), and 500 nm (c).

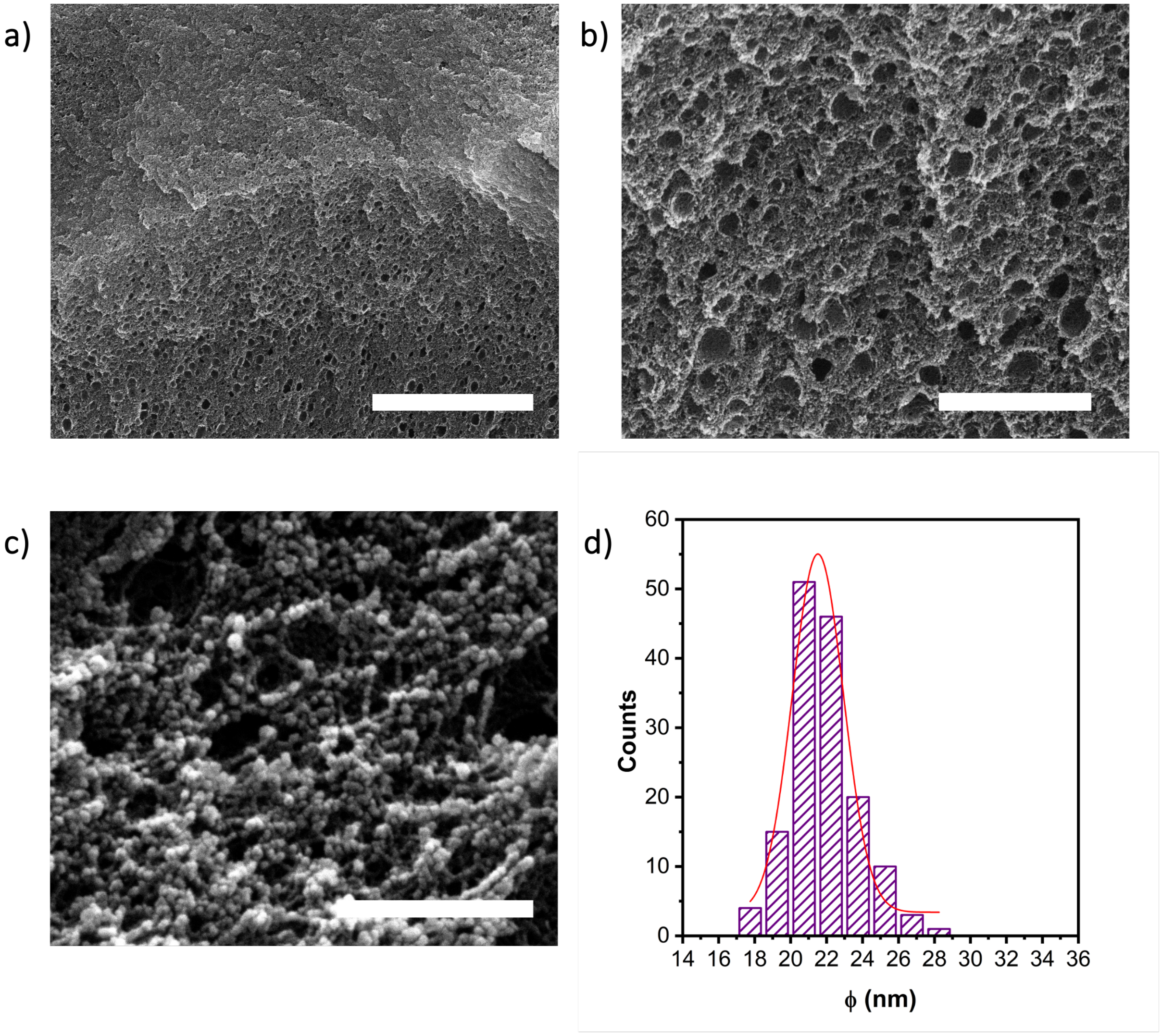

Figure S14. FESEM images of the BCN-11_42 aerogel at different magnifications. (d) Particle size distribution histogram of the BCN-11_42 aerogel with a mean particle size of 22 ± 2 nm. Scale bars: 10 μm (a), 2 μm (b), and 500 nm (c).

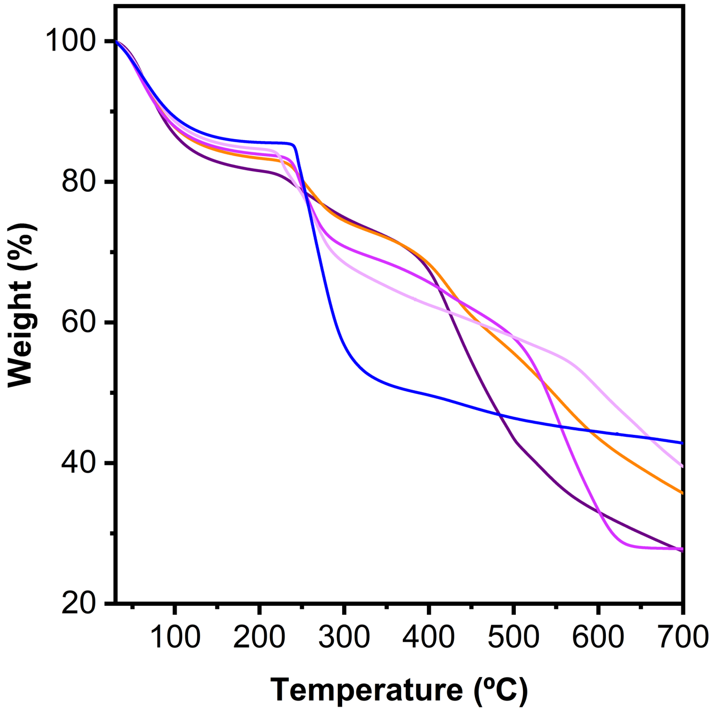

Figure S15. Thermogravimetric analysis of DNA (blue), BCN-11_13 (light pink), BCN-11_21 (pink), BCN-11_35 (orange) and BCN-11_42 (purple). Note that the initial mass loss of approximately 15% corresponds to the evaporation of residual water and solvent molecules in the samples. The thermogravimetric analysis reveals that all DNA-based aerogels undergo a two-step degradation process. The first degradation step occurs around 225 °C, similar to the degradation of pure DNA. The second degradation step varies with the mass percentage of the samples, with lower mass percentages resulting in higher degradation temperatures.

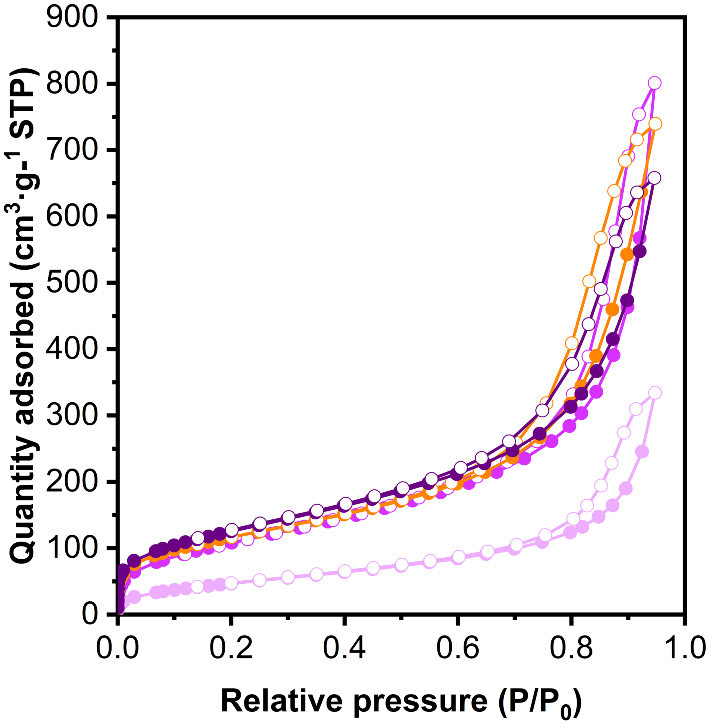

Figure S16. N_2-sorption isotherms for BCN-11_13 (light pink), BCN-11_21 (pink), BCN-11_35 (orange), and BCN-11_42 (purple).

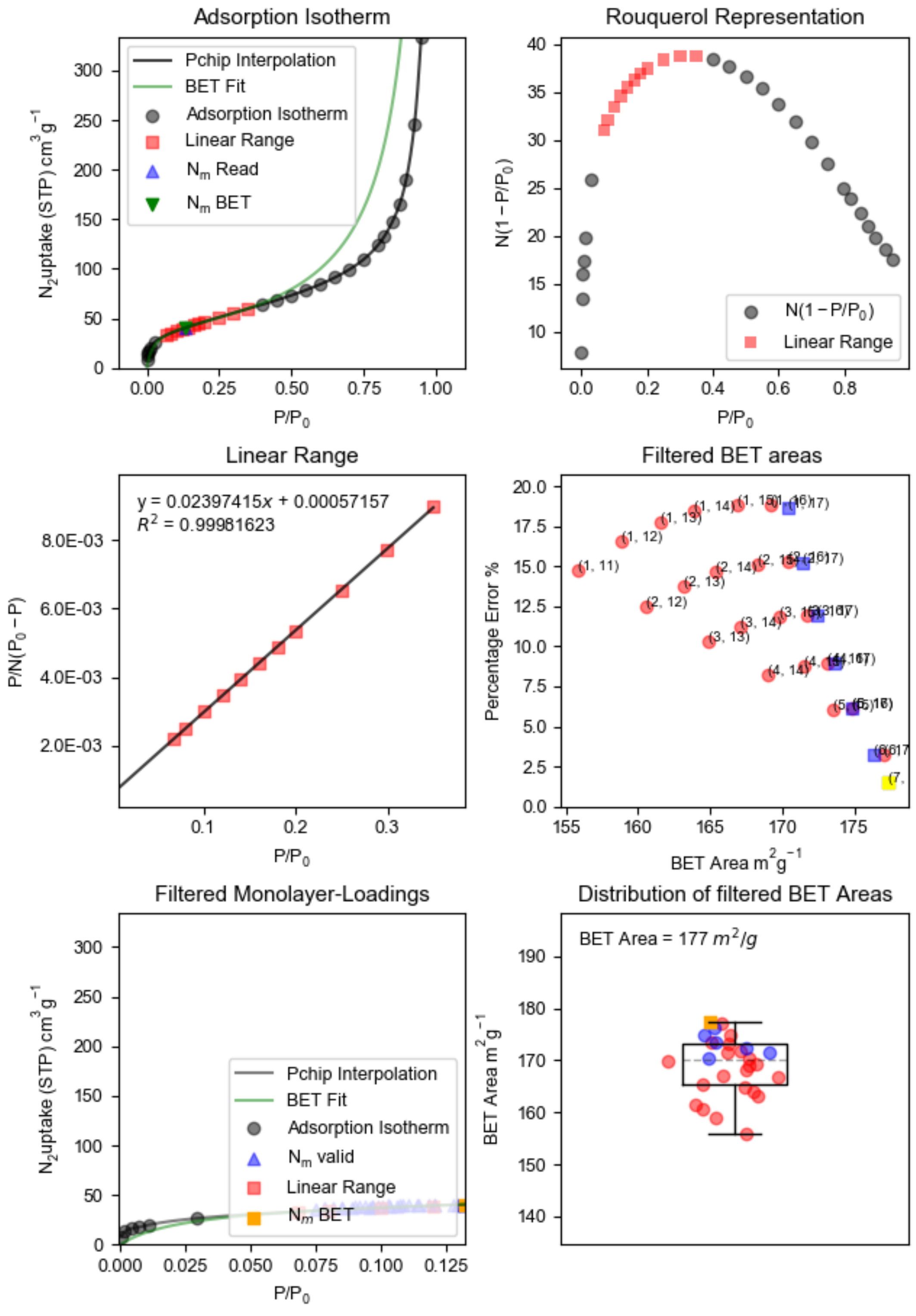

Figure S17. BETSI analysis for BCN-11_13.

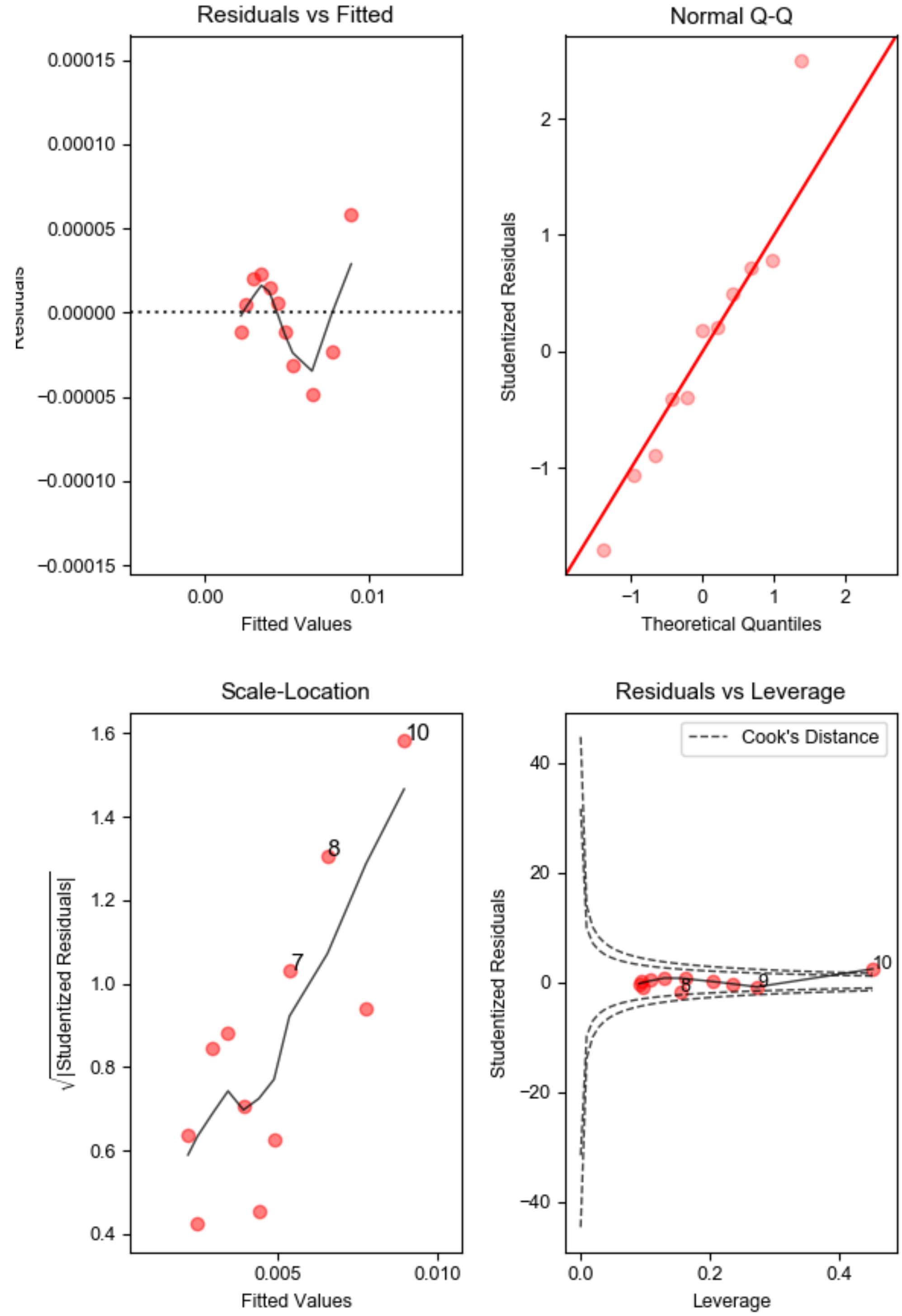

Figure S18. BETSI regression diagnostics for BCN-11_13.

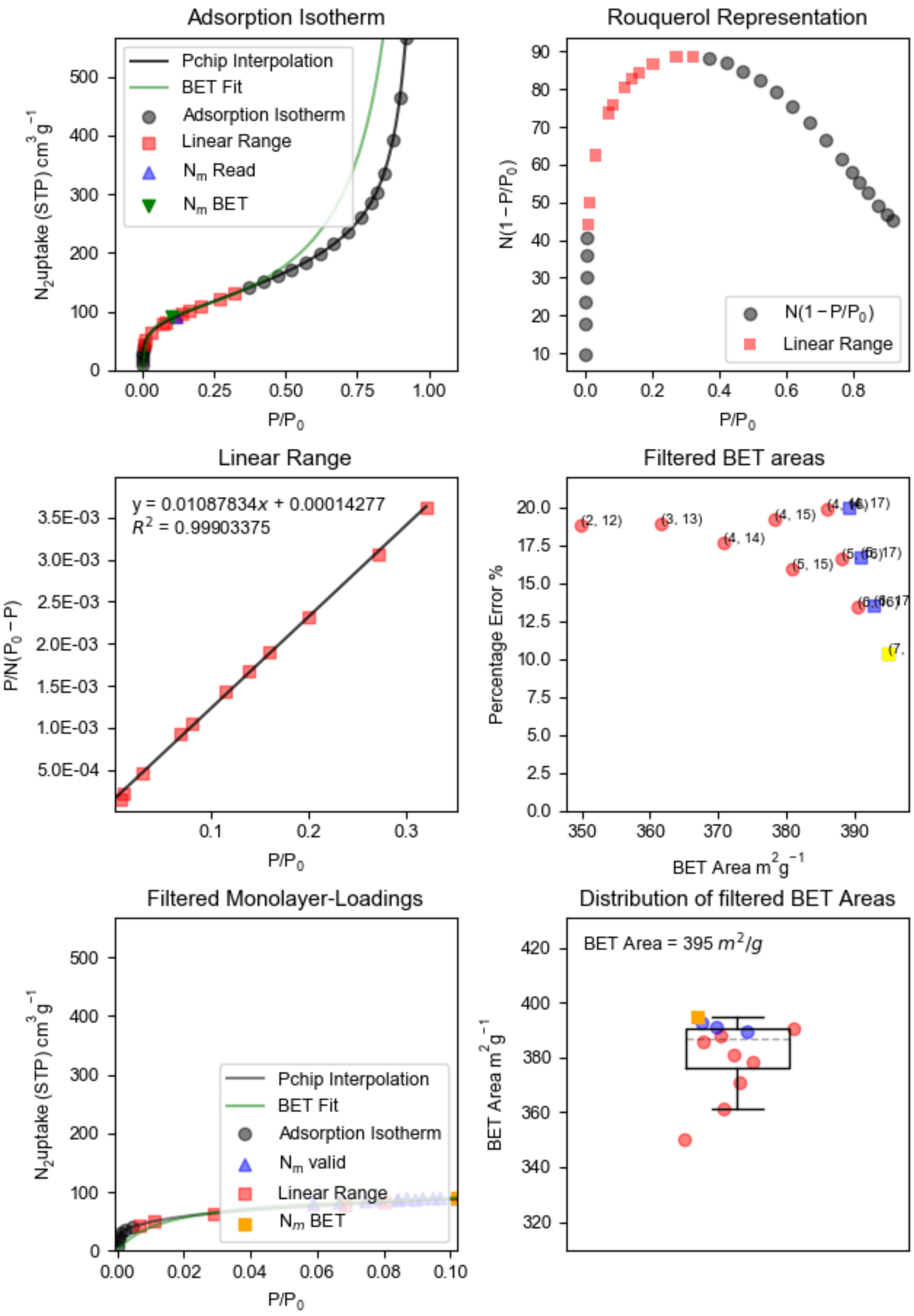

Figure S19. BETSI analysis for BCN-11_21.

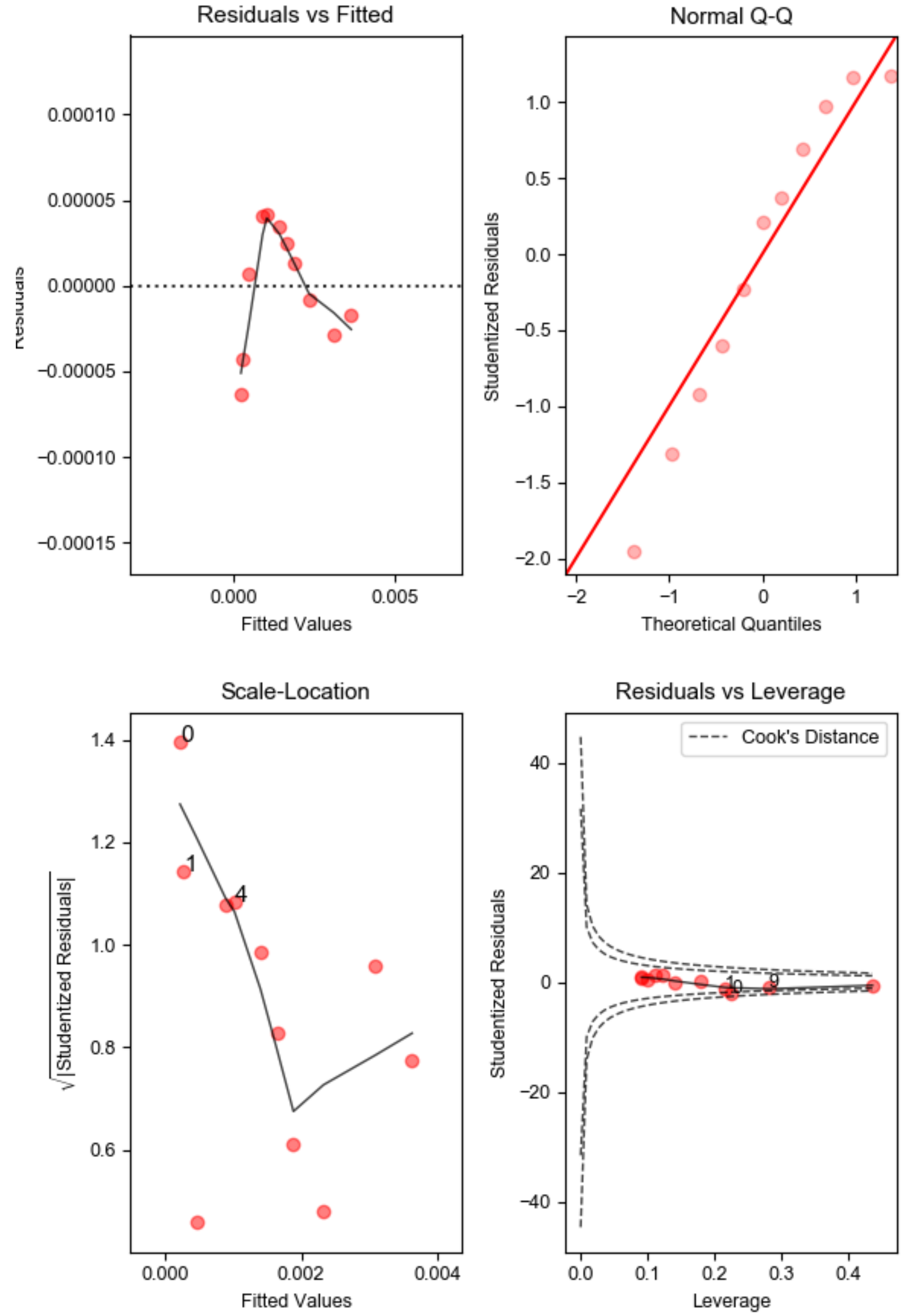

Figure S20. BETSI regression diagnostics for BCN-11_21.

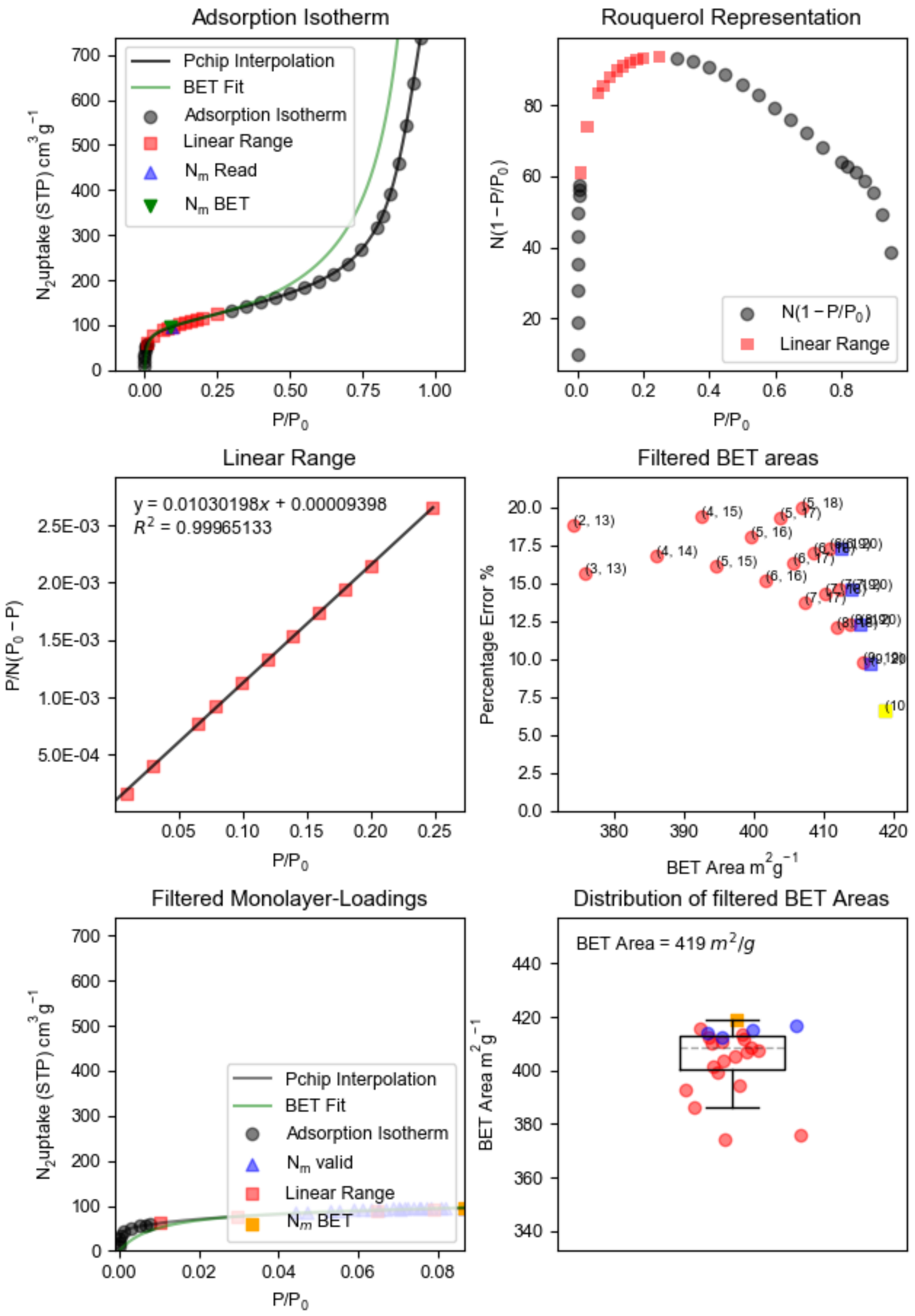

Figure S21. BETSI analysis for BCN-11_35.

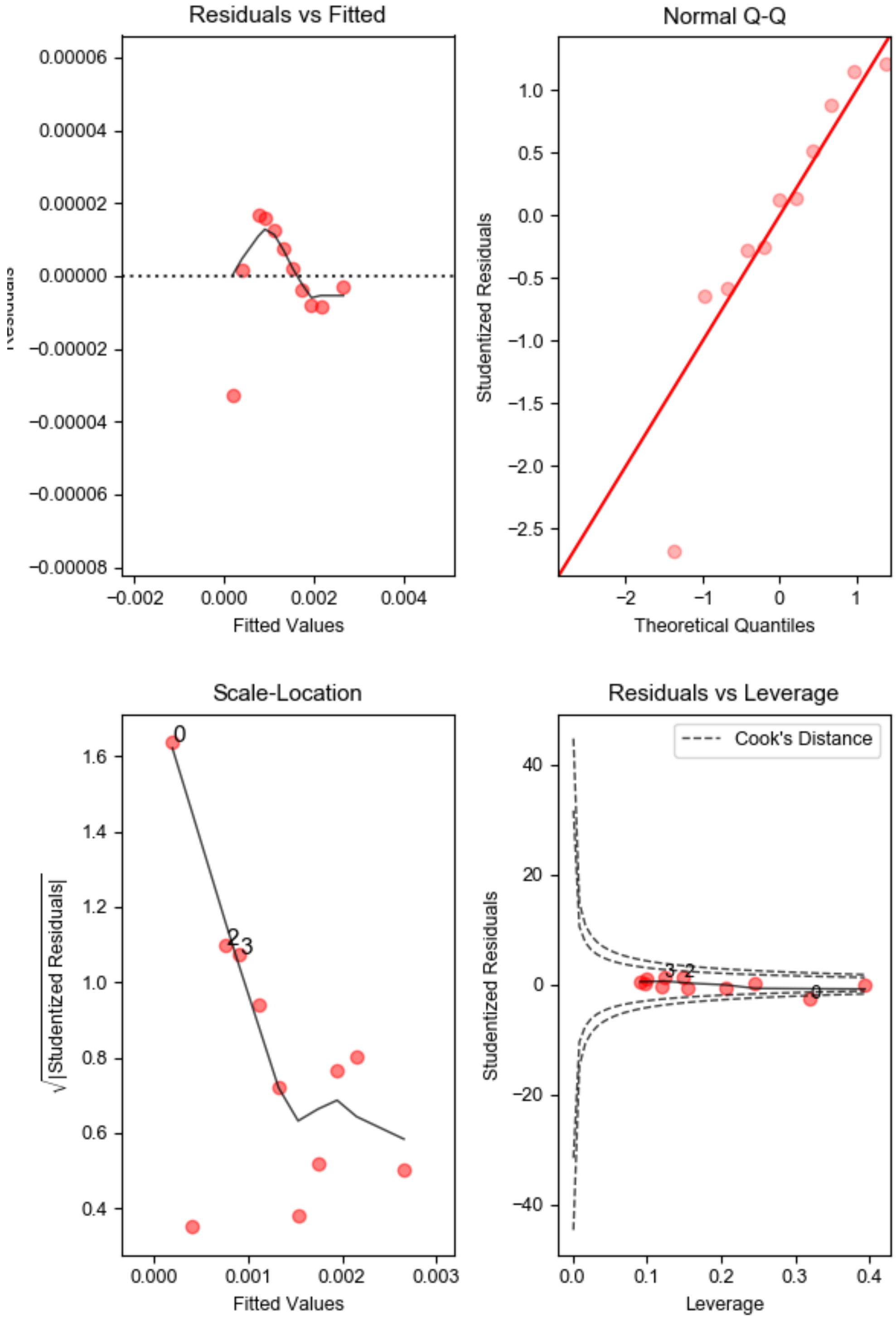

Figure S22. BETSI regression diagnostics for BCN-11_35.

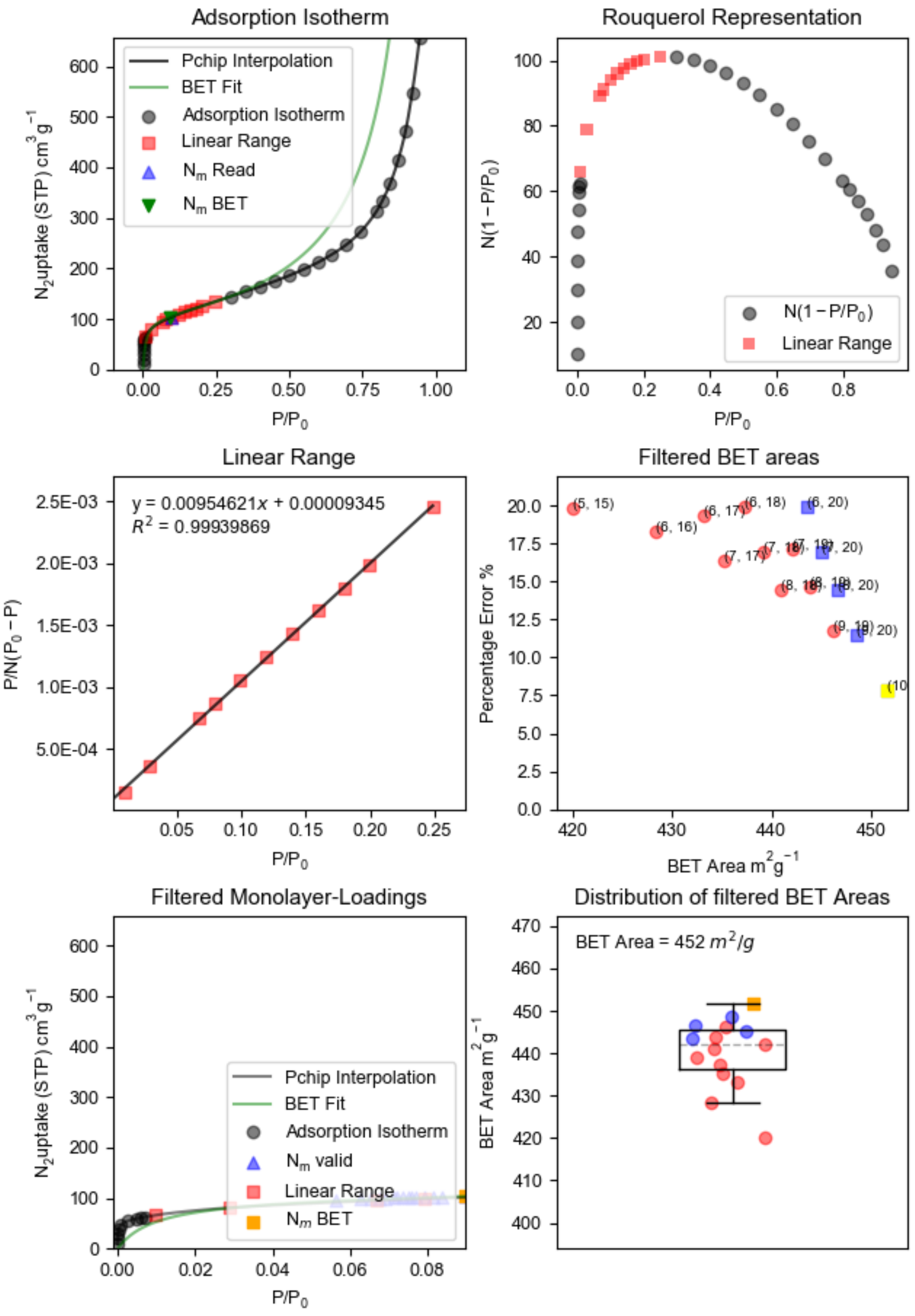

Figure S23. BETSI analysis for BCN-11_42.

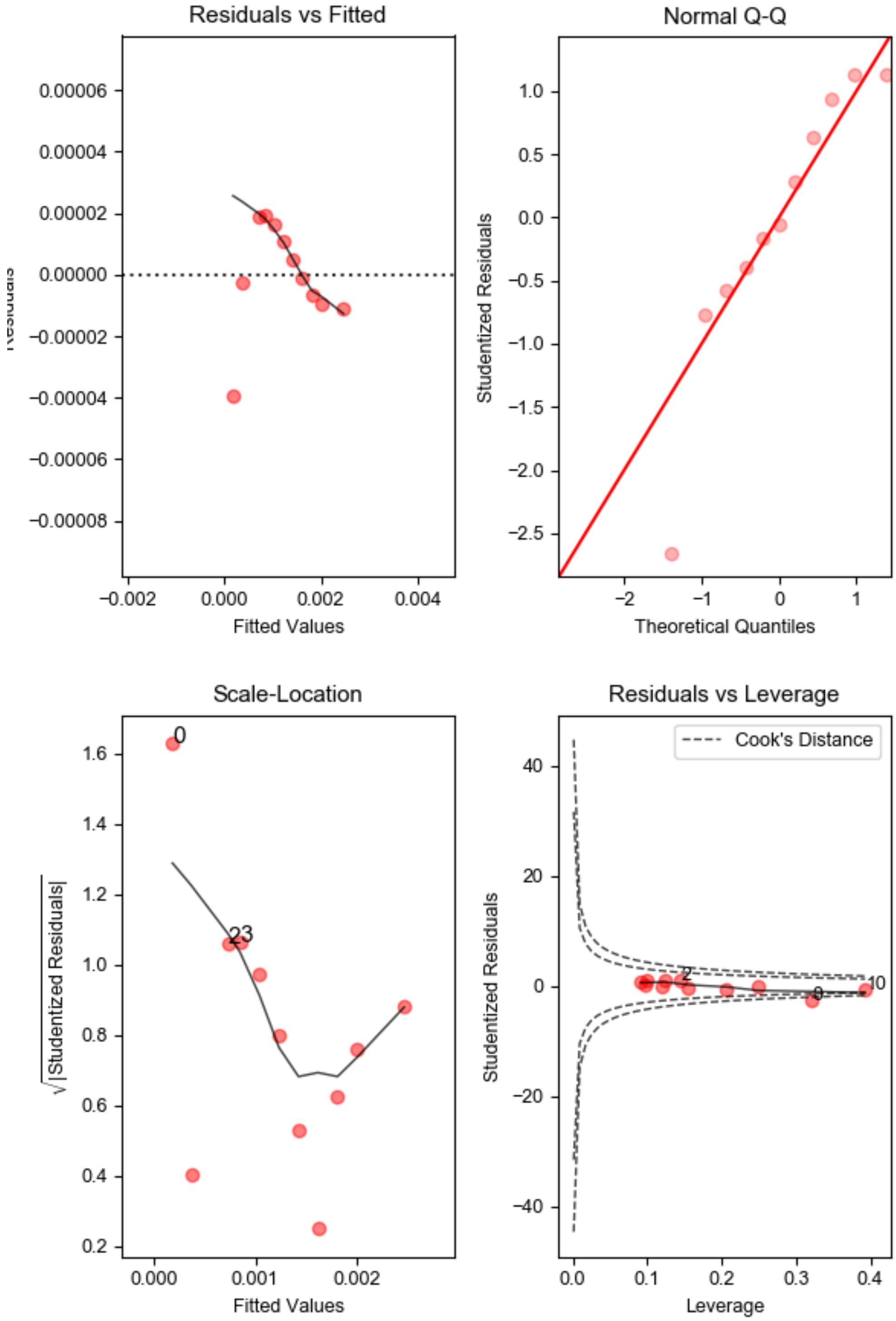

Figure S24. BETSI regression diagnostics for BCN-11_42.

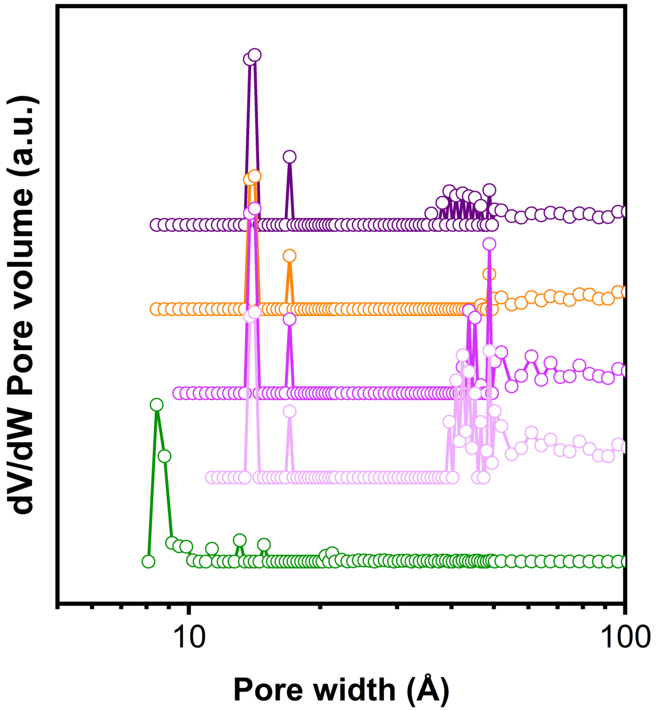

Figure S25. Pore size distribution (PSD) estimated by DFT for OH-RhMOP (green), BCN-11_13 (light pink), BCN-11_21 (pink), BCN-11_35 (orange), and BCN-11_42 (purple). The PSD was estimated using the MicroActive Version 4.06 software, employing Density Functional Theory (DFT) with a cylindrical pore model for oxide surfaces. The PSD was then optimized to reduce roughness, achieving the smallest possible Root Mean Square (RMS) Error fit.

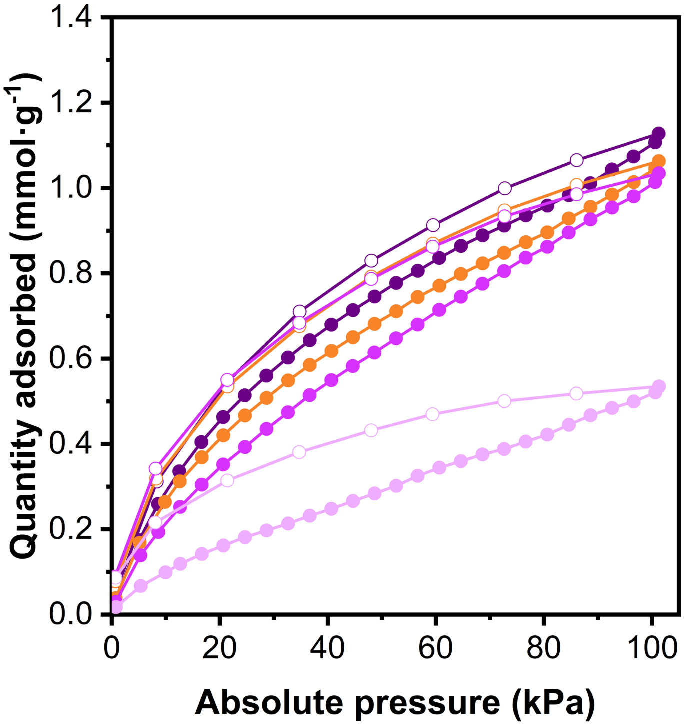

Figure S26. CO_2 adsorption isotherms at 298 K for BCN-11_13 (light pink), BCN-11_21 (pink), BCN-11_35 (orange), and BCN-11_42 (purple).

Table S2. Swelling and ICP-MS data of the supernatant after the swelling process.

Aerogel	Swelling ratio	Leached Rh ($mg \cdot L^{-1}$)	Leached P ($mg \cdot L^{-1}$)	Volume	Released DNA (% w/w)	Released MOP (% w/w)
**BCN-11_13**	968	0.85	0.02	120.6	0.99	0.59
**BCN-11_21**	346	0.78	0.01	138.4	0.99	0.59
**BCN-11_35**	64	0.74	0.01	147.3	0.99	0.8
**BCN-11_42**	23	0.19	0.02	148.9	0.99	0.96

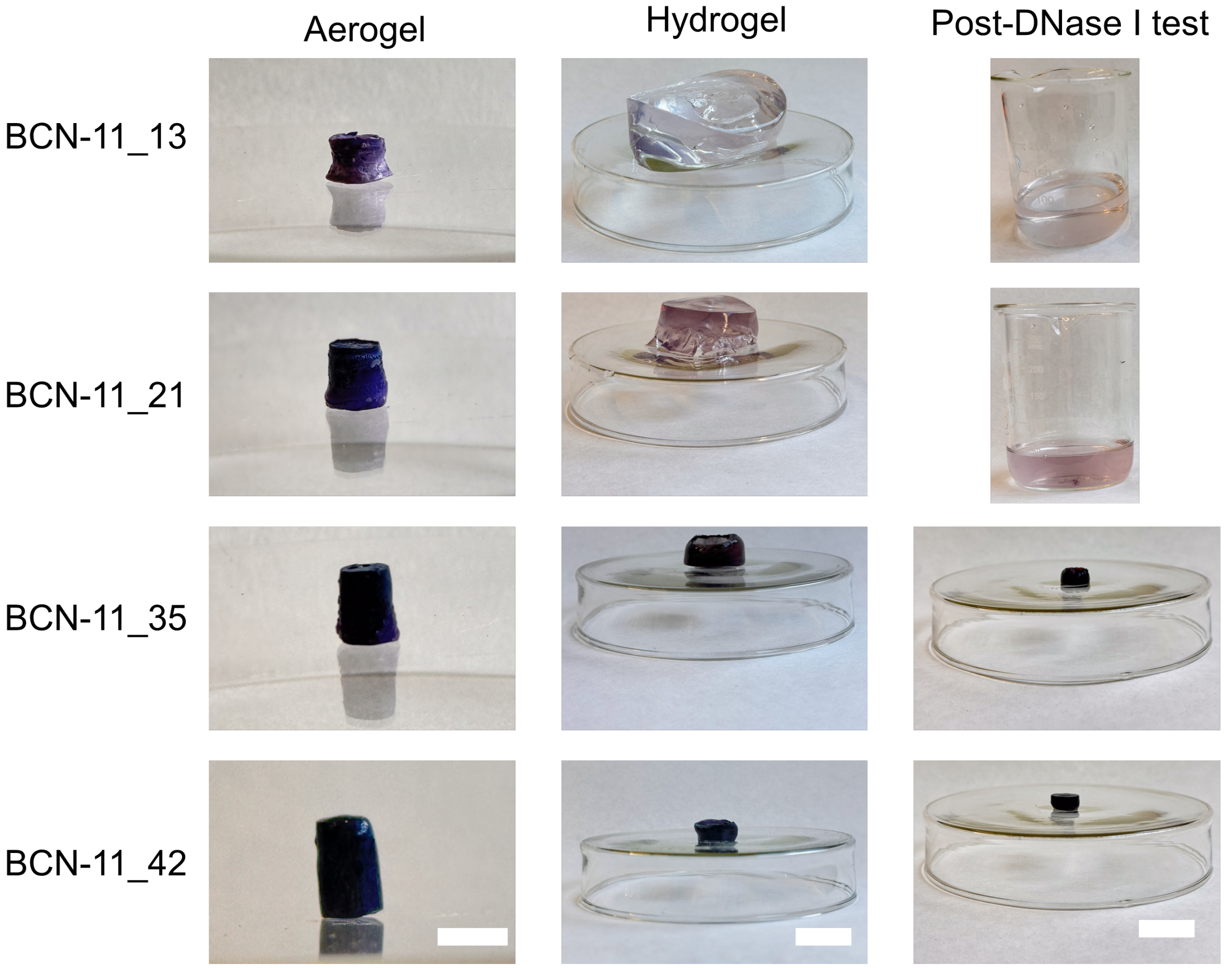

Figure S27. Photographs of BCN-11 aerogels (left, scale bar: 0.5 cm), swelled hydrogels in water (center, scale bar: 2 cm) and the obtained gels/solution after incubating the hydrogels in the digestion solution containing DNase I (right, scale bar: 2 cm). The shrinkage observed in the case of BCN-11_35 and BCN-11_42 upon incubation in the digestion solution is ascribed to the high ionic strength of this solution. Rh and P were not detected in the supernatant of the digestion solution of BCN-11_35 and BCN-11_42, confirming the stability of both gels in the presence of DNase I.

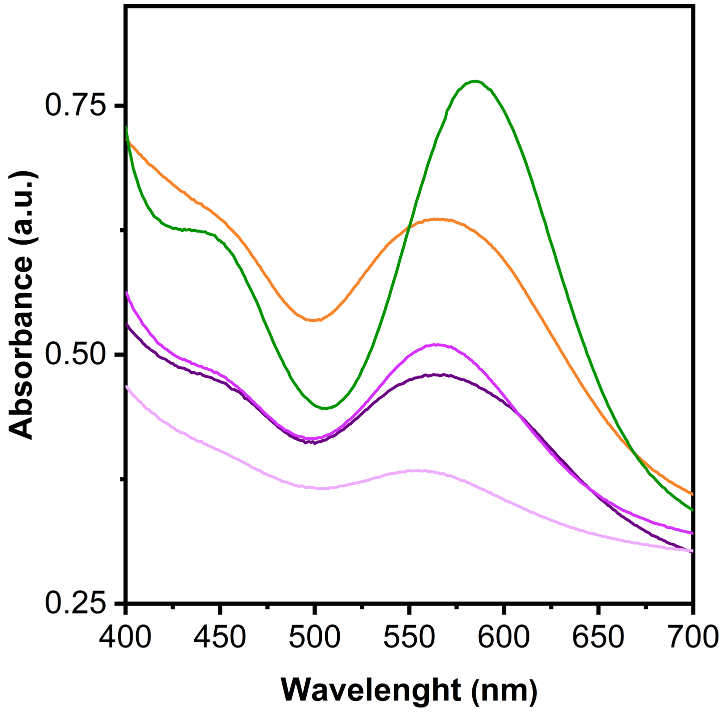

Figure S28. Solid-state UV-Vis absorption spectra for DNA-MOP hydrogel (green), BCN-11_13 hydrogel (light pink), BCN-11_21 hydrogel (pink), BCN-11_35 hydrogel (orange), and BCN-11_42 hydrogel (purple). Note that the characteristic absorption band of the Rh-Rh bond (λ_{max}) centered at 586 nm for ONa-RhMOP is shifted to *ca.* 564 nm for all the BCN-11 derived hydrogels, confirming coordinative interaction between DNA and ONa-RhMOP in the hydrogel state.

.

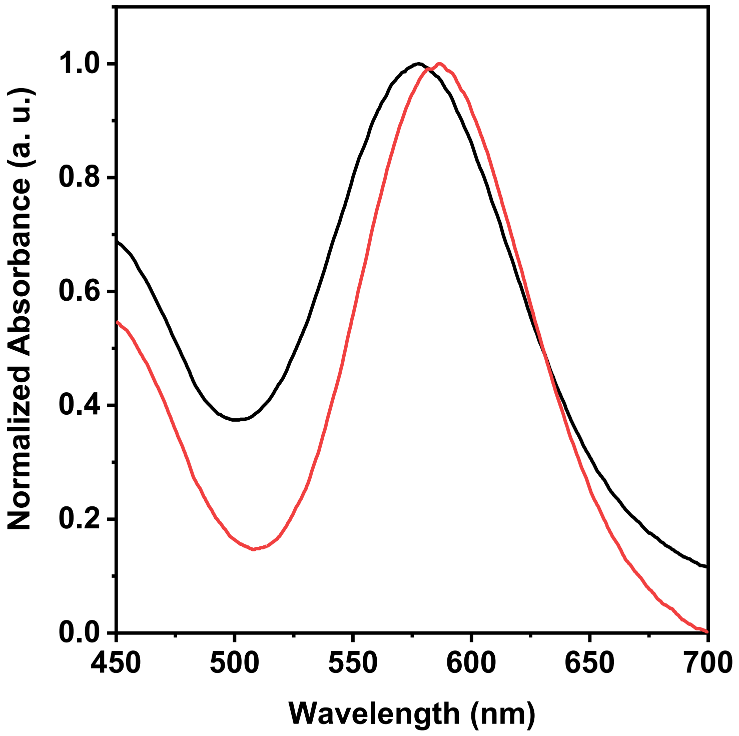

Figure S29. UV-Vis spectra of ONa-MOP (red) and ONa-MOP with 6 mol. eq. of adenosine monophosphate (black) in H_2O (pH = 11) after exposing the reaction mixture to methanol, as detailed above in the synthetic procedure. The shift in λ_{max}, from 587 nm (red) to 578 nm (black), is attributed to the coordination between adenosine monophosphate and the Rh(II) open metal site of ONa-MOP.

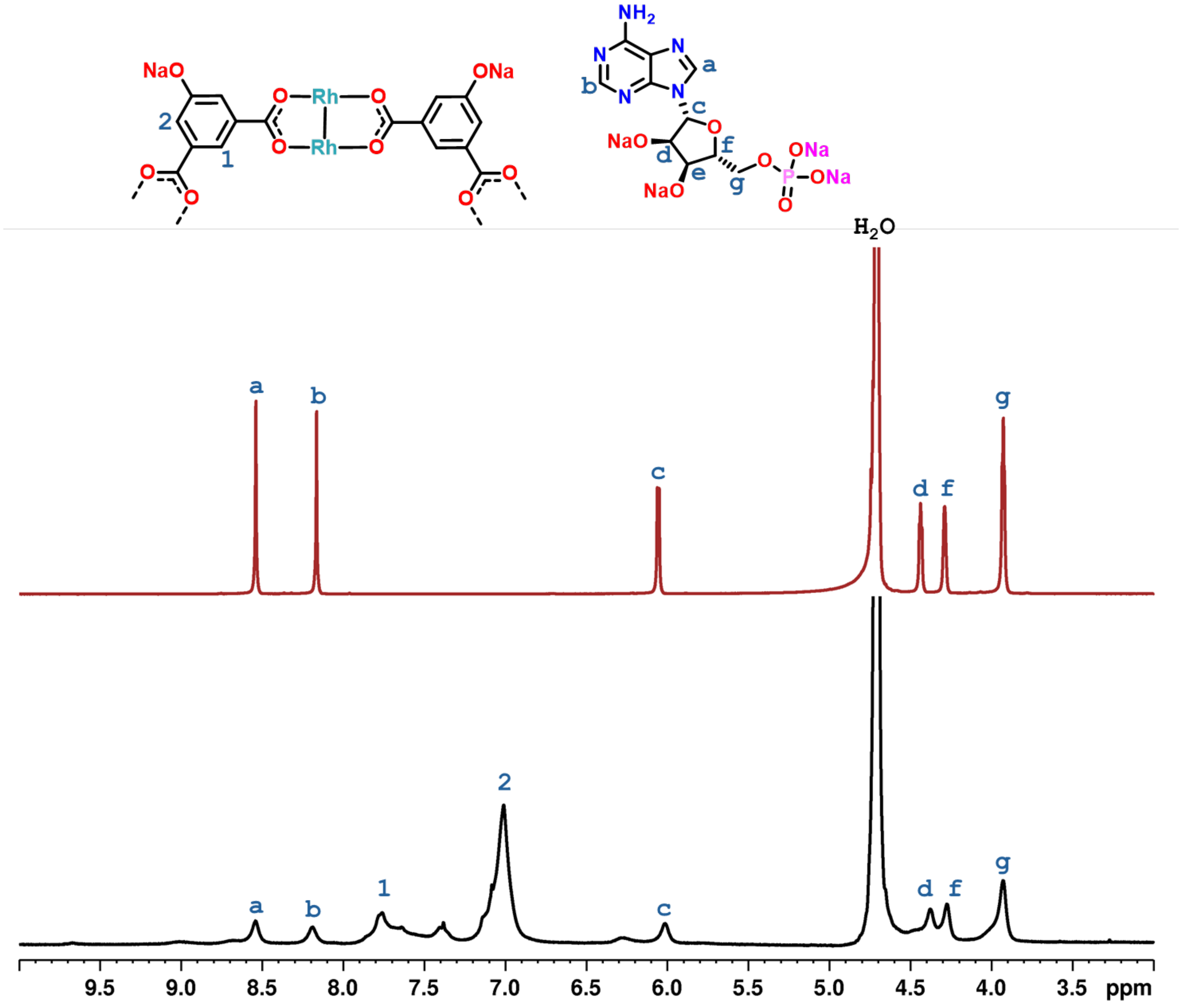

Figure S30. ^{1}H-NMR spectra (500 MHz, 25ºC) of adenosine monophosphate (top) and a mixture of ONa-MOP and adenosine monophosphate (6 mol. eq.) (bottom) in D_2O (pD = 11) after exposing the reaction mixture to methanol, as detailed above in the synthetic procedure. Signal e is overlapped with the signal of H_2O. Note that, in the spectrum of the mixture (bottom), the signals of adenosine monophosphate are broadened and shifted upfield, indicating that adenosine monophosphate is interacting with ONa-RhMOP through metal-coordination.

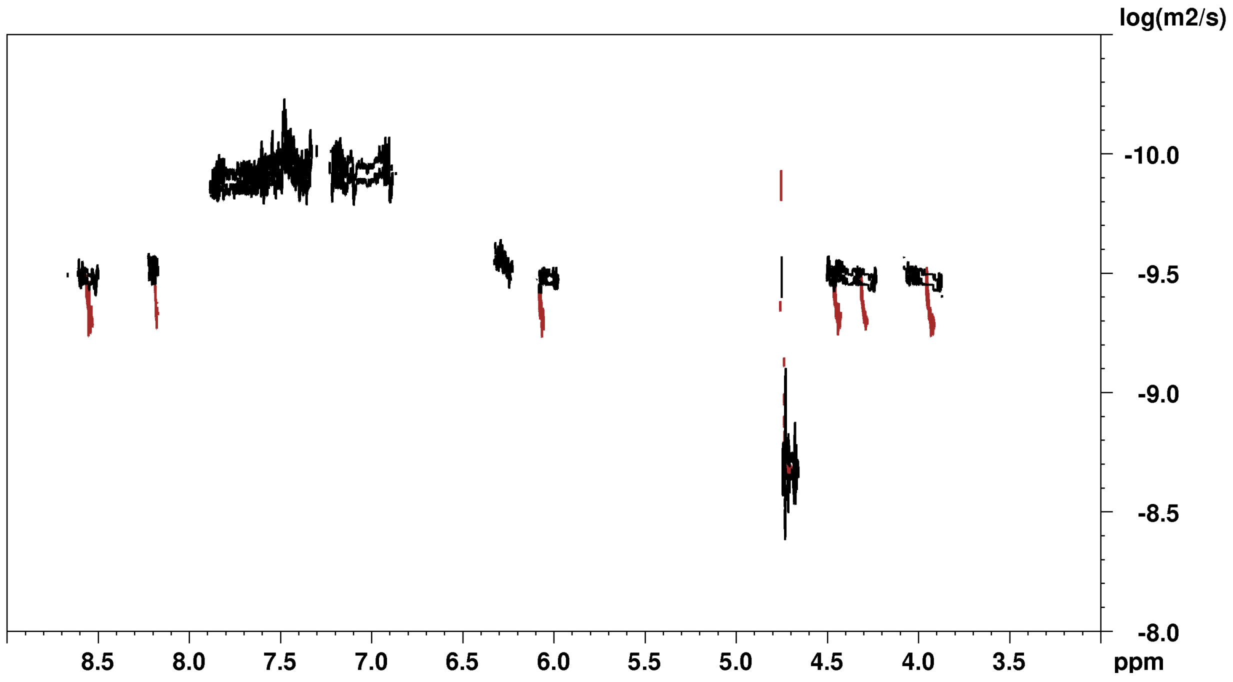

Figure S31. DOSY-NMR spectra (500 MHz, 25ºC) of adenosine monophosphate (red) and the mixture of ONa-MOP and adenosine monophosphate (6 mol. eq.) (black) in D_2O (pD = 11) after exposing the reaction mixture to methanol, as detailed above in the synthetic procedure. The decrease in diffusion coefficient of adenosine monophosphate in the presence of ONa-RhMOP further confirms its coordination to the MOP.

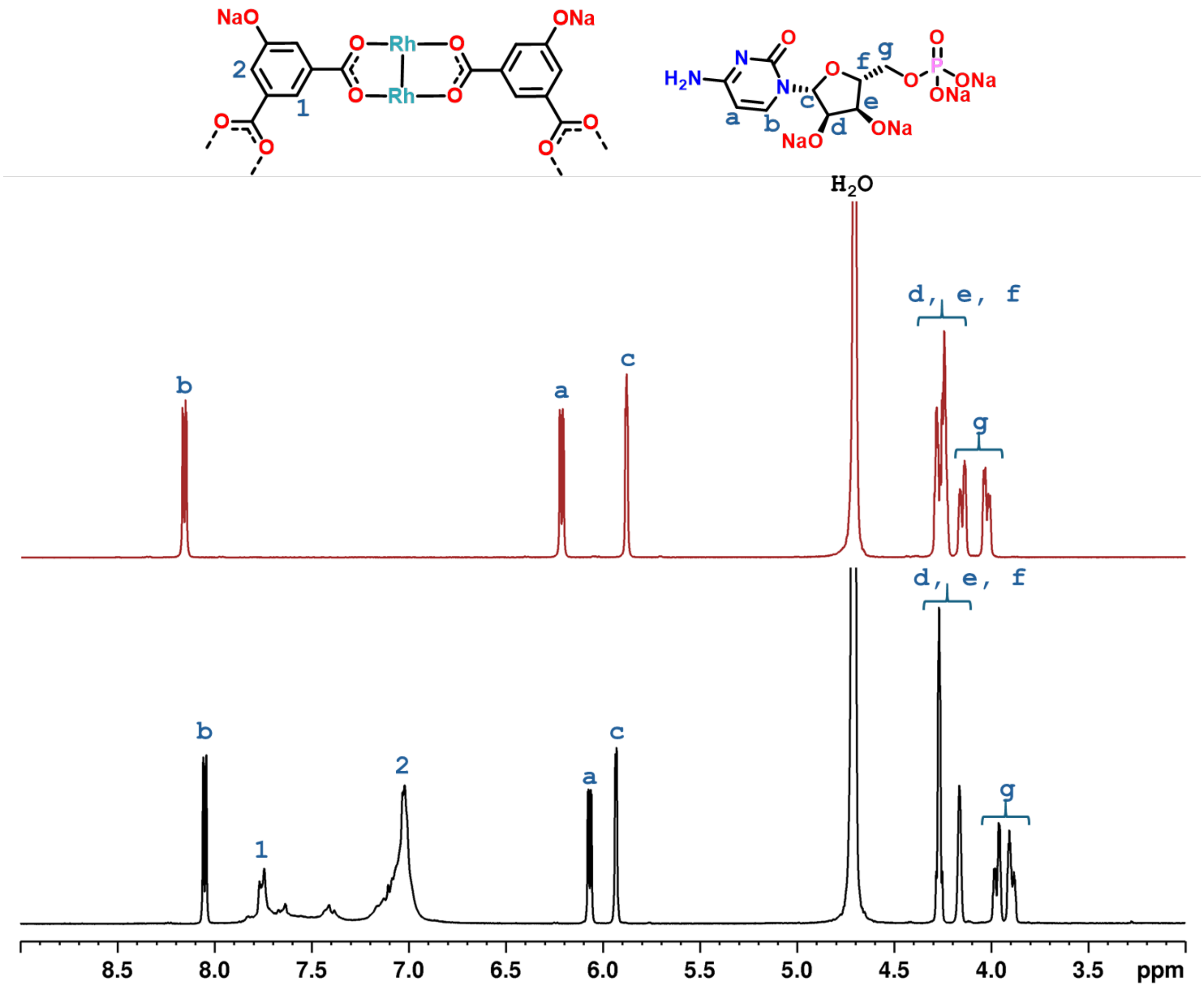

Figure S32. ^{1}H-NMR spectra (500 MHz, 25ºC) of cytidine monophosphate (top) and a mixture of ONa-MOP and cytidine monophosphate (6 mol. eq.) (bottom) in D_2O (pD = 11) after exposing the reaction mixture to methanol, as detailed above in the synthetic procedure. In the spectrum of the mixture (bottom), the signals of cytidine monophosphate are not broadened or shifted, indicating that there is not coordinative interaction between the nucleotide and the MOP.

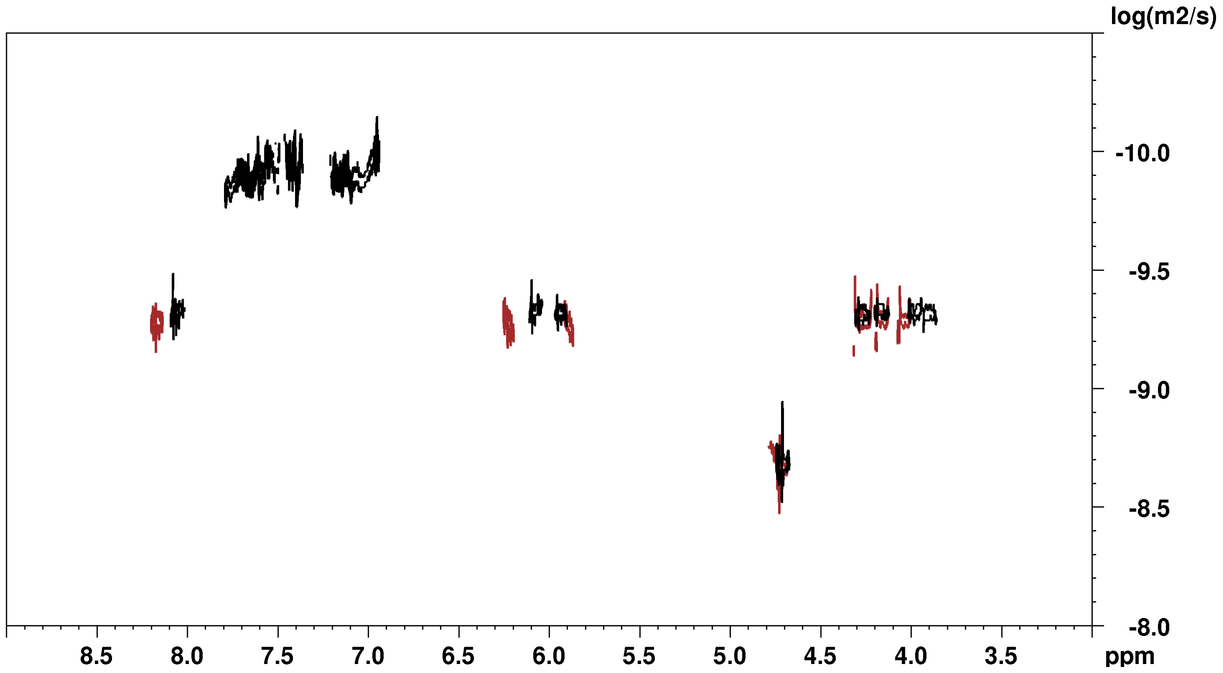

Figure S33. DOSY-NMR spectra (500 MHz, 25ºC) of cytidine monophosphate (red) and the mixture of ONa-MOP and cytidine monophosphate (6 mol. eq.) (black) in D_2O (pD = 11) after exposing the reaction mixture to methanol, as detailed above in the synthetic procedure. The diffusion coefficient of cytidine monophosphate does not change upon addition of ONa-RhMOP, indicating the absence of strong interactions between the nucleotide and the MOP.

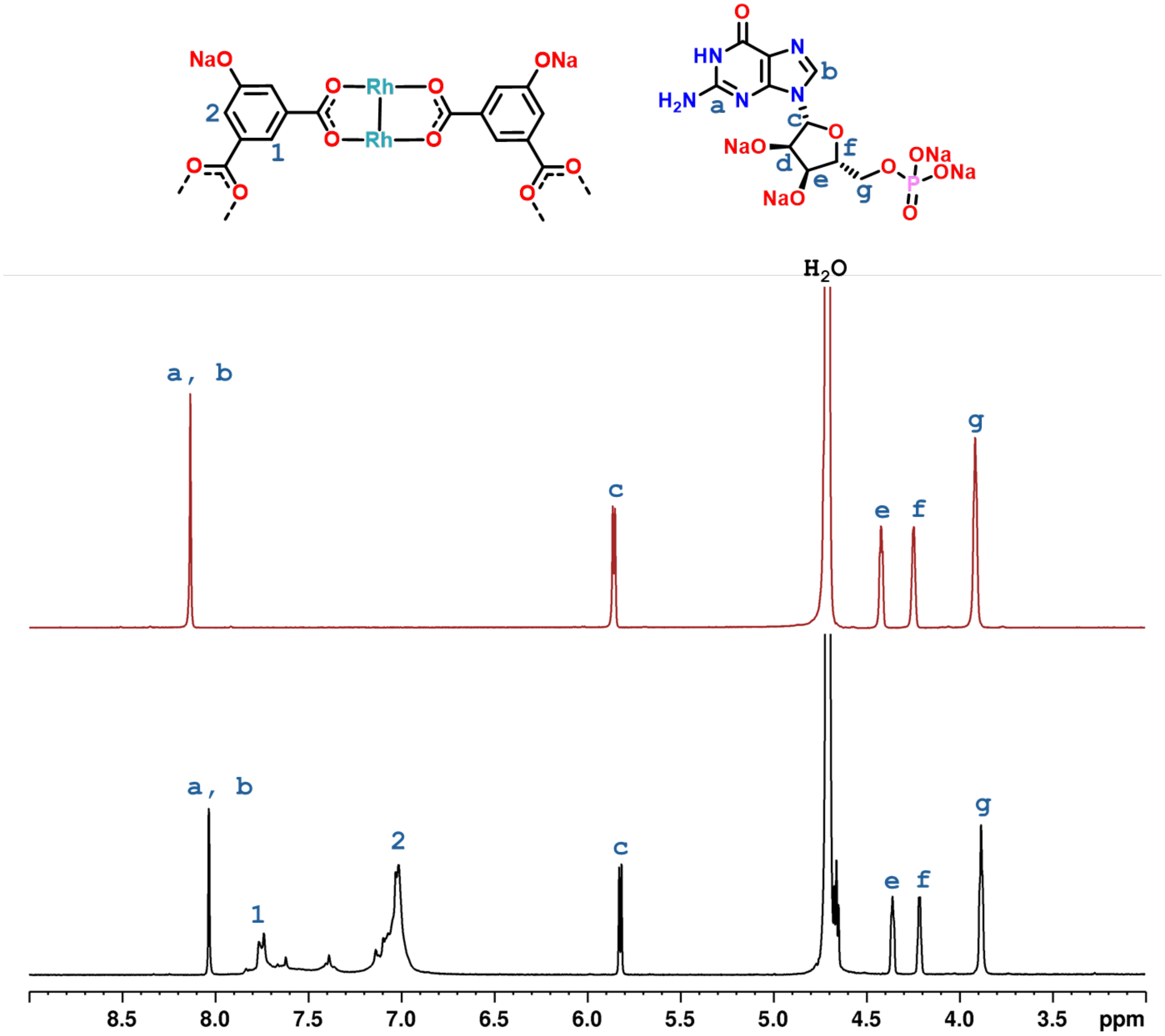

Figure S34. ^{1}H-NMR spectra (500 MHz, 25ºC) of guanosine monophosphate (top) and a mixture of ONa-MOP and guanosine monophosphate (6 mol. eq.) (bottom) in D_2O (pD = 11) after exposing the reaction mixture to methanol, as detailed above in the synthetic procedure. In the spectrum of the mixture (bottom), the signals of guanosine monophosphate are not broadened or shifted, indicating that there is not coordinative interaction between the nucleotide and the MOP.

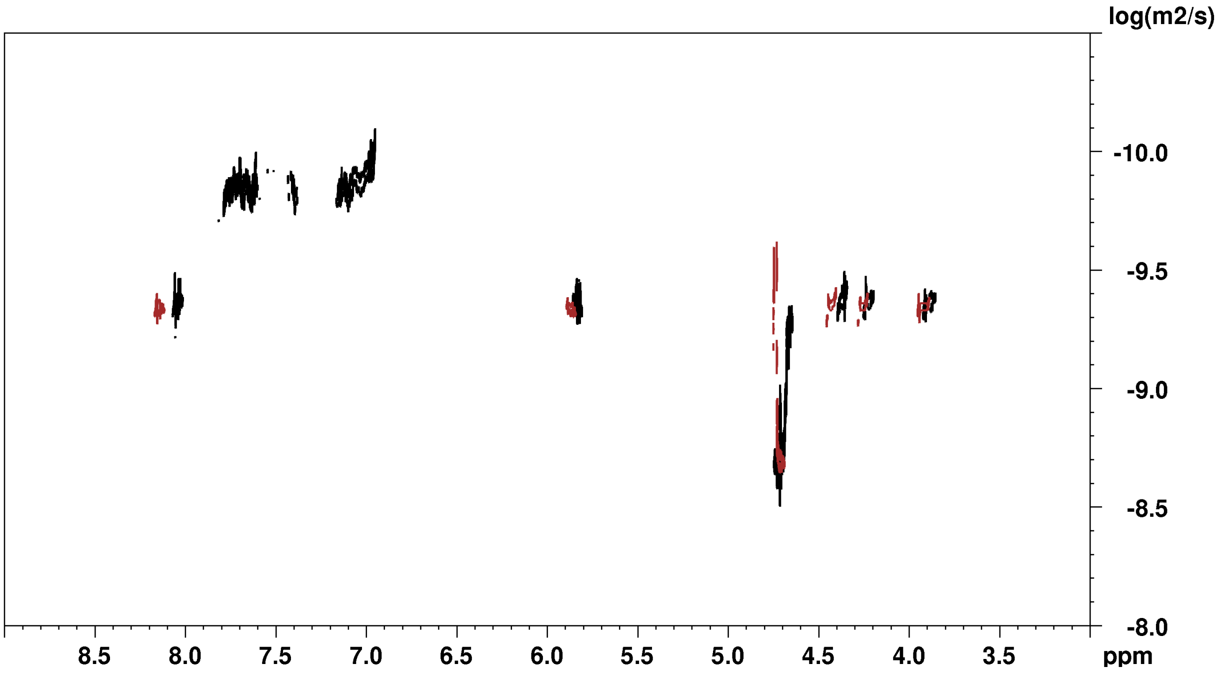

Figure S35. DOSY-NMR spectra (500 MHz, 25ºC) of guanosine monophosphate (red) and the mixture of ONa-MOP and guanosine monophosphate (6 mol. eq.) (black) in D_2O (pD = 11) after exposing the reaction mixture to methanol, as detailed above in the synthetic procedure. The diffusion coefficient of guanosine monophosphate does not change upon addition of ONa-RhMOP, indicating the absence of strong interactions between the nucleotide and the MOP.

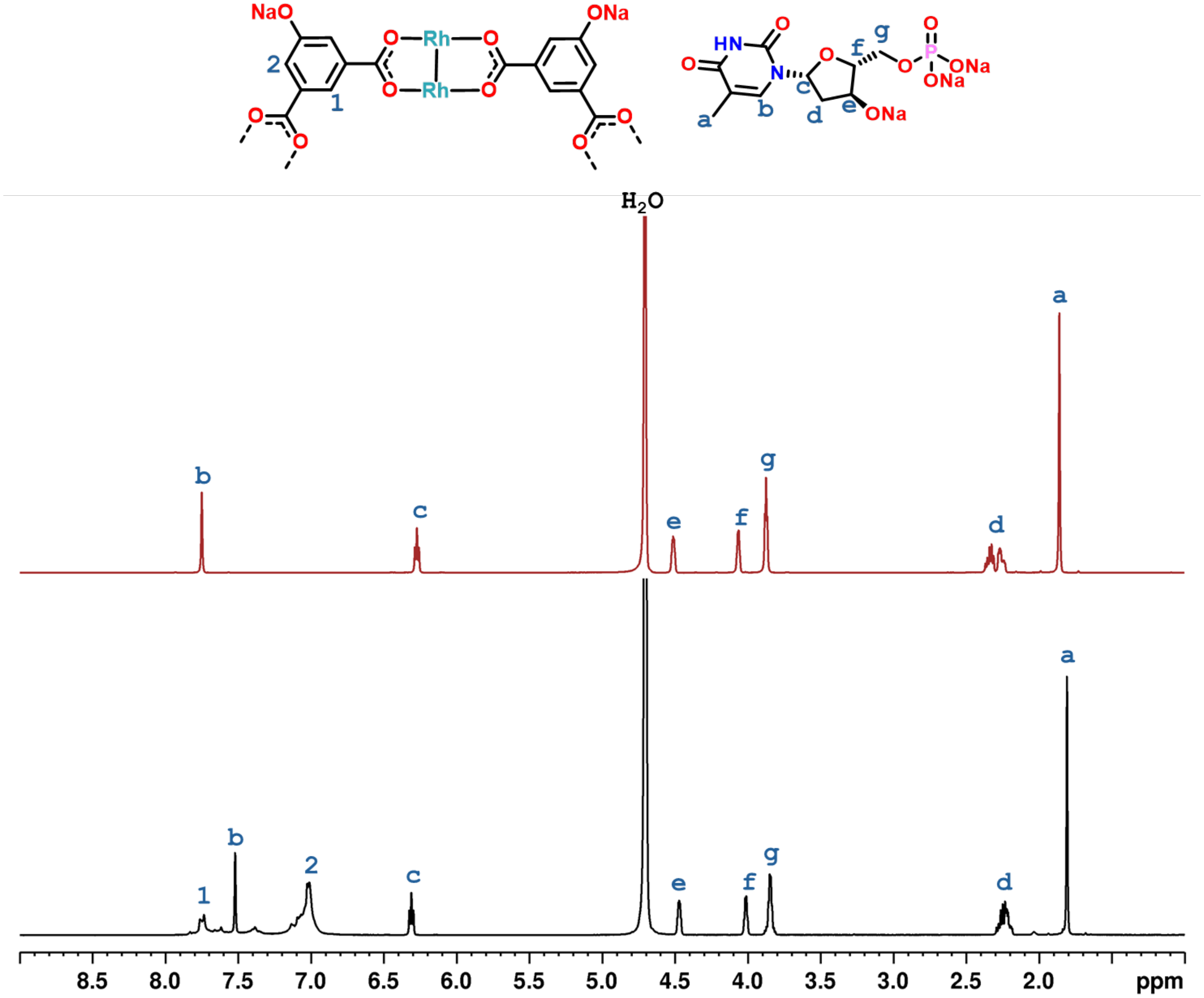

Figure S36. ^{1}H-NMR spectra (500 MHz, 25ºC) of thymidine monophosphate (top) and a mixture of ONa-MOP and thymidine monophosphate (6 mol. eq.) (bottom) in D_2O (pD = 11) after exposing the reaction mixture to methanol, as detailed above in the synthetic procedure. In the spectrum of the mixture (bottom), the signals of thymidine monophosphate are not broadened or shifted, indicating that there is not coordinative interaction between the nucleotide and the MOP.

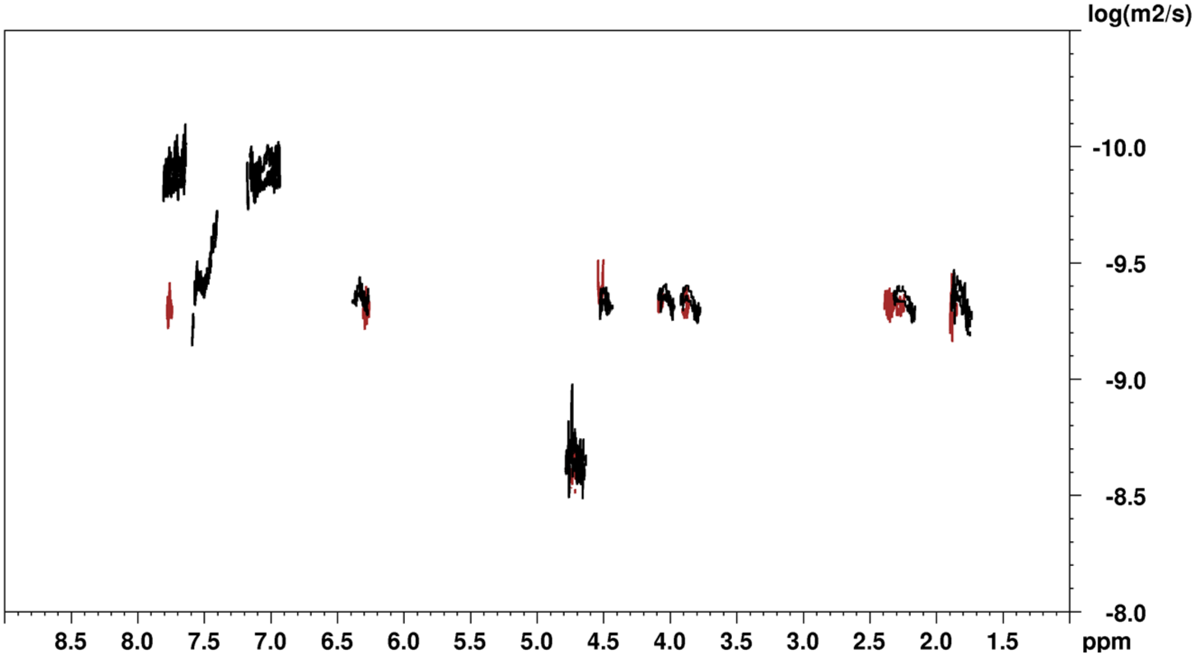

Figure S37. DOSY-NMR spectra (500 MHz, 25ºC) of thyimidine monophosphate (red) and the mixture of ONa-MOP and thyimidine monophosphate (6 mol. eq.) (black) in D_2O (pD = 11) after exposing the reaction mixture to methanol, as detailed above in the synthetic procedure. The diffusion coefficient of thyimidine monophosphate does not change upon addition of ONa-RhMOP, indicating the absence of strong interactions between the nucleotide and the MOP.

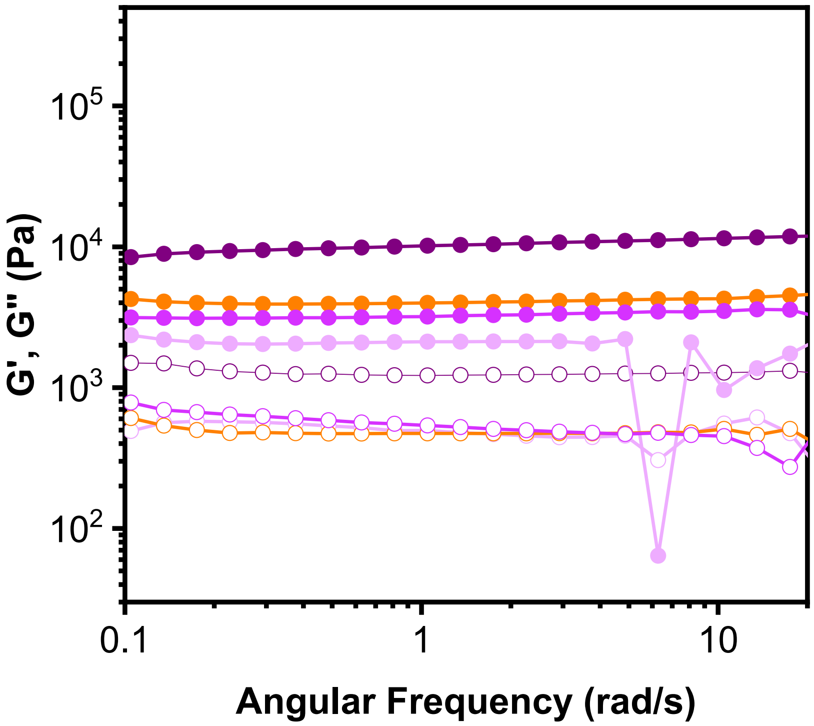

Figure S38. Shear storage modulus (G’) (filled circles) and loss storage modulus (G”) (hollow circles) of BCN-11_13 (light pink), BCN-11_21 (pink), BCN-11_35 (orange), and BCN-11_42 (purple).

Table S3. Summary of the mechanical properties of most relevant DNA-based hydrogels on the literature.

TYPE OF DNA	CROSSLINKING NODE	CROSSLINKING INTERACTION	STORAGE MODULUS (G´, PA)	REFERENCE
Synthetic oligonucleotides containing poly(a)	Cyanuric acid (Cy)	A-Cy H-bonding	10^5	4
Synthetic oligonucleotides	-	Base paring	4000	5
Synthetic oligonucleotides	Y scaffold	Base paring	1800	6
Synthetic oligonucleotides	-	Base paring	1800	7
Synthetic oligonucleotides	-	Base paring	2800	8
Synthetic oligonucleotides	Y scaffold	Base paring	1500	9
Synthetic oligonucleotides	Y scaffold	Base paring	500	10
Synthetic oligonucleotides	DNA-functionalized Magnetic nanoparticles	Base paring	1000	11
Rca dna	Upconversion nanoparticles	Metal cooridnation	50	12
Pcr elongated dna	Gold nanoparticles	Base paring + thiol-Au interactions	1000	13
Salmon sperm dna	Upconversion nanoparticles	Metal cooridnation	180	12
Salmon sperm dna	G-quadruplex	Physical crosslinking	50	14
Dna derived from onion	DNA derived carbon dots	Base paring	400	15
Salmon sperm	Inonomers	Electrostatic	10	16
Dna derived from fish sperm	Graphene oxide	π−π stacking and hydrophobic interactions	4600	17
Dna from salmon testes	Polyethylene glycol diglycidyl ethe and silicate nanodisks	Covalent bonding and electrostatic interactions	1100	18
Dna from salmon testes	1,1′ -diheptyl-4,4′ -bipyridinium	Groove binding	10000	19
Dna from salmon testes	poly(ethylene glycol)diglycidyl ether + calcium-alginate	Covalent + electrostatic interactions	10000	20
Dna from salmon testes	two-dimensional silicate nanodisks	Electrostatic	460	21
Salmon sperm dna	MOP	Metal coordination	2100 - 10100	This work

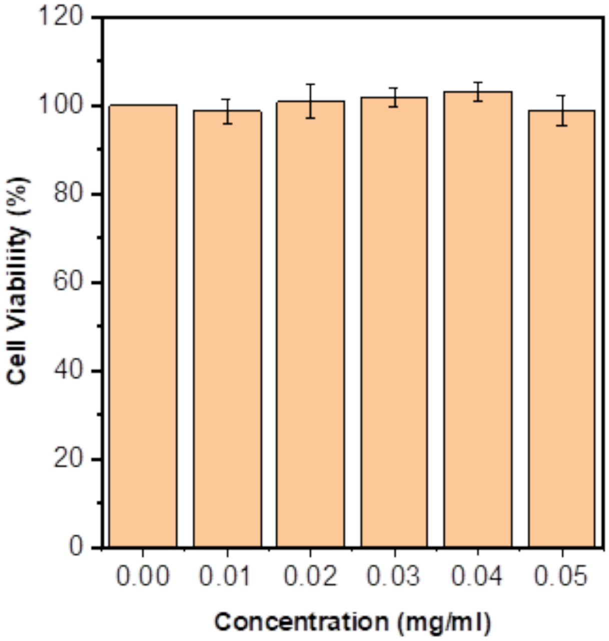

Figure S39. Cell viability study of the degradation products obtained after exposing BCN-11_13 to DNase I. Cell line employed for the cell viability assay was 1BR3G human skin fibroblasts.

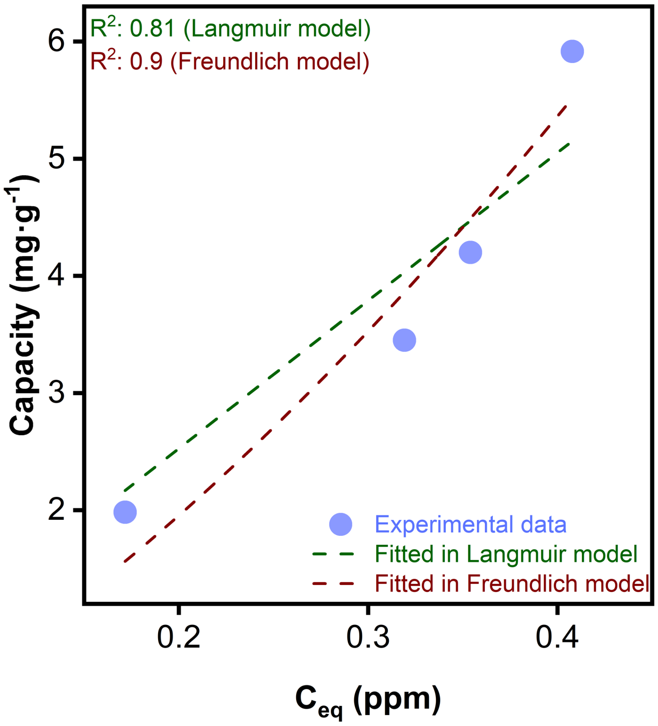

Figure S40. Non-linear fitting of Langmuir and Freundlich adsorption isotherm models for the removal of Acridine Yellow at time duration of 24 h with the BCN-11_42 hydrogel.

Table S4. Langmuir and Freundlich adsorption isotherm parameters obtained by nonlinear fitting for the capture of Acridine Yellow within the BCN-11_42 hydrogel.

Adsorption isotherm model	R^2	χ^2	n
Langmuir model	0.81	0.51	-
Freundlich model	0.9	0.29	0.68

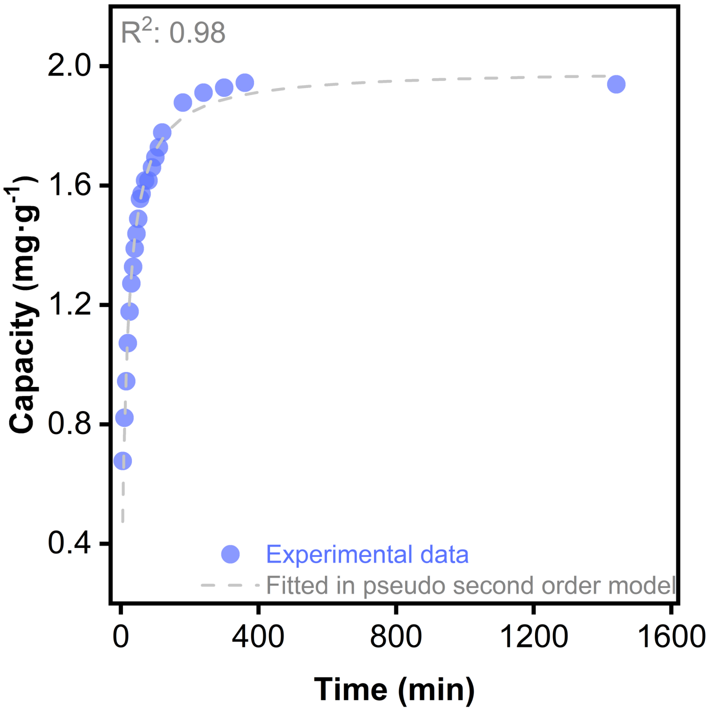

Figure S41. Adsorption kinetic for the capture of Acridine Yellow (initial concentration: 4 ppm) within the BCN-11_42 hydrogel fitted in pseudo second-order kinetic model.

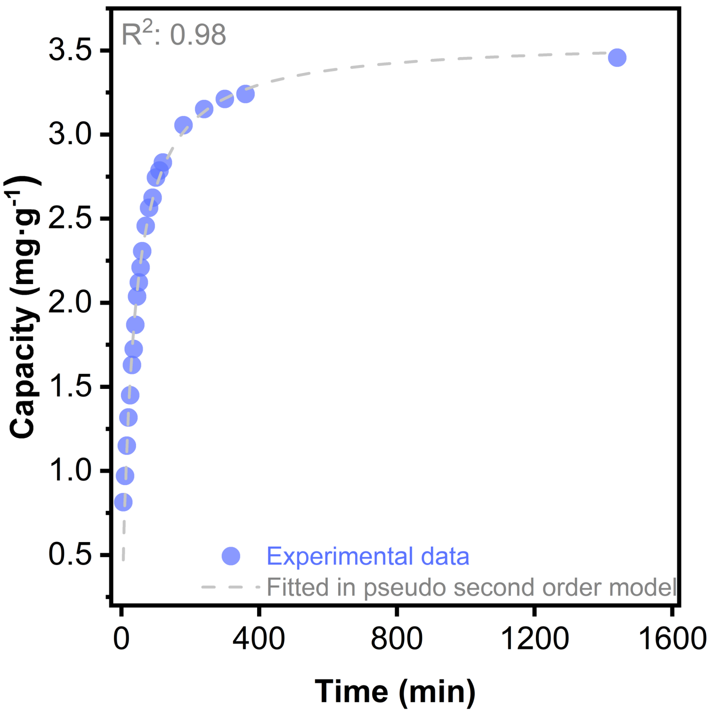

Figure S42. Adsorption kinetic for the capture of Acridine Yellow (initial concentration: 6 ppm) within the BCN-11_42 hydrogel fitted in pseudo second-order kinetic model.

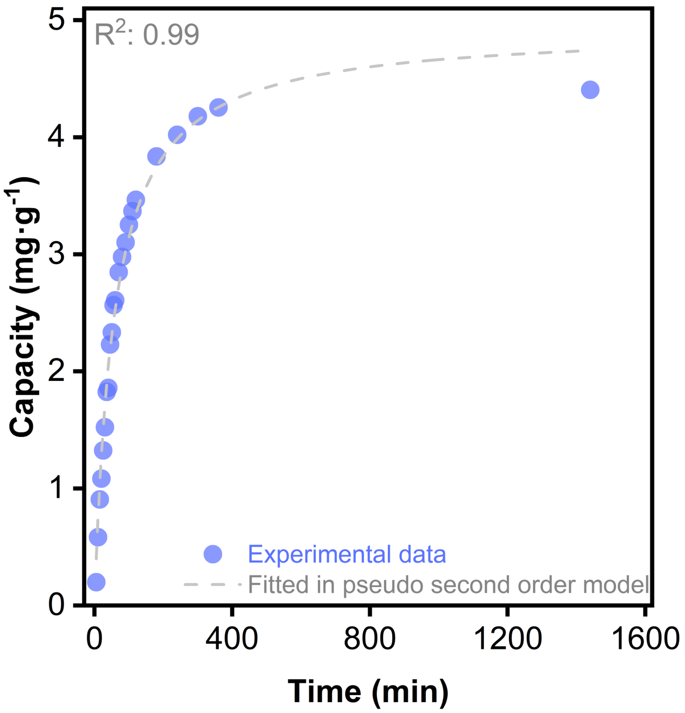

Figure S43. Adsorption kinetic for the capture of Acridine Yellow (initial concentration: 7 ppm) within the BCN-11_42 hydrogel fitted in pseudo second-order kinetic model.

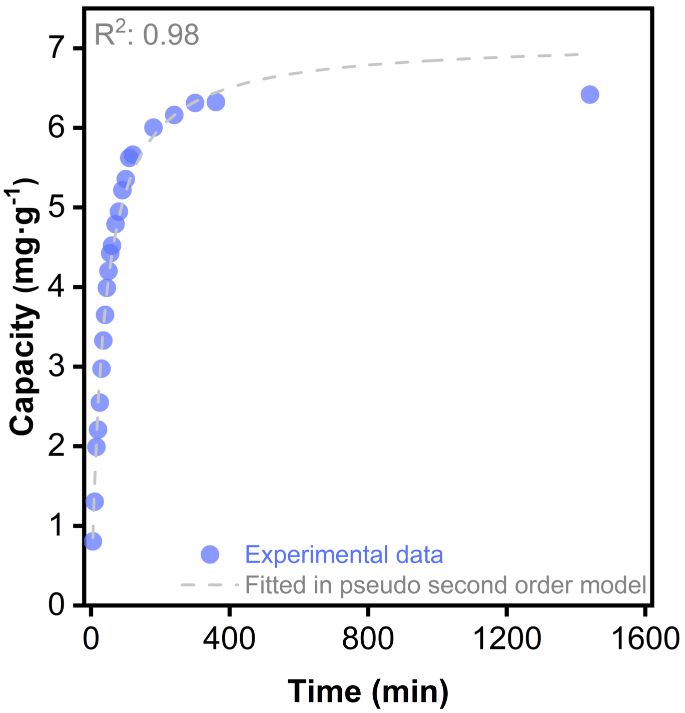

Figure S44. Adsorption kinetic for the capture of Acridine Yellow (initial concentration: 10 ppm) within the BCN-11_42 hydrogel fitted in pseudo second-order kinetic model.

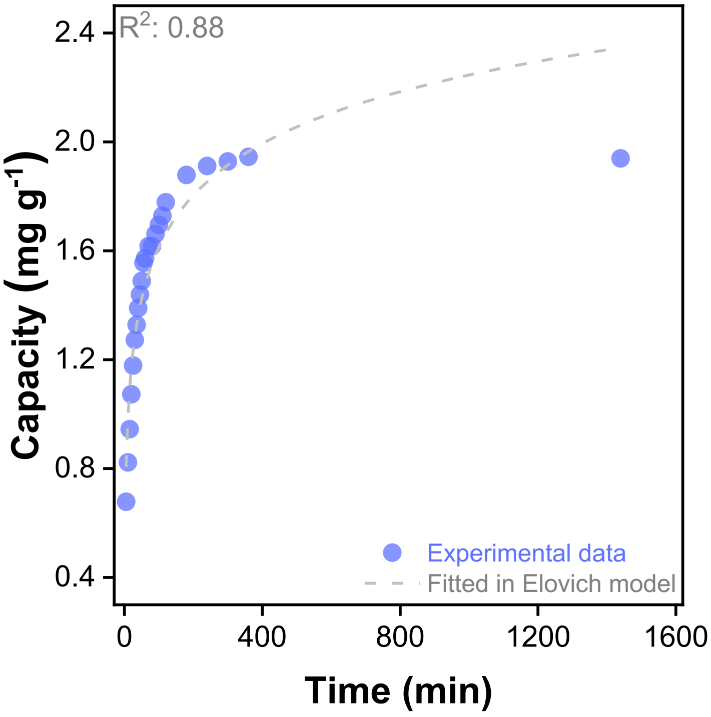

Figure S45. Adsorption kinetic for the capture of Acridine Yellow (initial concentration: 4 ppm) within the BCN-11_42 hydrogel fitted to the Elovich model.

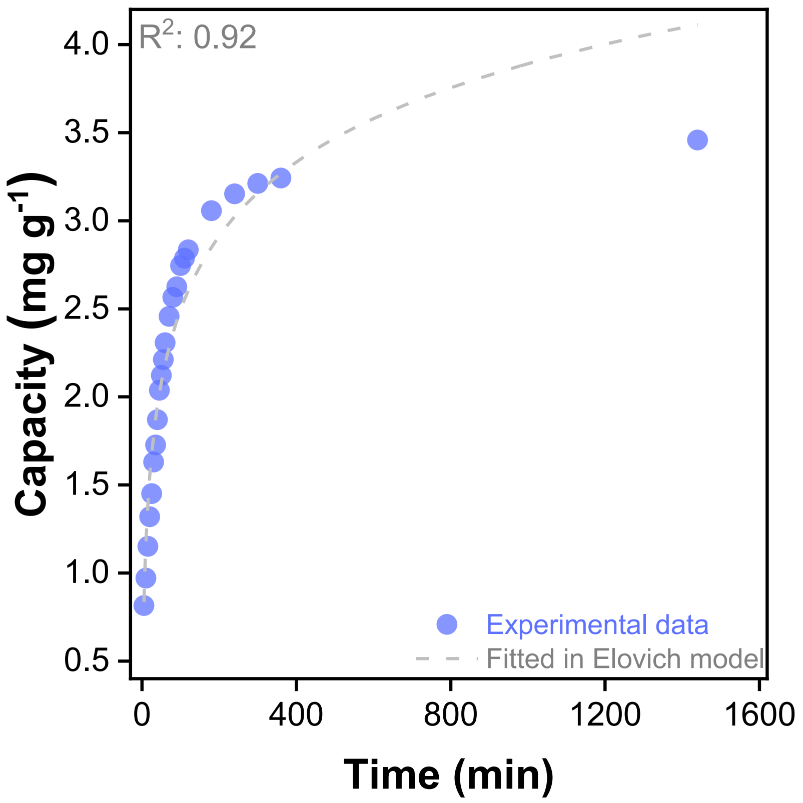

Figure S46. Adsorption kinetic for the capture of Acridine Yellow (initial concentration: 6 ppm) within the BCN-11_42 hydrogel fitted to the Elovich model.

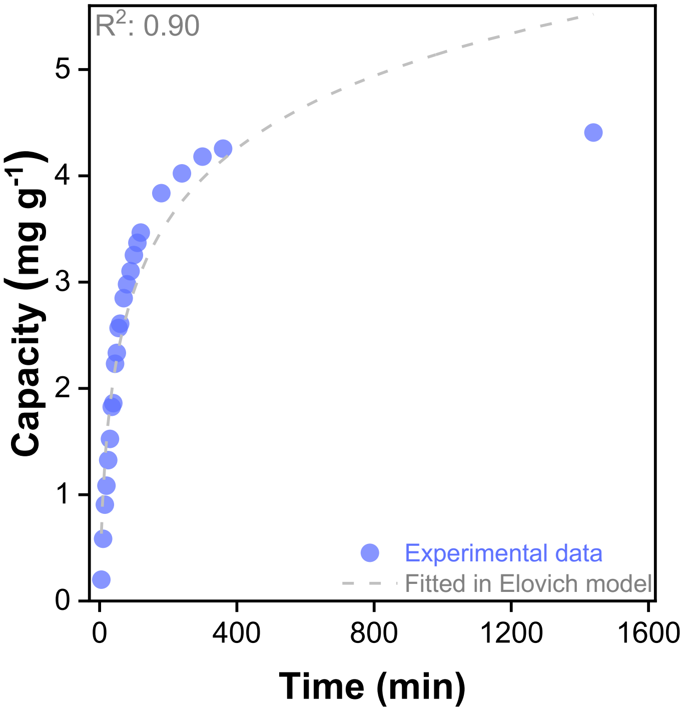

Figure S47. Adsorption kinetic for the capture of Acridine Yellow (initial concentration: 7 ppm) within the BCN-11_42 hydrogel fitted to the Elovich model.

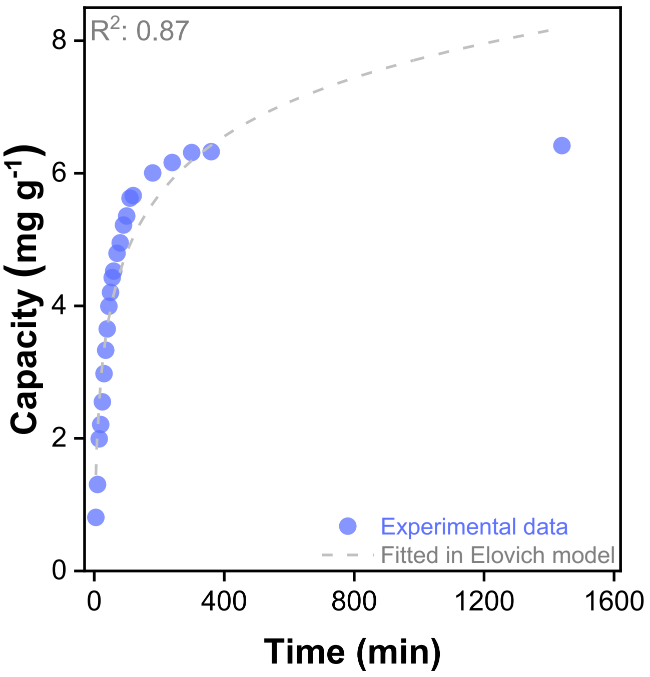

Figure S48. Adsorption kinetic for the capture of Acridine Yellow (initial concentration: 10 ppm) within the BCN-11_42 hydrogel fitted to the Elovich model.

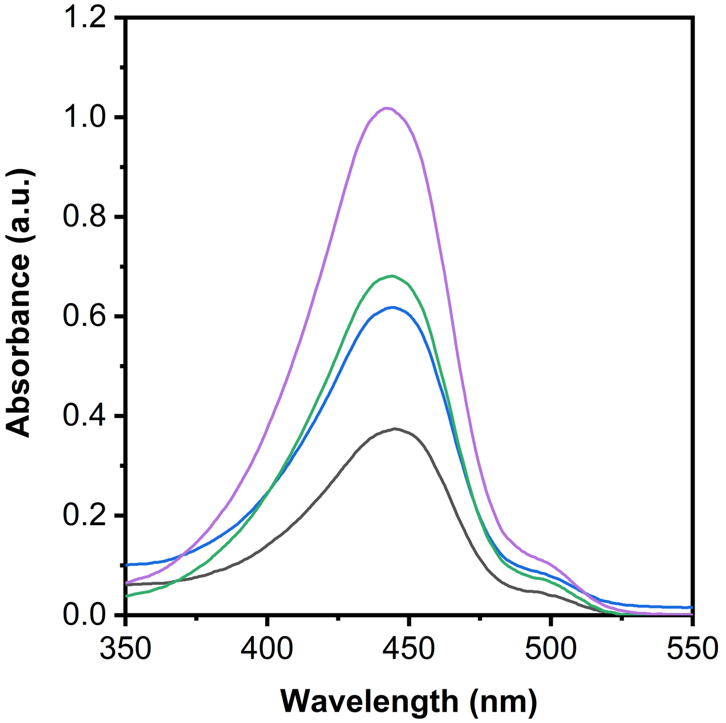

Figure S49. UV-Vis absorption spectra of Acridine Yellow at different concentrations. The spectrums were measured for initial solutions at 4 ppm (dark grey), 6 ppm (blue), 7 ppm (green), and 10 ppm (pink).

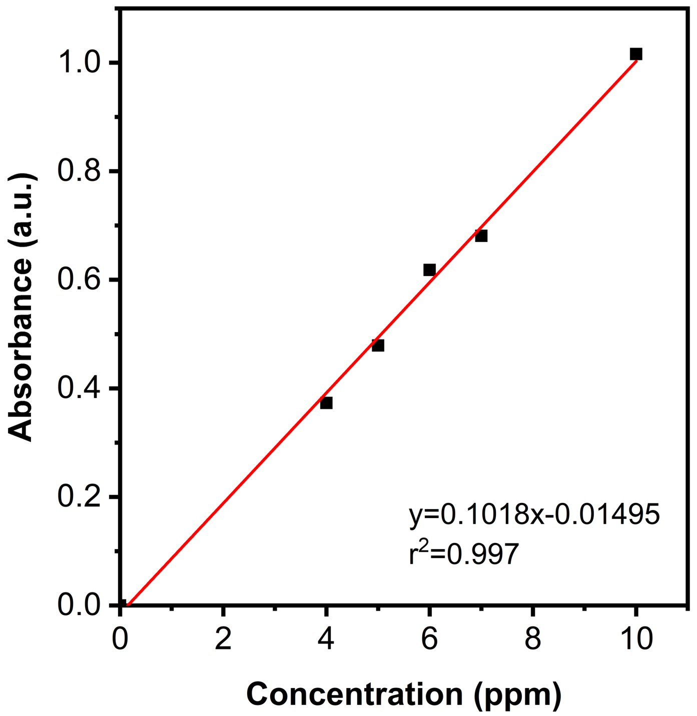

Figure S50. Calibration curve for Acridine Yellow created from the initial absorbance at 444 nm for the different concentrations measured.

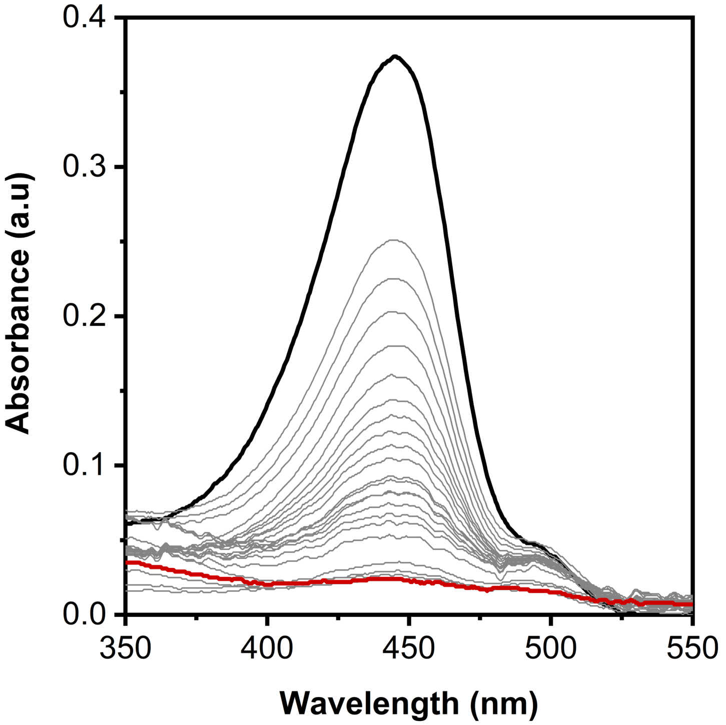

Figure S51. Decrease of the absorbance intensity of the UV-vis spectrum of the 4-ppm solution of Acridine Yellow when incubated with the BCN-11_42 hydrogel over time.

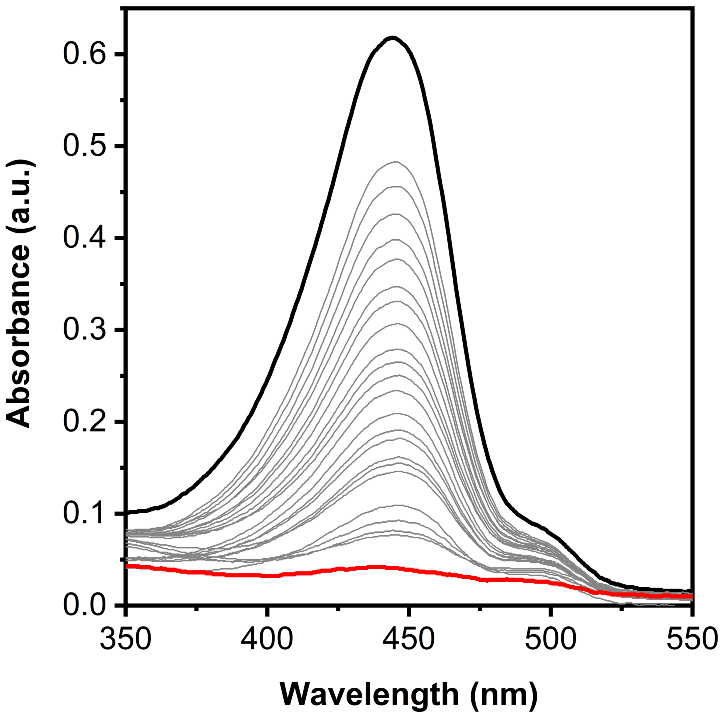

Figure S52. Decrease of the absorbance intensity of the UV-vis spectrum of the 6-ppm solution of Acridine Yellow when incubated with the BCN-11_42 hydrogel over time.

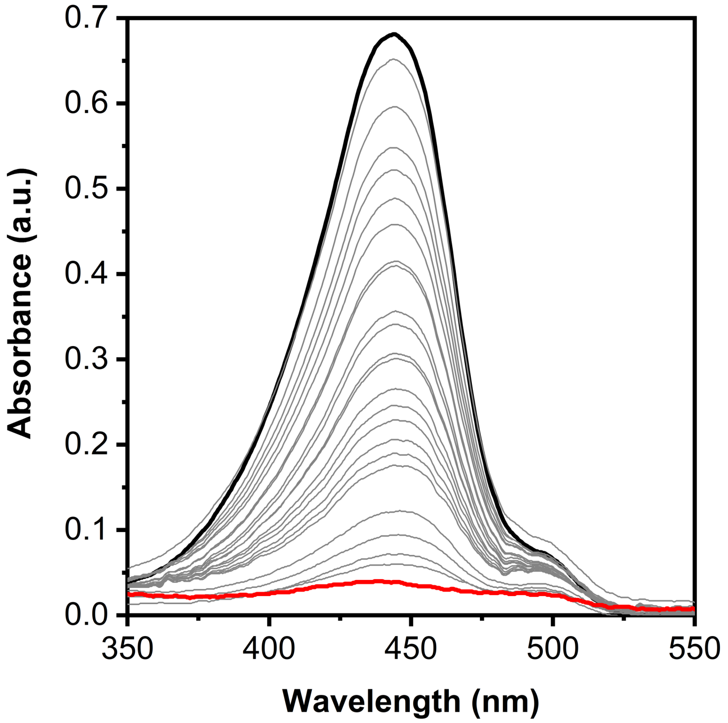

Figure S53. Decrease of the absorbance intensity of the UV-vis spectrum of the 7-ppm solution of Acridine Yellow when incubated with the BCN-11_42 hydrogel over time.

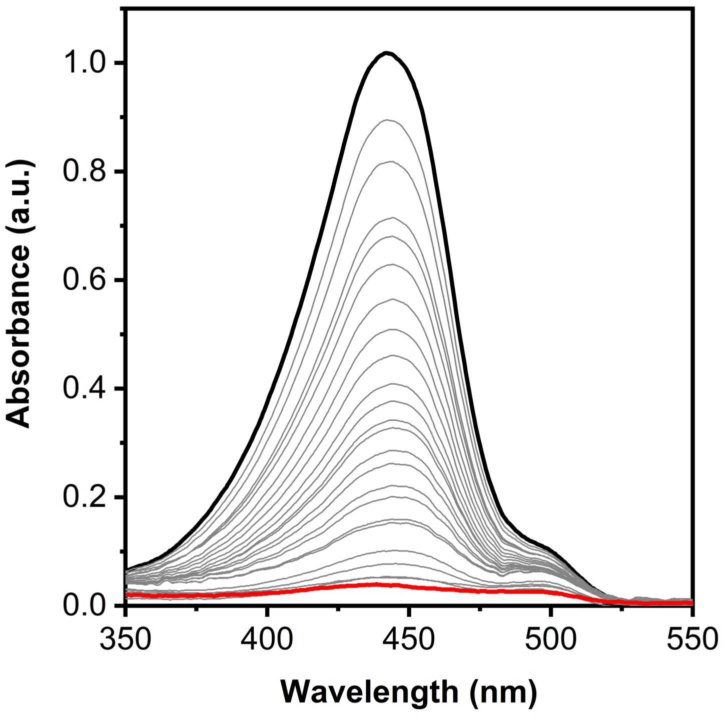

Figure S54. Decrease of the absorbance intensity of the UV-vis spectrum of the 10-ppm solution of Acridine Yellow when incubated with the BCN-11_42 hydrogel over time.

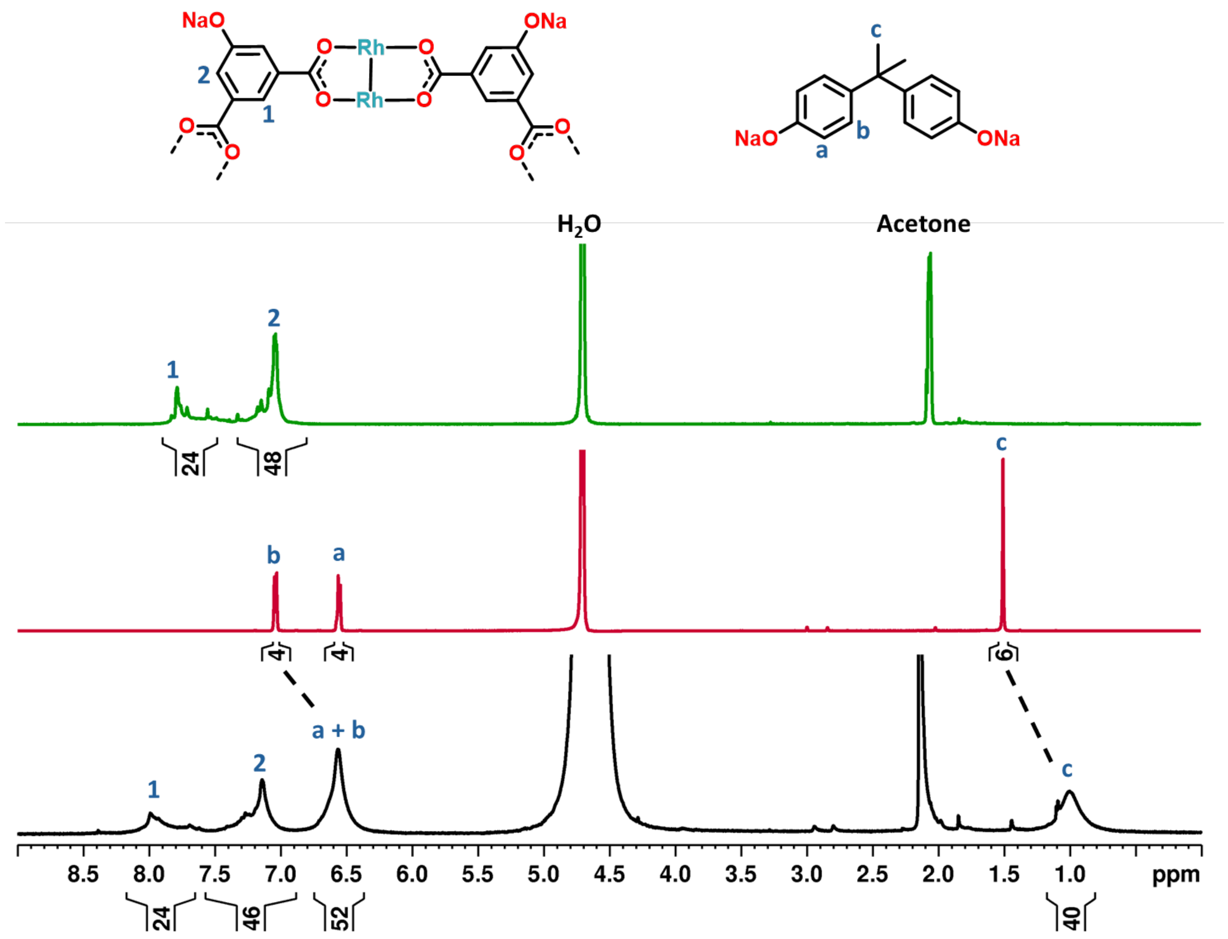

Figure S55. ^{1}H-NMR spectra (500 MHz, 25ºC) in D_2O of ONa-MOP (green), BPA (red) and a mixture of ONa-MOP and 6 mol. eq. of BPA (black) (pD = 11). Note that the shift and broadness of signals a, b and c confirm the interaction between ONa-MOP and BPA.

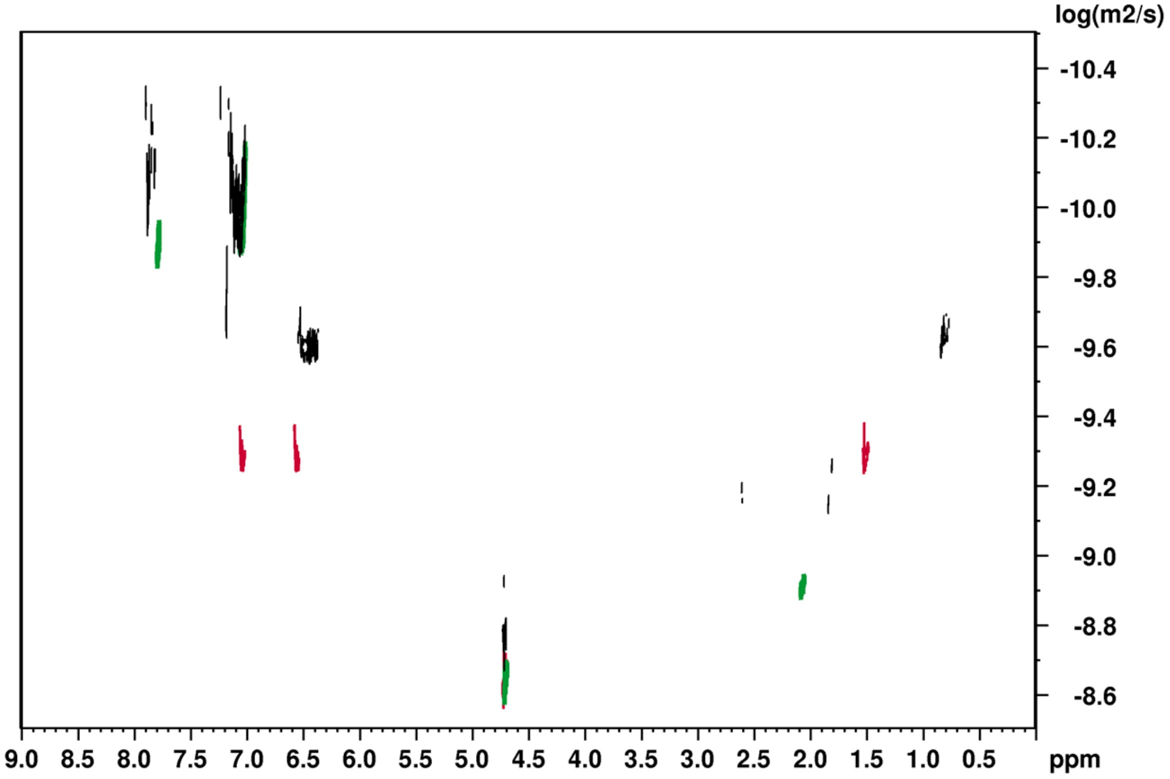

Figure S56. DOSY-NMR spectra (500 MHz, 25ºC) in D_2O of ONa-MOP (green), BPA (red) and a mixture of ONa-MOP and 6 mol. eq. of BPA (black) (pD = 11). The decrease of the diffusion coefficient of the signals ascribed to BPA upon mixing with ONa-RhMOP confirms the interaction between ONa-MOP and BPA.

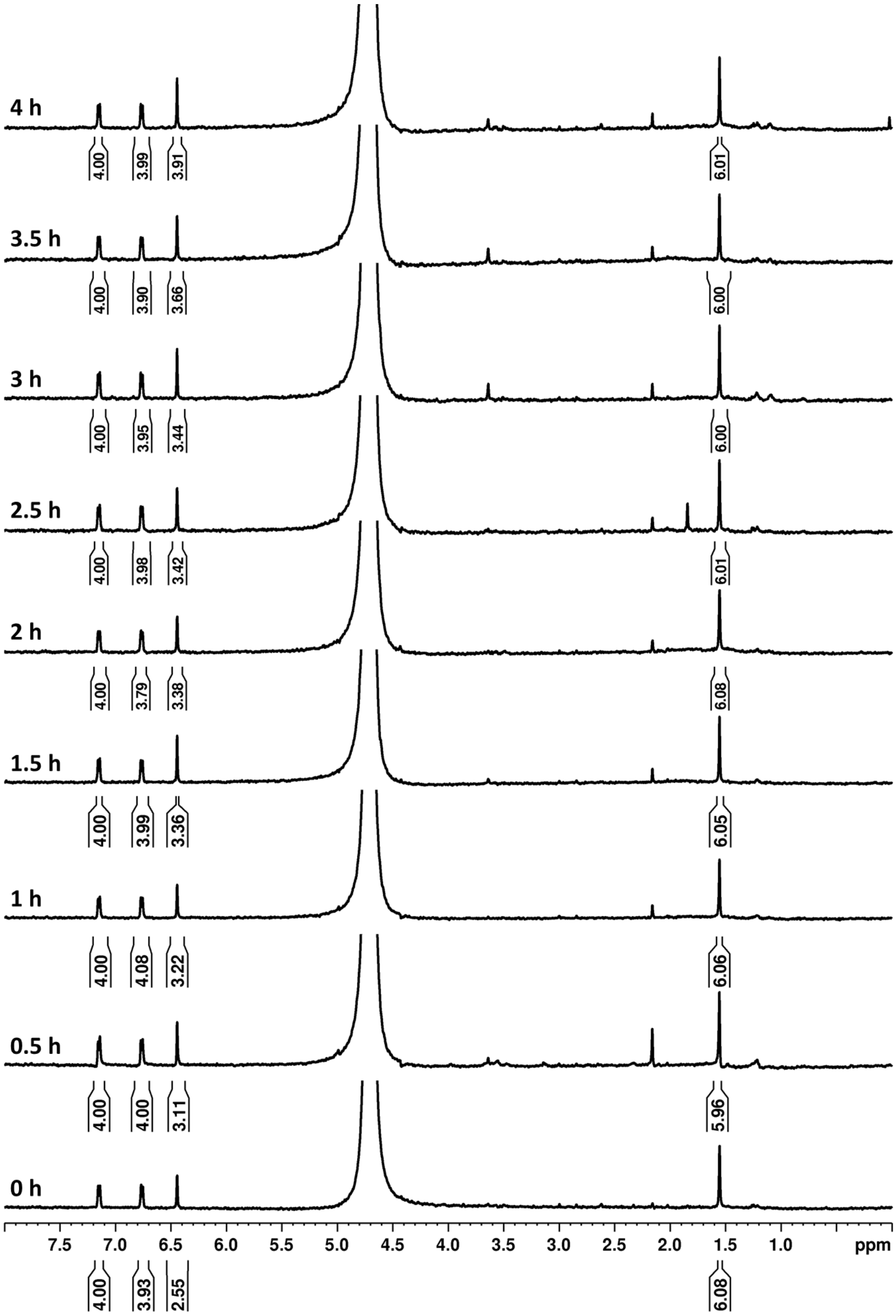

Figure S57. ^{1}H-NMR spectra (500 MHz, 25ºC) of the remaining BPA (initial concentration of 40 ppm) in solution at different times when incubated with BCN-11_42 hydrogel over the first 4 h. Note that fumaric acid (0.02 µmol) was added to quantify the remaining BPA.

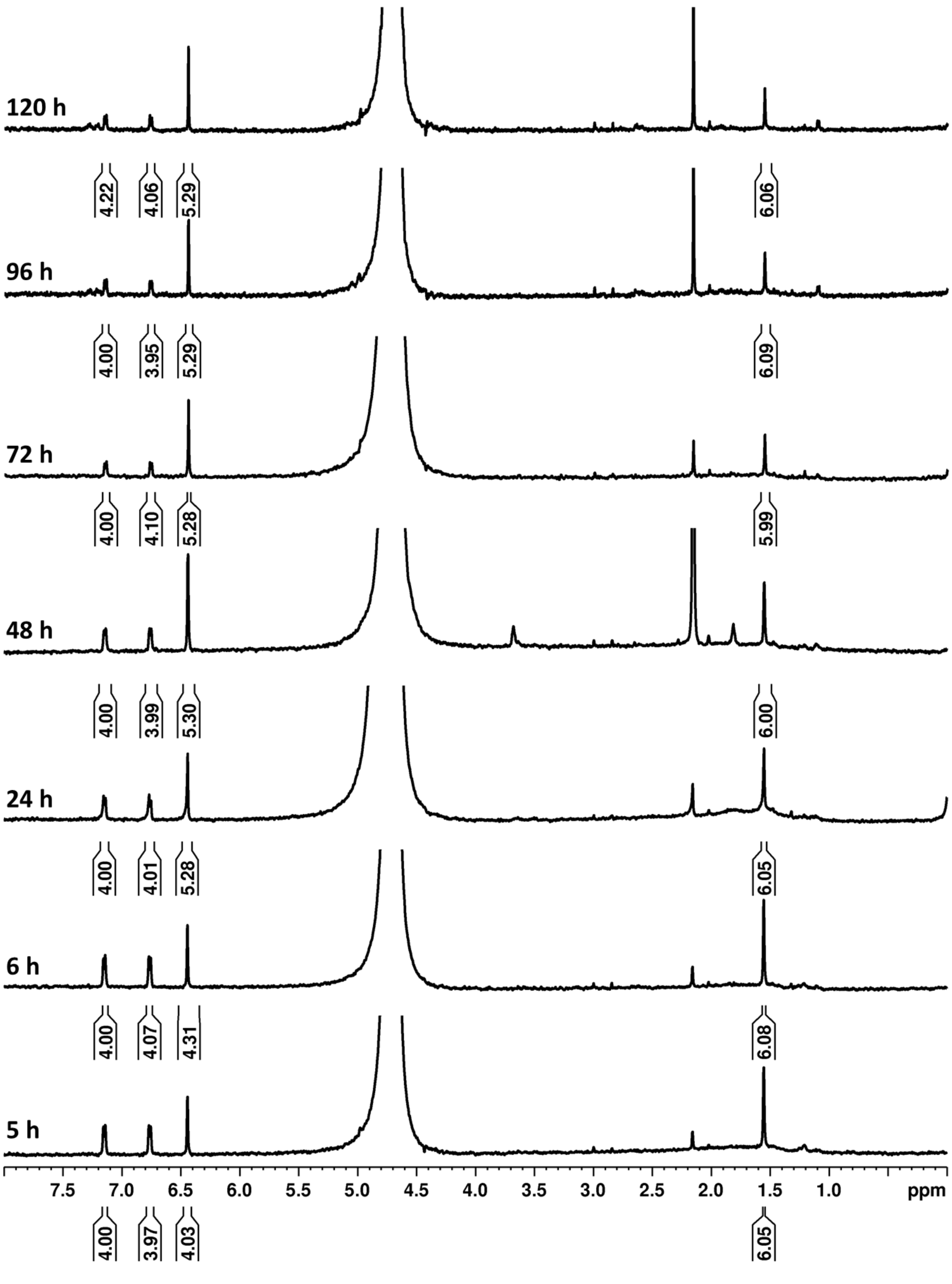

Figure S58. ^{1}H-NMR spectra (500 MHz, 25ºC) of the remaining BPA (initial concentration of 40 ppm) in solution at different times when incubated with BCN-11_42 hydrogel over 5 – 120 h. Note that fumaric acid (0.02 µmol) was added to quantify the remaining BPA.

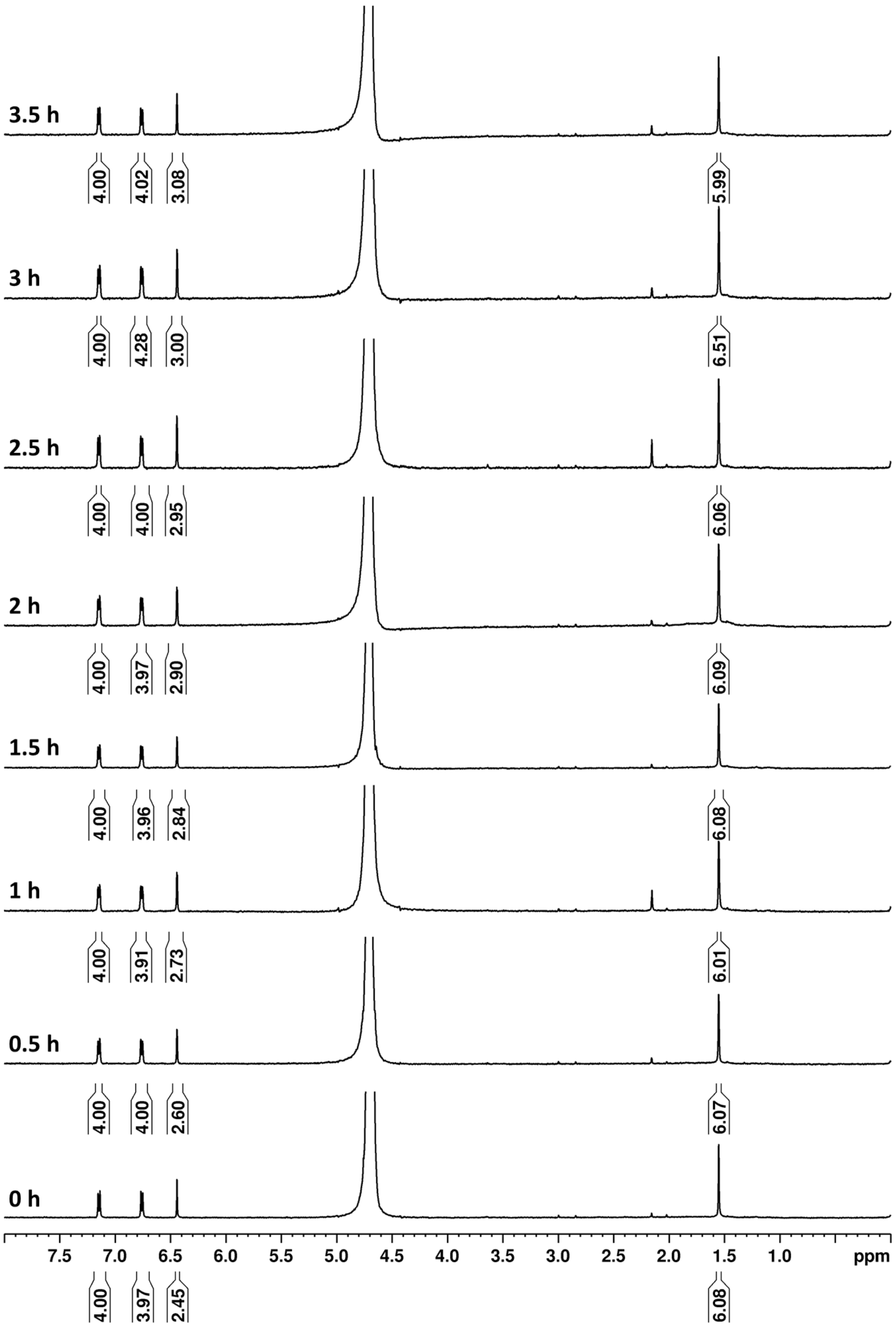

Figure S59. ^{1}H-NMR spectra (500 MHz, 25ºC) of the remaining BPA (initial concentration of 80 ppm) in solution at different times when incubated with BCN-11_42 hydrogel over the first 3.5 h. Note that fumaric acid (0.02 μmol) was added to quantify the remaining BPA.

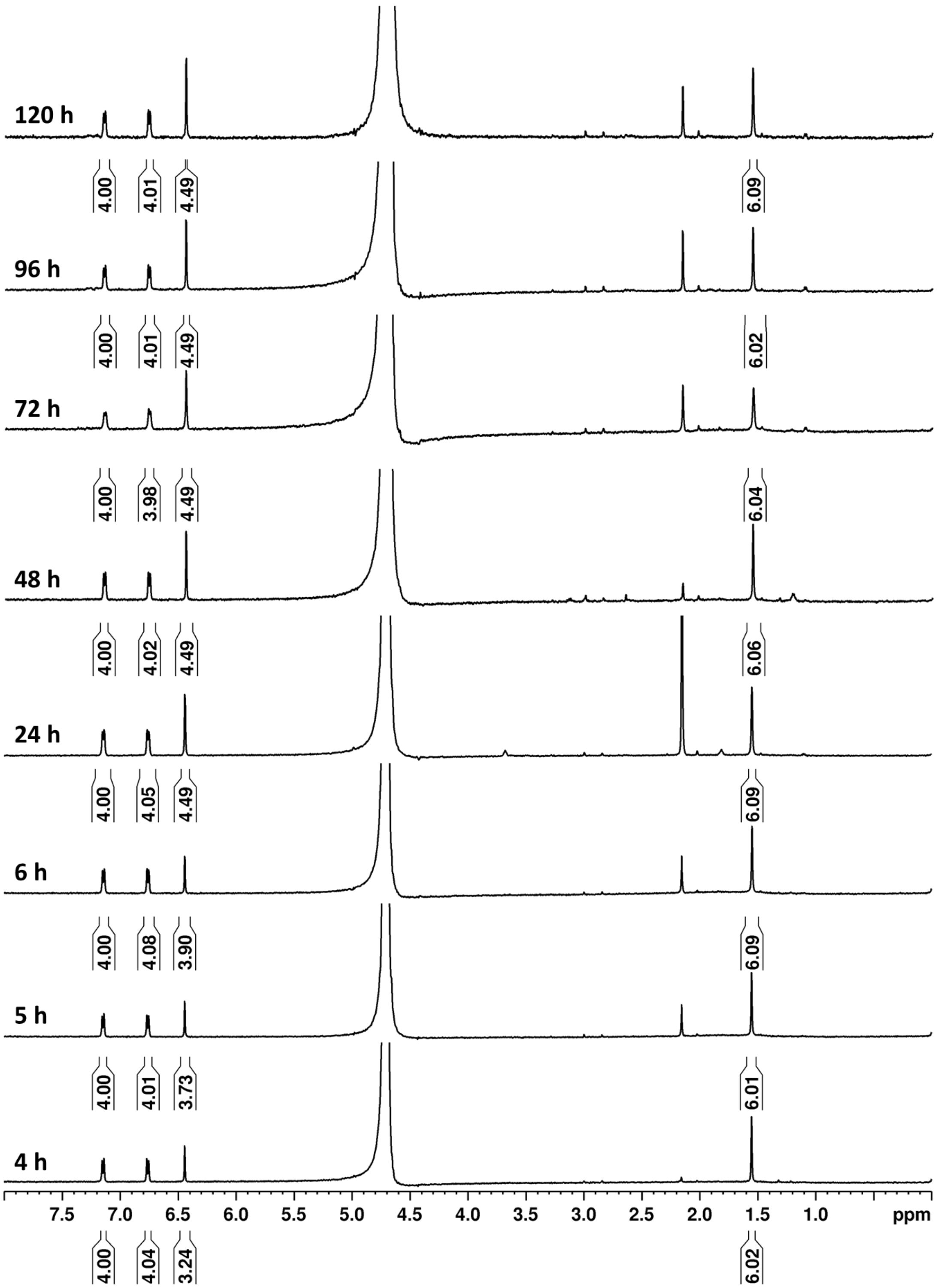

Figure S60. ^{1}H-NMR spectra (500 MHz, 25ºC) of the remaining BPA (initial concentration of 80 ppm) in solution at different times when incubated with BCN-11_42 hydrogel over 4 – 120 h. Note that fumaric acid (0.02 μmol) was added to quantify the remaining BPA.

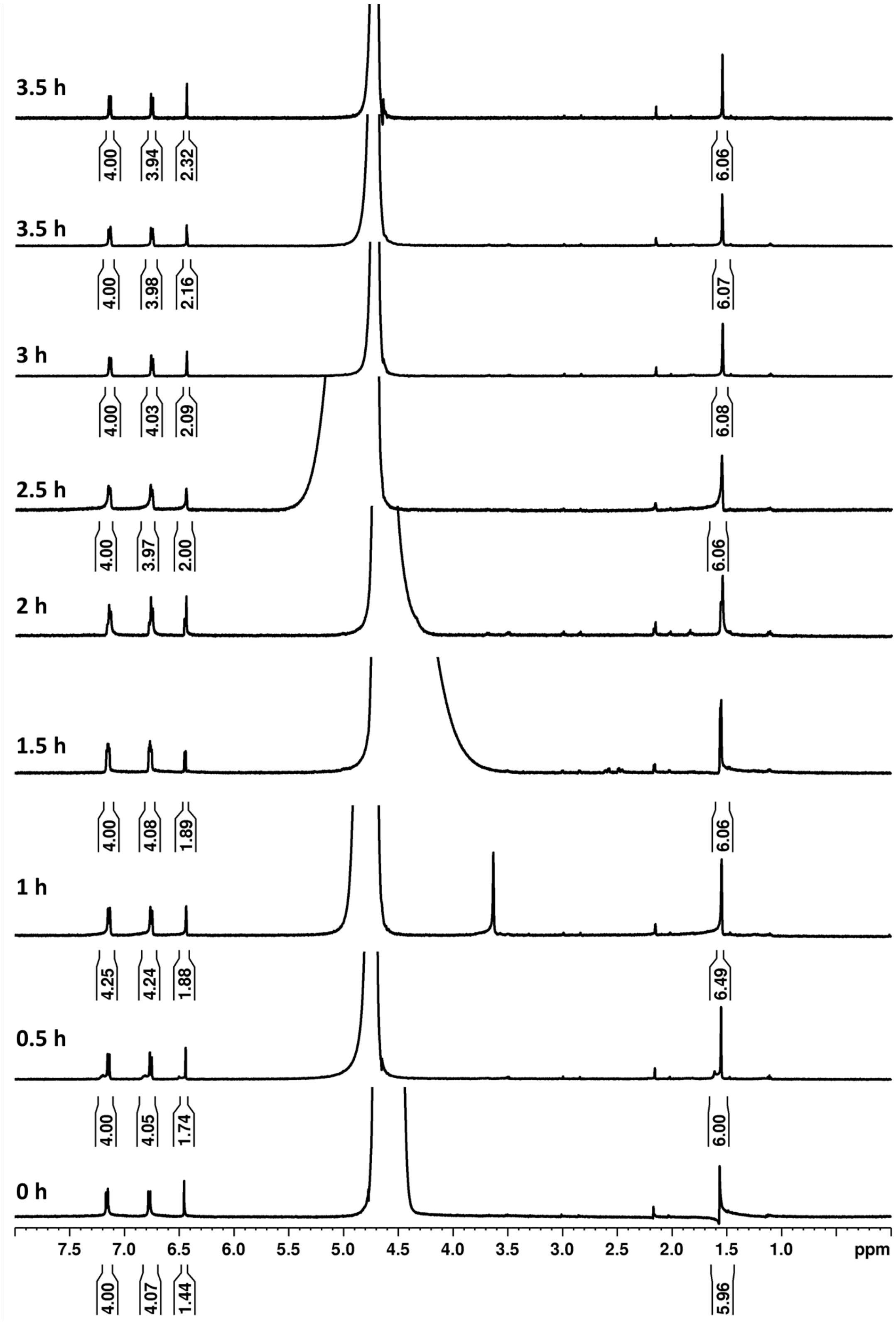

Figure S61. ^{1}H-NMR spectra (500 MHz, 25ºC) of the remaining BPA (initial concentration of 200 ppm) in solution at different times when incubated with BCN-11_42 hydrogel over the first 3.5 h. Note that fumaric acid (0.02 µmol) was added to quantify the remaining BPA.

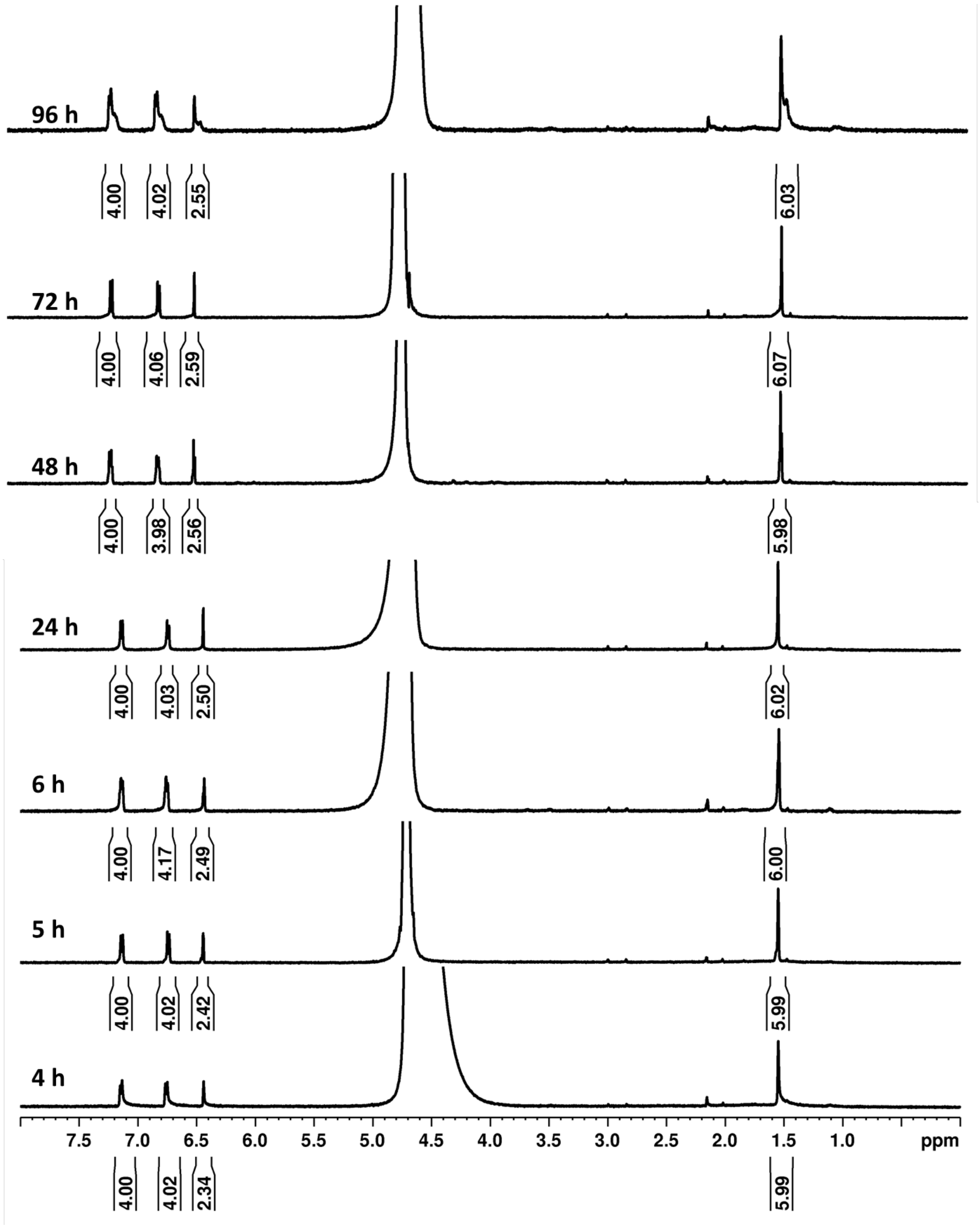

Figure S62. ^{1}H-NMR spectra (500 MHz, 25ºC) of the remaining BPA (initial concentration of 200 ppm) in solution at different times when incubated with BCN-11_42 hydrogel over 4 – 96 h. Note that fumaric acid (0.02 µmol) was added to quantify the remaining BPA.

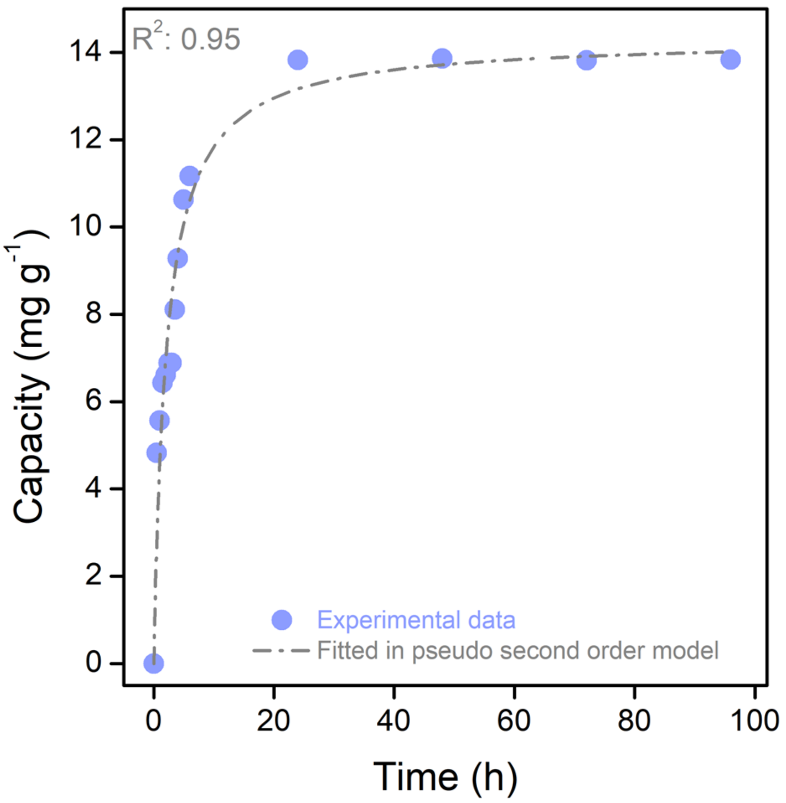

Figure S63. Adsorption kinetic for the capture of BPA (initial concentration: 40 ppm) within the BCN-11_42 hydrogel fitted in pseudo second-order kinetic model.

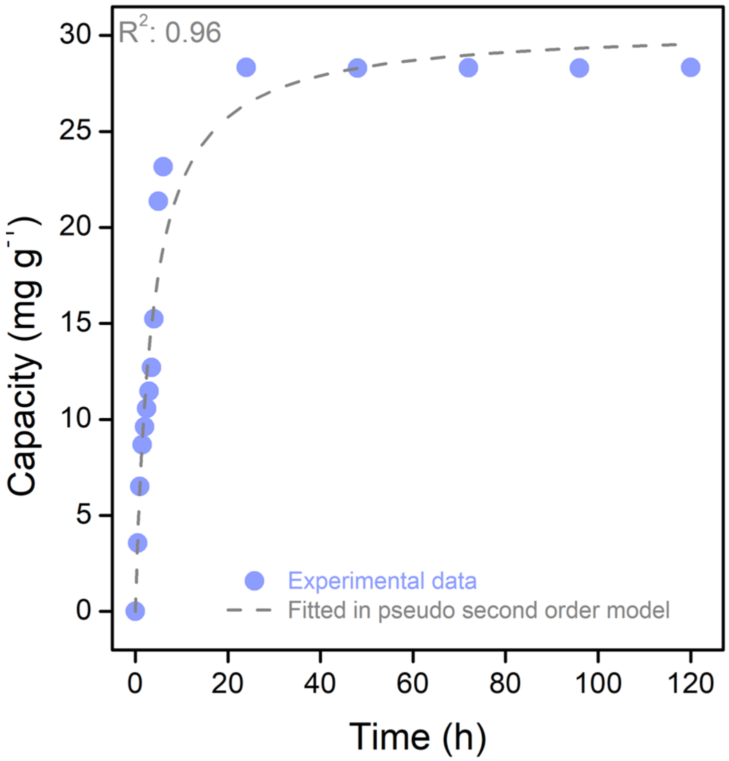

Figure S64. Adsorption kinetic for the capture of BPA (initial concentration: 80 ppm) within the BCN-11_42 hydrogel fitted in pseudo second-order kinetic model.

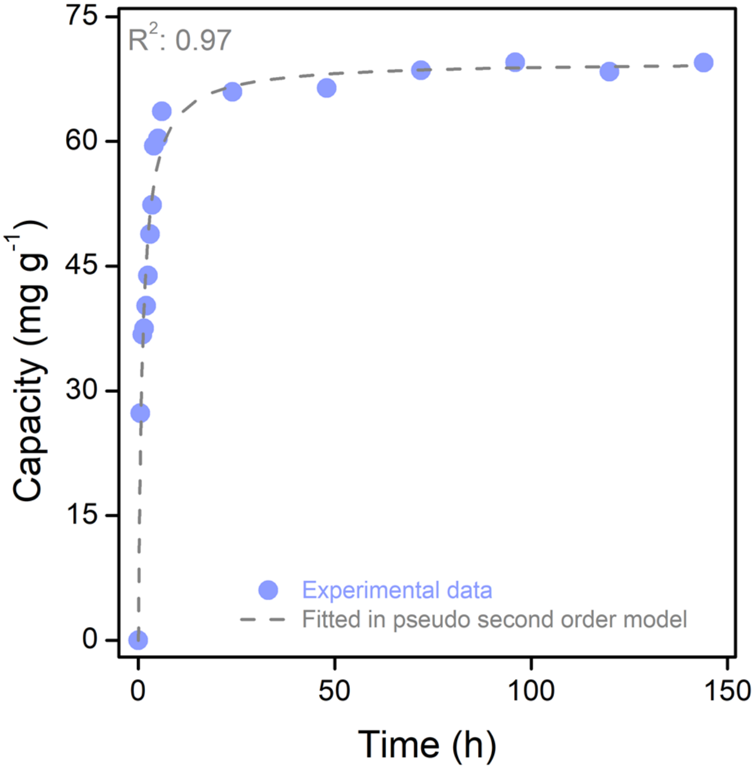

Figure S65. Adsorption kinetic for the capture of BPA (initial concentration: 200 ppm) within the BCN-11_42 hydrogel fitted in pseudo second-order kinetic model.

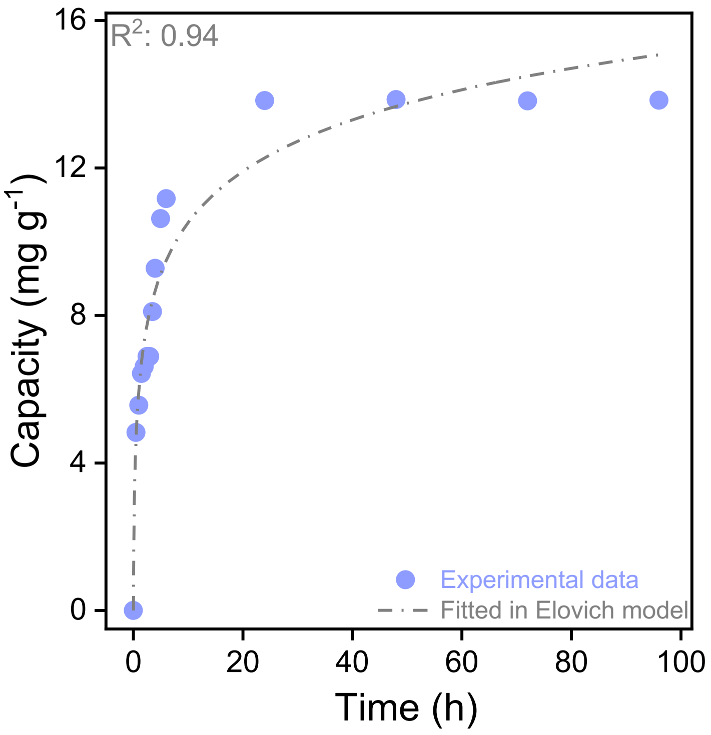

Figure S66. Adsorption kinetic for the capture of BPA (initial concentration: 40 ppm) within the BCN-11_42 hydrogel fitted in the Elovich model.

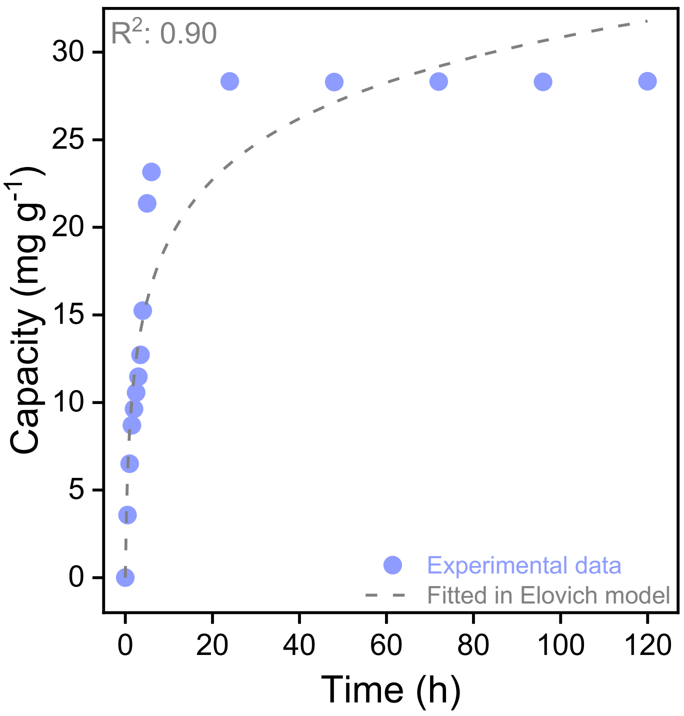

Figure S67. Adsorption kinetic for the capture of BPA (initial concentration: 80 ppm) within the BCN-11_42 hydrogel fitted in the Elovich model.

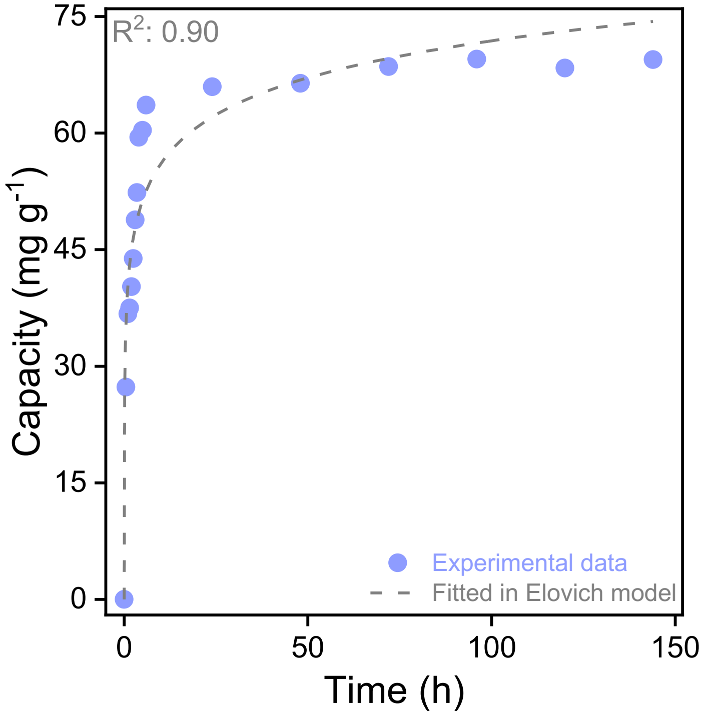

Figure S68. Adsorption kinetic for the capture of BPA (initial concentration: 200 ppm) within the BCN-11_42 hydrogel fitted in the Elovich model.

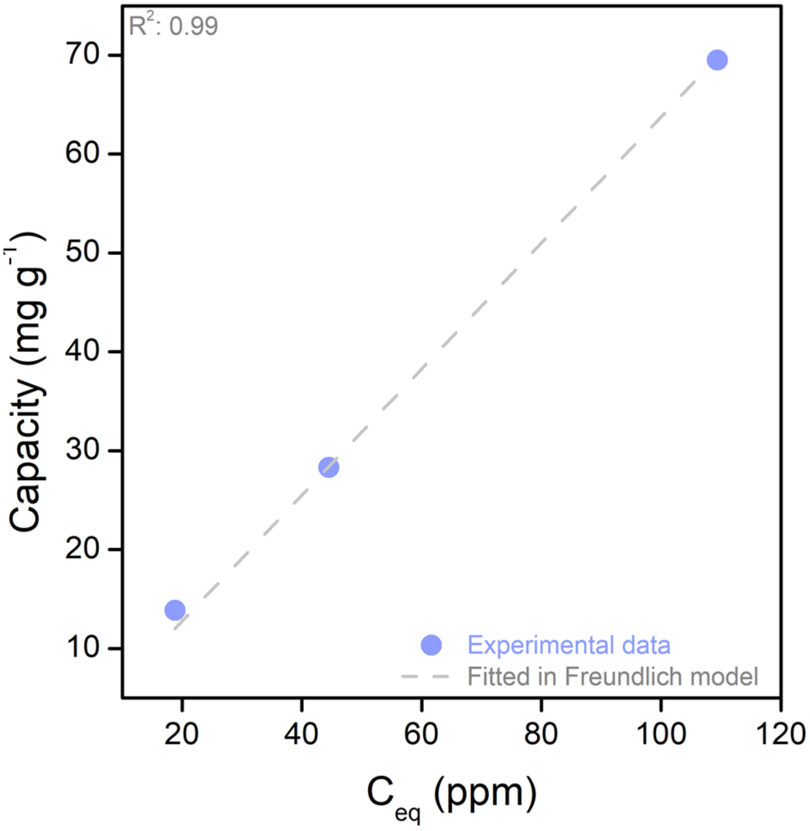

Figure S69. Fitting of Freundlich adsorption isotherm models for the removal of BPA with the BCN-11_42 hydrogel.

Table S5. Table of relevant adsorbents for the pollutants studied in this work.

ENTRY	POLLUTANT	ADSORBENT MATERIAL	ADSORPTION CAPACITY (MG/G)	ADSORPTION MODEL	REF.
**1**	Bisphenol A	BCN-11_42 hydrogel	69.4	Freundlich model	This work
**2**	Bisphenol A	Banana Bunch Biomass	20.98	Langmuir model	22
**3**	Bisphenol A	Coconut Bunch Biomass	21.72	Langmuir model	22
**4**	Bisphenol A	Surfactant-modified natural zeolite	6.9	Tempkin model	23
**5**	Bisphenol A	Empty fruit bunch activated carbon	41.98	Langmuir model	24
**6**	Bisphenol A	EGIS/CPAB modified bentonite	119.88	Langmuir model	25
**7**	Bisphenol A	CST modified Bentonite	77.36	Langmuir model	26
**8**	Bisphenol A	nCDp/DT-Fe@MM	25.44	Freundlich model	27
**9**	Bisphenol A	HTAB modified Bentonite	10.449	Freundlich model	28
**10**	Bisphenol A	Spectrogel® type-C	55.77	Freundlich model	29
**11**	Acridine yellow	BCN-11_42 hydrogel	5.92	Freundlich model	This work
**12**	Acridine yellow G	Functionalized graphene nanoplatelets/modified polybutadiene hybrid composite (FGNPs/MPB)	22.9 (Maximum adsorption capacity from the Langmuir model)	Langmuir model	30

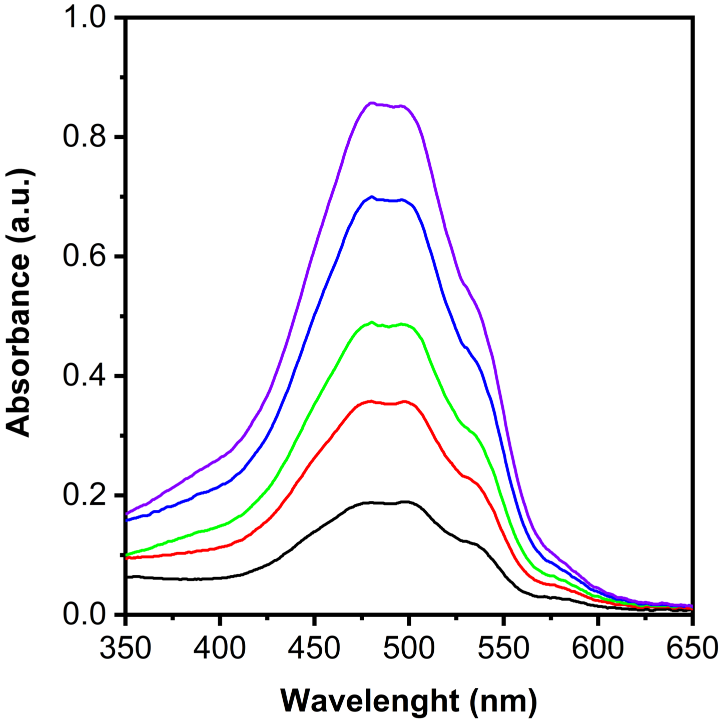

Figure S70. UV-vis of doxorubicin in the digestion solution without DNase I at a concentration of 0.05 mg/mL (purple), 0.04 mg/mL (blue), 0.03 mg/mL (green), 0.02 mg/mL (red) and 0.01 mg/mL (black) used for the calibration curve.

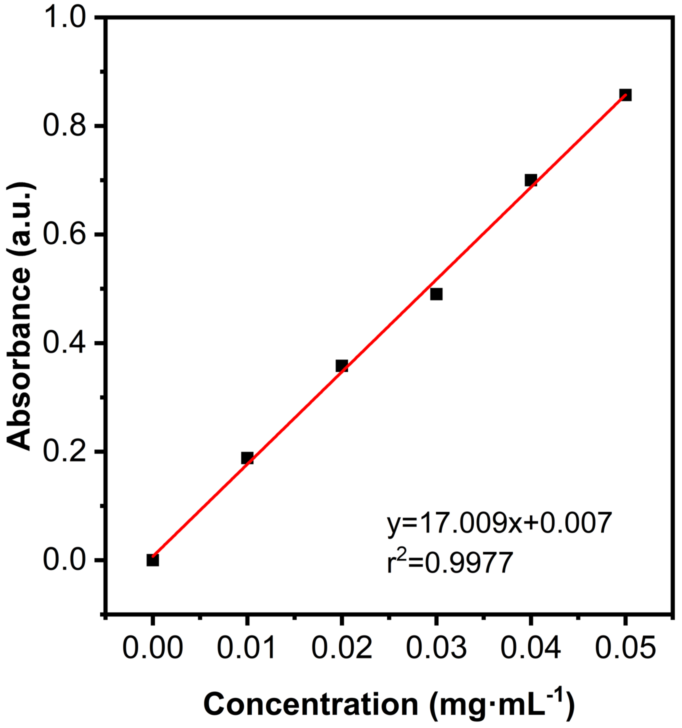

Figure S71. Calibration curve created from the doxorubicin standard solutions in the digestion media without DNase I. The maximum at 480 nm of the UV-Vis spectra of doxorubicin was used for the calibration curve.

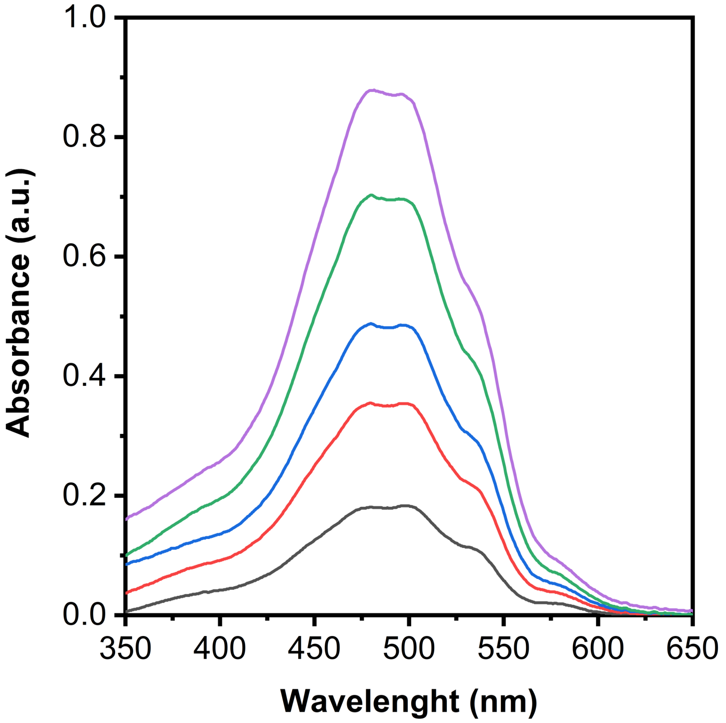

Figure S72. UV-vis of doxorubicin in the digestion solution containing DNase I at a concentration of 0.05 mg/mL (purple), 0.04 mg/mL (blue), 0.03 mg/mL (green), 0.02 mg/mL (red) and 0.01 mg/mL (black) used for the calibration curve.

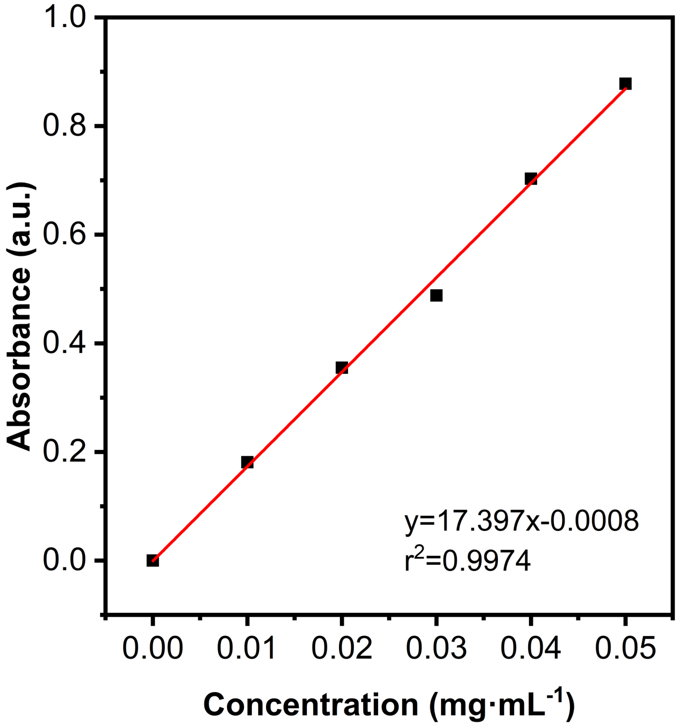

Figure S73. Calibration curve created from the doxorubicin standard solutions in the digestion media containing DNase I. The maximum at 480 nm of the UV-Vis spectra of doxorubicin was used for the calibration curve.

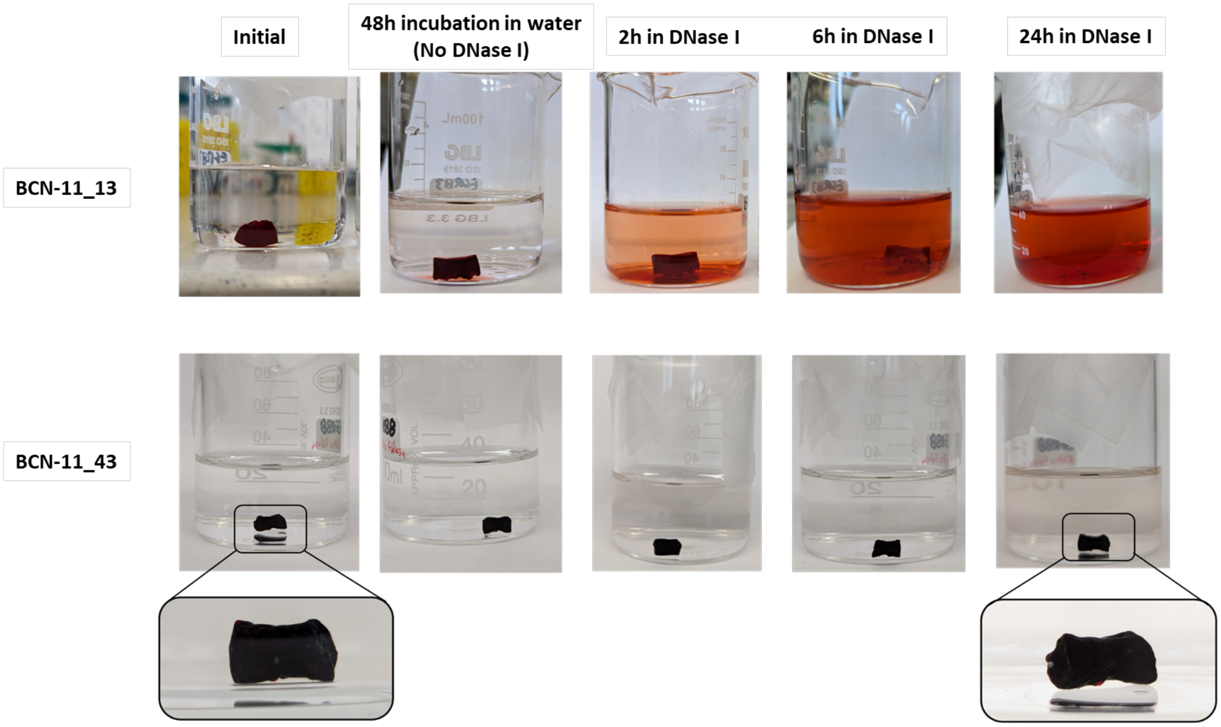

Figure S74. Photographs of the degradation process of BCN-11_13 and BCN-11_43 loaded with doxorubicin in the absence and presence of DNase I.

S3. References

(1) Rondeau EB, Christensen KA, Sakhrani D, Biagi CA, Wetklo M, Johnson HA, Despins CA, Leggatt RA, Minkley DR, Withler RE, et al. Genome assembly, transcriptome and SNP database for chum salmon (Oncorhynchus keta). In.: Cold Spring Harbor Laboratory; 2021. https://doi.org/10.1101/2021.12.27.474290.

(2) Osterrieth, J. W. M.; Rampersad, J.; Madden, D.; Rampal, N.; Skoric, L.; Connolly, B.; Allendorf, M. D.; Stavila, V.; Snider, J. L.; Ameloot, R.; Marreiros, J.; Ania, C.; Azevedo, D.; Vilarrasa-Garcia, E.; Santos, B. F.; Bu, X.-H.; Chang, Z.; Bunzen, H.; Champness, N. R.; Griffin, S. L.; Chen, B.; Lin, R.-B.; Coasne, B.; Cohen, S.; Moreton, J. C.; Colón, Y. J.; Chen, L.; Clowes, R.; Coudert, F.-X.; Cui, Y.; Hou, B.; D'Alessandro, D. M.; Doheny, P. W.; Dincă, M.; Sun, C.; Doonan, C.; Huxley, M. T.; Evans, J. D.; Falcaro, P.; Ricco, R.; Farha, O.; Idrees, K. B.; Islamoglu, T.; Feng, P.; Yang, H.; Forgan, R. S.; Bara, D.; Furukawa, S.; Sanchez, E.; Gascon, J.; Telalović, S.; Ghosh, S. K.; Mukherjee, S.; Hill, M. R.; Sadiq, M. M.; Horcajada, P.; Salcedo-Abraira, P.; Kaneko, K.; Kukobat, R.; Kenvin, J.; Keskin, S.; Kitagawa, S.; Otake, K.; Lively, R. P.; DeWitt, S. J. A.; Llewellyn, P.; Lotsch, B. V.; Emmerling, S. T.; Pütz, A. M.; Martí-Gastaldo, C.; Padial, N. M.; García-Martínez, J.; Linares, N.; Maspoch, D.; Suárez del Pino, J. A.; Moghadam, P.; Oktavian, R.; Morris, R. E.; Wheatley, P. S.; Navarro, J.; Petit, C.; Danaci, D.; Rosseinsky, M. J.; Katsoulidis, A. P.; Schröder, M.; Han, X.; Yang, S.; Serre, C.; Mouchaham, G.; Sholl, D. S.; Thyagarajan, R.; Siderius, D.; Snurr, R. Q.; Goncalves, R. B.; Telfer, S.; Lee, S. J.; Ting, V. P.; Rowlandson, J. L.; Uemura, T.; Iiyuka, T.; van der Veen, M. A.; Rega, D.; Van Speybroeck, V.; Rogge, S. M. J.; Lamaire, A.; Walton, K. S.; Bingel, L. W.; Wuttke, S.; Andreo, J.; Yaghi, O.; Zhang, B.; Yavuz, C. T.; Nguyen, T. S.; Zamora, F.; Montoro, C.; Zhou, H.; Kirchon, A.; Fairen-Jimenez, D. How Reproducible Are Surface Areas Calculated from the BET Equation? *Adv. Mater*. **2022**, 34 (27), 2201502.

(3) Carné-Sánchez, A.; Albalad, J.; Grancha, T.; Imaz, I.; Juan-huix, J.; Larpent, P.; Furukawa, S.; Maspoch, D. Postsynthetic Covalent and Coordination Functionalization of Rhodium(II)-Based Metal–Organic Polyhedra. *J. Am. Chem. Soc.* **2019**, *141* (9), 4094–4102.

(4) Lachance-Brais, C.; Rammal, M:; Asohan, J.; Katolik, A.; Luo, X.; Saliba, D.; Jonderian, A.; Damha, M. J.; Harrington, M. J.; Sleiman, H. F. Small Molecule-Templated DNA Hydrogel with Record Stiffness Integrates and Releases DNA Nanostructures and Gene Silencing Nucleic Acids. *Adv. Sci.* **2023**, 10 (12), 2205713

(5) Shi, J.; Zhu, C.; Li, Q.; Li, Y.; Chen, L.; Yang, B.; Xu, J-F.; Dong, Y.; Mao, C.; Liu, D. Kinetically Interlocking Multiple-Units Polymerization of DNA Double Crossover and Its Application in Hydrogel Formation. *Macromol. Rapid Commun.* **2021**, 42, 2100182.

(6) Xing, Y.; Cheng, E.; Yang, Y.; Chen, P.; Zhang, T.; Sun, Y.; Yang, Z.; Liu, D. Self-assembled DNA hydrogels with designable thermal and enzymatic responsiveness. *Adv. Mater*. **2011**, 23 (9), 1117-1121.

(7) Pan, W.; Wen, H.; Niu, L.; Su, C.; Liu, C.; Zhao, J.; Mao, D.; Liang, D. Effects of chain flexibility on the properties of DNA hydrogels. *Soft Matter*, **2016**,12, 5537-5541.

(8) Jiang, H.; Pan, V.; Vivek, S.; Weeks, E. R.; Ke, Y. Programmable DNA Hydrogels Assembled from Multidomain DNA Strands. *Chem. Eur. J.* **2016**, 17 (12), 1156-1162.

(9) Yang, B.; Zhao, Z.; Pan, Y.; Xie, J.; Zhou, B.; Li, Y.; Dong, Y.; Liu, D. Shear-Thinning and Designable Responsive Supramolecular DNA Hydrogels Based on Chemically Branched DNA. *ACS Appl. Mater. Interfaces* **2021**, 13, 41, 48414–48422.

(10) Yang, B.; Zhou, B.; Li, C.; Li, X.; Shi, Z.; Li, Y.; Zhu, C.; Li, X.; Hua, Y.; Pan, Y.; He, J.; Cao, T.; Sun, Y.; Liu, W. Ge, M.; Yang, Y. R.; Dong, Y.; Liu, D. *Angew. Chem. Int. Ed*. **2022**, 61, e202202520.

(11) Ma, X.; Yang, Z.; Wang, Y.; Zhang, G.; Shao, Y.; Jia, H.; Cao, T.; Wang, R.; Liu, D. Remote Controlling DNA Hydrogel by Magnetic Field. *ACS Appl. Mater. Interfaces* **2017**, 9, 3, 1995–2000.

(12) Tang, J.; Ou, J.; Zhu, C.; Yao, C.; Yang, D. Flash Synthesis of DNA Hydrogel via Supramacromolecular Assembly of DNA Chains and Upconversion Nanoparticles for Cell Engineering. *Adv. Funct. Mater*. **2022**, 32, 2107267.

(13) Eguchi, Y.; Kato, T.; Tanakaa, T.; Maruyama, T. A DNA–gold nanoparticle hybrid hydrogel network prepared by enzymatic reaction. *Chem. Commun*., **2017**,53, 5802-5805

(14) Sarma, S.; Thakur, N.; Varshney, N.; Jha, H. C.; Sarma, T. K. Chromatin inspired bio-condensation between biomass DNA and guanosine monophosphate produces all-nucleic hydrogel as a hydrotropic drug carrier. *Commun Chem.* **2024**, 7, 261

(15) Nayak, S.; Kumar, P.; Shankar, R.; Kumar, A.; Mukhopadhyay, A. K.; Mandal, S.; Das, P. Biomass derived self-assembled DNA-dot hydrogels for enhanced bacterial annihilation. *Nanoscale*, **2022**,14, 16097-16109.

(16) Han, J.; Guo, Y.; Wang, H.; Zhang, K.; Yang, D. Sustainable Bioplastic Made from Biomass DNA and Ionomers. *J. Am. Chem. Soc*. **2021**, 143, 46, 19486–19497.

(17) Xu, Y.; Wu, Q.; Sun, Y.; Bai, H.; Shi, G. Three-Dimensional Self-Assembly of Graphene Oxide and DNA into Multifunctional Hydrogels. *ACS Nano* **2010**, 4, 12, 7358–7362.

(18) Basu, S.; Alkiswani, A-R.; Pacelli, S.; Paul, A. Nucleic Acid-Based Dual Cross-Linked Hydrogels for in Situ Tissue Repair via Directional Stem Cell Migration. *ACS Appl. Mater. Interfaces* **2019**, 11, 38, 34621–34633.

(19) Ji, Y.; Kim, T.; Han, D.; Lee, J. B. Self-Healing and Thermal Responsive DNA Bioplastics for On-Demand Degradable Medical Devices. *ACS Materials Lett*. **2024**, 6, 4, 1277–1287.

(20) Basu, S.; Johl, R.; Pacelli, S.; Gehrke, S.; Paul, A. Fabricating Tough Interpenetrating Network Cryogels with DNA as the Primary Network for Biomedical Applications. *ACS Macro Lett*. **2020**, 9, 9, 1230–1236.

(21) Basu, S.; Pacelli, S.; Feng, Y.; Lu, Q.; Wang, J.; Paul. A. Harnessing the Noncovalent Interactions of DNA Backbone with 2D Silicate Nanodisks To Fabricate Injectable Therapeutic Hydrogels. *ACS Nano* **2018**, 12, 10, 9866–9880.
(22) Lazim, Z.M;, Salmiati, Hadibarata, H.; Yusop, Z.; Nazifa, T. H.; Abdullah, N. H.; Nuid, M.; Salim, N. A. A.; Zainuddin, N. A; Ahmad, N. Bisphenol A Removal by Adsorption Using Waste Biomass: Isotherm and Kinetic Studies. *Biointerface Res. Appl. Chem*. **2020**, 11, 8467–8481.
(23) Genç, N.; Kılıçoğlu, Ö.; Narci, A. O. Removal of Bisphenol a Aqueous Solution Using Surfactant-Modified Natural Zeolite: Taguchi's Experimental Design, Adsorption Kinetic, Equilibrium and Thermodynamic Study. *Environ. Technol.* **2017**, 38, 424–432.
(24) Wirasnita, R.; Hadibarata, T.; Yusoff, A. R. M.; Yusop, Z. Removal of Bisphenol A from Aqueous Solution by Activated Carbon Derived from Oil Palm Empty Fruit Bunch. *Water. Air. Soil Pollut*. **2014**, 225, 2148.
(25) Men, X.; Guo, Q.; Meng, B.; Ren, S.; Shen, B. Adsorption of Bisphenol A in Aqueous Solution by Composite Bentonite with Organic Moity. *Microporous Mesoporous Mater*. **2020**, 308, 110450.
(26) Cao, Y.; Zhou, G.; Zhou, R.; Wang, C.; Chi, B.; Wang, Y.; Hua, C.; Qiu, J.; Jin, Y.; Wu, S. Green Synthesis of Reusable Multifunctional γ-Fe2O3/Bentonite Modified by Doped TiO2 Hollow Spherical Nanocomposite for Removal of BPA. *Sci. Total Environ*. **2020**, 708, 134669.
(27) Okon, O. E.; Inam, E. J.; Offiong, N.-A. O.; Akpabio, U. D. Aqueous Adsorptive Removal of Bisphenol A Using Tripartite Magnetic Montmorillonite Composites. *Pollutants* **2022**, 2, 363–387.
(28) Li, Y.; Jin, F.; Wang, C.; Chen, Y.; Wang, Q.; Zhang, W.; Wang, D. Modification of Bentonite with Cationic Surfactant for the Enhanced Retention of Bisphenol A from Landfill Leachate. *Environ. Sci. Pollut. Res*. **2015**, 22, 8618–8628.
(29) De Farias, M. B.; Silva, M. G. C.; Vieira, M. G. A. Adsorption of Bisphenol A from Aqueous Solution onto Organoclay: Experimental Design, Kinetic, Equilibrium and Thermodynamic Study. *Powder Technol*. **2022**, 395, 695–707.
(30) Aliyeva, S. Adsorption of Acridine Yellow G from Aqueous Solutions Using Functionalized Graphene Nanoplatelets/Modified Polybutadiene Hybrid Composite. *J. Chin. Chem. Soc*. **2020**, 67 (11), 2071–2081.
